# Mapping the biosynthetic pathway of a hybrid polyketide-nonribosomal peptide in a metazoan

**DOI:** 10.1038/s41467-021-24682-9

**Published:** 2021-08-13

**Authors:** Likui Feng, Matthew T. Gordon, Ying Liu, Kari B. Basso, Rebecca A. Butcher

**Affiliations:** 1 Department of Chemistry, University of Florida, Gainesville, FL USA; 2 Present Address: Lulu and Anthony Wang Laboratory of Neural Circuits and Behavior, The Rockefeller University, New York, NY USA

**Keywords:** Caenorhabditis elegans, Natural products, Biochemistry, Biosynthesis

## Abstract

Polyketide synthase (PKS) and nonribosomal peptide synthetase (NRPS) hybrid systems typically use complex protein-protein interactions to facilitate direct transfer of intermediates between these multimodular megaenzymes. In the canal-associated neurons (CANs) of *Caenorhabditis elegans*, PKS-1 and NRPS-1 produce the nemamides, the only known hybrid polyketide-nonribosomal peptides biosynthesized by animals, through a poorly understood mechanism. Here, we use genome editing and mass spectrometry to map the roles of individual PKS-1 and NRPS-1 enzymatic domains in nemamide biosynthesis. Furthermore, we show that nemamide biosynthesis requires at least five additional enzymes expressed in the CANs that are encoded by genes distributed across the worm genome. We identify the roles of these enzymes and discover a mechanism for trafficking intermediates between a PKS and an NRPS. Specifically, the enzyme PKAL-1 activates an advanced polyketide intermediate as an adenylate and directly loads it onto a carrier protein in NRPS-1. This trafficking mechanism provides a means by which a PKS-NRPS system can expand its biosynthetic potential and is likely important for the regulation of nemamide biosynthesis.

## Abstract (web summary)

The only known animal polyketide-nonribosomal peptides, the nemamides, are biosynthesized by two megasynthetases in the canal-associated neurons (CANs) of *C. elegans*. Here, the authors map the biosynthetic roles of individual megasynthetase domains and identify additional enzymes in the CANs required for nemamide biosynthesis.

## Introduction

In the past 15 years, it has become clear that animal genomes encode biosynthetic pathways for many microbial-like secondary metabolites^1–11^. Although in some cases these pathways were acquired from microorganisms through horizontal gene transfer, in the majority of cases these pathways are thought to have evolved independently in animals^1,3,7,9–11^. Investigating the rich biochemistry of animals thus has the potential to uncover many important chemical insights and biosynthetic strategies. Furthermore, these studies are poised to reveal how the animal biosynthetic machinery is integrated with the higher-order complexity found in animals, including multiple organelles and tissues, integrated signaling pathways, and complex life-history traits.

**Fig. 1:**
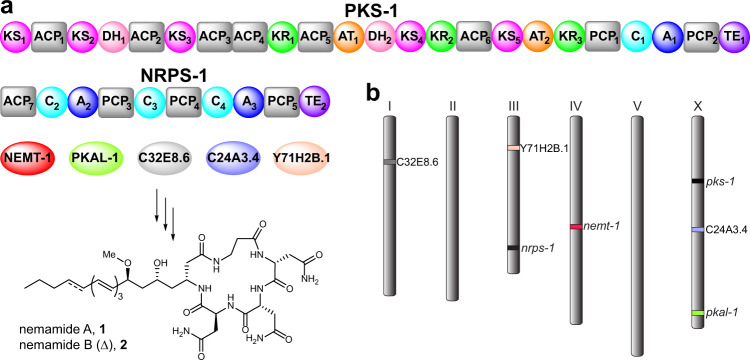
Enzymes required for nemamide biosynthesis. **a** The domain organization of PKS-1 and NRPS-1 is shown, along with five additional free-standing enzymes (NEMT-1, PKAL-1, C32E8.6, C24A3.4, and Y71H2B.1) that were demonstrated in this study to be required for nemamide biosynthesis. To facilitate annotation of the mutant worm strains generated in this study, the enzyme domains have been numbered according to the order of their appearance in PKS-1 and NRPS-1. The ACP_7_ domain was identified and its functional role was confirmed in this study. Domain abbreviations: acyl carrier protein (ACP), acyltransferase (AT), ketosynthase (KS), ketoreductase (KR), dehydratase (DH), peptidyl carrier protein (PCP), adenylation (A), condensation (C), thioesterase (TE). **b** The approximate chromosomal location in *C. elegans* of *pks-1*, *nrps-1*, and the five additional genes demonstrated to be required for nemamide biosynthesis in this study.

Nematodes, in particular, have been shown to have a rich biosynthetic repertoire^12^. In fact, many nematode genomes encode multi-module Type I PKSs and NRPSs for assembly-line-type biosynthesis of polyketides and nonribosomal peptides^3–5,8^. Using the megasynthetases PKS-1 and NRPS-1, the model nematode *Caenorhabditis elegans* has been shown to produce a remarkable class of hybrid polyketide-nonribosomal peptides known as the nemamides in two essential neurons, the canal-associated neurons (CANs) (Fig. 1a)^8^. The nemamides promote the survival of the worm during starvation possibly through their effects on insulin gene expression and in a manner that is at least partially independent of the transcription factor DAF-16/FOXO^8,13^. Since PKS-1 and NRPS-1 homologs are found in most nematode species, including parasitic ones, it is likely that other nematode species produce nemamide-like molecules and that these natural products play a conserved role in nematode biology^8^. However, the biosynthesis of the nemamides, as well as how it is regulated, is poorly understood.

In Type I PKSs and NRPSs, each module is responsible for incorporating a different building block, such as malonyl- or methylmalonyl-CoA in the case of PKSs and proteinogenic or nonproteinogenic amino acids in the case of NRPSs^14,15^. Acyltransferase (AT) domains are responsible for loading building blocks onto acyl carrier proteins (ACPs) in PKSs, while adenylation (A) domains are responsible for loading building blocks onto peptidyl carrier proteins (PCPs) in NRPSs. The building blocks are then linked together by ketosynthase (KS) domains in PKSs and condensation (C) domains in NRPSs. Additional domains can be present in the modules, such as ketoreductase (KR) and dehydratase (DH) domains in PKSs that control the oxidation state of the β-carbon, as well as methyltransferase and aminotransferase domains in PKSs and NRPSs that can further modify the natural product. Once synthesis of the natural product on a PKS or NRPS is complete, it is typically cleaved from the synthetase by a thioesterase (TE) domain. Further complexity can be introduced through hybrid systems that combine PKS and NRPS modules. These two types of modules can be encoded in the same protein, but they are often encoded in separate proteins. In this latter case, protein–protein interactions facilitate the direct passage of biosynthetic intermediates between the two proteins^16^.

Domain analysis of PKS-1 and NRPS-1 provides a number of tantalizing clues indicating that the biosynthesis of the nemamides deviates from a canonical pathway in several respects^8^. Both PKS-1 and NRPS-1 contain stretches of protein sequence with no homology to known enzymatic domains, and the enzymatic domains that can be identified are out of order and/or have diverged significantly from those found in other systems^8^. For example, the substrate preferences of the A domains cannot be predicted based on the presence of key residues present in their active sites^17^. Furthermore, there are no obvious domains that would enable certain structural features present in the nemamides, such as the *O*-methyl and amino groups, to be incorporated. Given that biosynthetic genes are not clustered in animals as they are in microorganisms, the identification of these missing domains in the worm represents a challenge. Additionally, while there is a TE domain at the C-terminus of NRPS-1, which presumably cleaves the natural product from the synthetase, there is also, strangely, a TE domain at the C-terminus of PKS-1 of unknown function.

Here, we map the biosynthesis of a complex metabolite in an animal system through genetic manipulation of the biosynthetic genes in vivo followed by comparative metabolomics. We show that the biosynthesis of the nemamides requires at least seven genes distributed across the worm genome that are united by their common expression in the CANs (Fig. 1a, b). Furthermore, we uncover the biosynthetic roles of these genes and show that the polyketide-ACP ligase PKAL-1 acts in trans and is required for the trafficking of intermediates between PKS-1 and NRPS-1. PKAL-1 represents a unique enzyme in that it loads a complex polyketide intermediate onto an NRPS for further chemical elaboration.

## Results

### NRPS-1 is responsible for incorporating all of the amino acid components of the nemamides

Assuming a linear, assembly-line mechanism in nemamide biosynthesis, we predicted that the β-Ala moiety in the nemamides is installed by the C-terminal NRPS module of PKS-1 and that the two D-Asn and final L-Asn moieties are installed by the NRPS modules of NRPS-1. However, the lack of sequence homology of the A domains in PKS-1 and NRPS-1 to bacterial and fungal A domains precluded predictions of the amino acid substrate specificities of these domains that might have supported our model^8,17^. Furthermore, our inability to express in *Escherichia coli* any of the PKS-1 or NRPS-1 A domains (either as excised domains or as part of multi-domain constructs) prevented us from analyzing the substrate preferences of the A domains in vitro. Therefore, we decided to inactivate specific domains in PKS-1 and NRPS-1 in the worm in order to map their biosynthetic roles. First, we used CRISPR–Cas9 to inactivate the TE domain in NRPS-1 by replacing the catalytic serine with alanine to generate strain *nrps-1[TE*_*2*_*_S2803A]* (Supplementary Fig. 1). This worm strain does not make the nemamides, consistent with the role of the NRPS-1 TE domain in cleaving nemamide from the synthetase through the formation of a macrolactam (Supplementary Fig. 2).

**Fig. 2:**
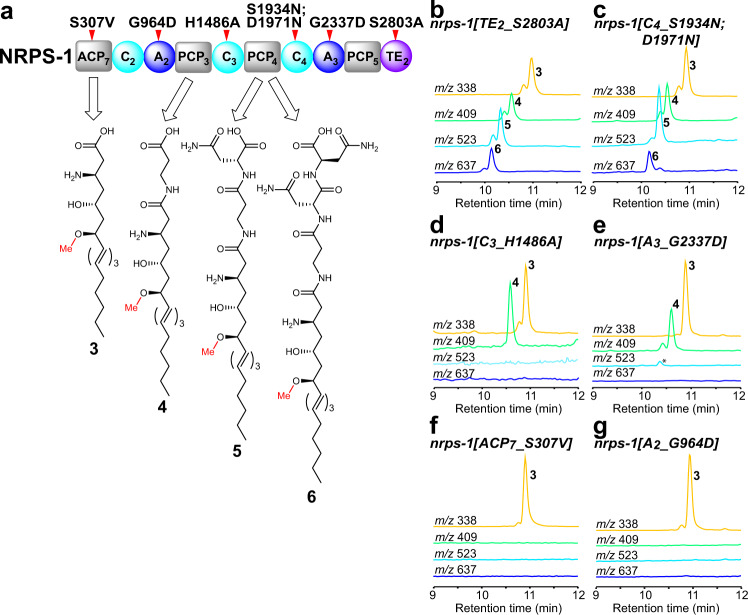
Analysis of biosynthetic intermediates in mutant strains in which specific NRPS-1 domains have been inactivated. **a** The structures of the intermediates that were identified in the NRPS-1 mutant strains and the proposed carrier proteins that carry them as the corresponding thioesters. Domain abbreviations: acyl carrier protein (ACP), peptidyl carrier protein (PCP), adenylation (A), condensation (C), thioesterase (TE). The domains that were mutated are indicated with a red tick mark. The chemical structures of the intermediates were confirmed through high resolution LC–MS/MS (Supplementary Figs. 4–7). **b**–**g** Representative extracted ion chromatograms of intermediates **3** ([M-H]^−^ *m/z* 338), **4** ([M-H]^−^ *m/z* 409), **5** ([M-H]^−^ *m/z* 523), and **6** ([M-H]^−^ *m/z* 637) in the NRPS-1 TE_2_ domain mutant *nrps-1(reb12[TE*_*2*_*_S2803A])* (**b**), the NRPS-1 C_4_ domain mutant, *nrps-1(gk186409[C*_*4*_*_S1934N];gk186410[C*_*4_*_*D1971N])* (**c**), the NRPS-1 C_3_ domain mutant, *nrps-1(reb10[C*_*3*_*_H1486A])* (**d**), the NRPS-1 A_3_ domain mutant, *nrps-1(reb31[A*_*3*_*_G2337D])* (**e**), the NRPS-1 ACP_7_ domain mutant, *nrps-1(reb8[ACP*_*7*_*_S307V])* (**f**), and the NRPS-1 A_2_ domain mutant, *nrps-1(reb32[A*_*2*_*_G964D])* (**g**). Note that the NRPS-1 C_4_ domain mutant contains a mutation near the active site (S1934N), as well as one more distal to the active site (D1971N). In (**e**), the asterisk (*) indicates that a very small amount of **5** is seen in this mutant (see Supplementary Fig. 3 for quantitative analysis of intermediates), likely because the mutation in the *nrps-1(reb31[A*_*3*_*_G2337D])* strain does not appear to completely block domain function as this strain can make a small amount of nemamides (see Supplementary Fig. 2).

Unexpectedly, the *nrps-1[TE*_*2*_*_S2803A]* strain accumulates a number of intermediates in the biosynthesis of the nemamides, one with no amino acids incorporated (**3**), one with β-Ala incorporated (**4**), one with β-Ala-D-Asn incorporated (**5**), and one with β-Ala-D-Asn-D-Asn incorporated (**6**) (Fig. 2a, b; Supplementary Fig. 3). Given that the nemamides are produced in very low amounts in *C. elegans*, and given that the biosynthetic intermediates are produced at even lower amounts, the intermediates had to be partially purified to enable identification. Totally, 2–3 L of worms grown in high-density axenic culture enabled the production of 3–5 g of worms, which were used to generate extracts that were then purified through two chromatographic steps. Intermediates were followed based on the characteristic ultraviolet (UV) spectrum of the triene or tetraene moiety that is present in them. Fortunately, this UV signature appears to be quite unique to nemamide and nemamide intermediates in *C. elegans* extracts. We verified the accumulation of intermediates **3**–**6** in the NRPS-1 TE domain mutant strain using high-resolution liquid chromatography–mass spectrometry (LC–MS)/MS (Supplementary Figs. 4–7). The amounts of the intermediates detected in this mutant were less than 10% of the mean amount of nemamide A in wild-type worms (Supplementary Fig. 3). The fact that the NRPS-1 TE domain mutant strain does not accumulate the linear form of nemamide A with the last L-Asn incorporated, suggests that this intermediate likely remains covalently attached to the NRPS-1 terminal PCP (PCP_5_) in the mutant.

We hypothesized that we might be able to map the biosynthesis of the nemamides by NRPS-1 by targeting individual domains in NRPS-1 and analyzing the intermediates that accumulate. We began with domains acting at the end of the nemamide biosynthetic pathway and worked our way backward. The *nrps-1[C*_*4*_*_S1934N;D1971N]* strain, in which an active site Ser of the NRPS-1 C_4_ domain was mutated, does not make the nemamides and accumulates intermediates **3**–**6** (Fig. 2a, c; Supplementary Figs. 1–3). This result suggests that the C_4_ domain in NRPS-1 incorporates the l-Asn into the growing chain before cyclization by the NRPS-1 TE domain to form the nemamides. The *nrps-1[C*_*3*_*_H1486A]* strain, in which the catalytic histidine of the NRPS-1 C_3_ domain was mutated, does not make the nemamides and accumulates intermediates **3** and **4** (Fig. 2a, d; Supplementary Figs. 1–3). The *nrps-1[A*_*3*_*_G2337D]* strain, in which a key glycine in the NRPS-1 A_3_ domain was mutated, also accumulates predominantly intermediates **3** and **4** (Fig. 2a, e; Supplementary Figs. 1–3). These results suggest that the C_3_ and A_3_ domains incorporate the two d-Asn moieties into the nemamides. Certain C domains catalyze both the epimerization and condensation of the amino acids that they incorporate, but none of the C domains in NRPS-1 have the characteristic features of these domains (Supplementary Fig. 8)^18^. Thus, it is unclear whether incorporation of the d-Asn moieties into the nemamides involves the A_3_ domain loading D-Asn or the A_3_ domain loading l-Asn, followed by epimerization to d-Asn by an unidentified epimerase.

The N-terminus of NRPS-1 contains a stretch of an unannotated sequence of 400 amino acids that has no obvious homology to PKS or NRPS domains, based on antiSMASH or BLAST analysis^19^. Using the modeling program SWISS-model, however, we were able to identify an ACP domain between residues 259 and 361 of this sequence (annotated as ACP_7_)^20^. The *nrps-1[ACP*_*7*_*_S307V]* strain, in which the predicted site of phosphopantetheinylation in ACP_7_ is mutated, does not make the nemamides, demonstrating that this ACP domain is functionally relevant (Supplementary Figs. 1 and 2). Surprisingly, this strain specifically accumulates intermediate **3**, but not **4**, revealing that the β-alanine residue in the nemamides is not incorporated by the C-terminal NRPS module of PKS-1, as we had originally proposed, but by the N-terminal NRPS module of NRPS-1 (Fig. 2a, f; Supplementary Fig. 3). In further support of this model, the *nrps-1[A*_*2*_*_G964D]* strain, in which a key glycine in the NRPS-1 A_2_ domain is mutated, accumulates intermediate **3**, but not **4**, indicating that A_2_ loads β-alanine onto NRPS-1 for incorporation into the nemamides (Fig. 2a, g; Supplementary Figs. 1–3). Since disruption of the NRPS-1 ACP_7_, A_2_, A_3_, C_3_, C_4_, and TE_2_ domains leads to the accumulation of intermediate **3**, this intermediate is most likely generated by PKS-1 and transferred onto NRPS-1 ACP_7_ for further elongation. Furthermore, because intermediate **3** contains the amino and *O*-methyl groups, incorporation of these groups must precede the biosynthetic steps carried out by NRPS-1.

### Role of the C-terminal NRPS module and TE domain in PKS-1

**Fig. 3:**
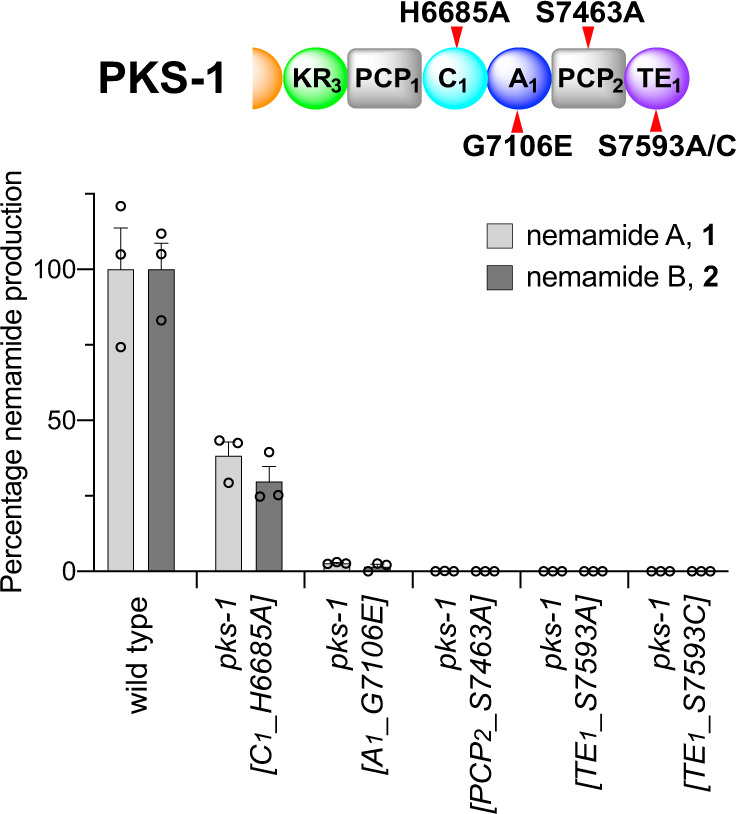
Analysis of nemamide production in mutant strains in which different domains in the C-terminal NRPS module of PKS-1 have been inactivated. The C-terminal NRPS module was inactivated by mutating essential residues in each domain. In the C_1_ domain, the catalytic residue His 6685 was mutated to Ala. In the A_1_ domain, Gly 7106, which is essential for cofactor binding, was mutated to Glu. In the PCP_2_ domain, the site of phosphopantheinylation, Ser 7463, was mutated to Ala. In the TE_1_ domain, Ser 7593, which is part of the catalytic triad, was mutated to either Ala or Cys. The domains that were mutated are indicated with a red tick mark. Domain abbreviations: ketoreductase (KR), peptidyl carrier protein (PCP), adenylation (A), condensation (C), thioesterase (TE). The amount of nemamide A or B in each strain relative to the amount of nemamide A or B in wild-type worms was based on the UV absorbance of the compounds and was normalized by the total mass of dried worms used for extraction. Data represent the mean ± SEM, *n* = 3 independent experiments in which worms obtained from a culture were extracted for nemamide analysis. Source data are provided as a Source Data file.

Given that the β-alanine moiety is incorporated by NRPS-1, it was unclear whether the C-terminal NRPS module in PKS-1 would be required for nemamide biosynthesis. The domain organization of this NRPS module in PKS-1, as well as the sequence of the A domain in this module, are highly conserved across nematode evolution, suggesting that the NRPS module of PKS-1 does play an important role in the biosynthesis (Supplementary Table 1)^8^. Three mutant worm strains containing mutations in either the A_1_, C_1_, or PCP_2_ domains of the NRPS module in PKS-1 were generated (Fig. 3; Supplementary Fig. 1). While the C_1_ domain mutant could still produce some nemamides (less than 40%), the A_1_ domain mutant made only very minor amounts of nemamides and the PCP_2_ domain mutant did not make any at all (Fig. 3; Supplementary Fig. 9). Thus, although the C-terminal NRPS module of PKS-1 is not involved in incorporating β-alanine, it is required for nemamide biosynthesis. None of these strains accumulated any intermediates with UV signatures characteristic of trienes or tetraenes, suggesting that if any such intermediates do accumulate, they remain covalently linked to the synthetase.

The NRPS module in PKS-1 is followed by a C-terminal TE domain of unknown function. Comparison of this TE domain to bacterial TEI domains, which are involved in the cleaving of final products from the synthetase, and bacterial TEII domains, which facilitate synthesis by cleaving misloaded intermediates from carrier proteins, suggests that the PKS-1 TE domain is most similar to bacterial TEI domains found in hybrid PKS-NRPS systems^8,21,22^. We generated two mutant worm strains, *pks-1[TE*_*1*_*_S7593A]* and *pks-1[TE*_*1*_*_S7593C]*, in which the catalytic serine residue of the PKS-1 TE domain was mutated to either an alanine or a cysteine, and showed that these strains do not make any nemamides or accumulate any biosynthetic intermediates (Fig. 3; Supplementary Fig. 1; Supplementary Fig. 9). This result suggests that the PKS-1 TE domain is not functioning in an editing fashion as a TEII domain, since such an editing enzyme would typically not be essential for biosynthesis, but only facilitate it^22^. Instead, this result suggests that the PKS-1 TE domain is playing an essential role, most likely in the trafficking of intermediates between PKS-1 and NRPS-1.

### Identification of *trans*-acting enzymes involved in nemamide biosynthesis

**Fig. 4:**
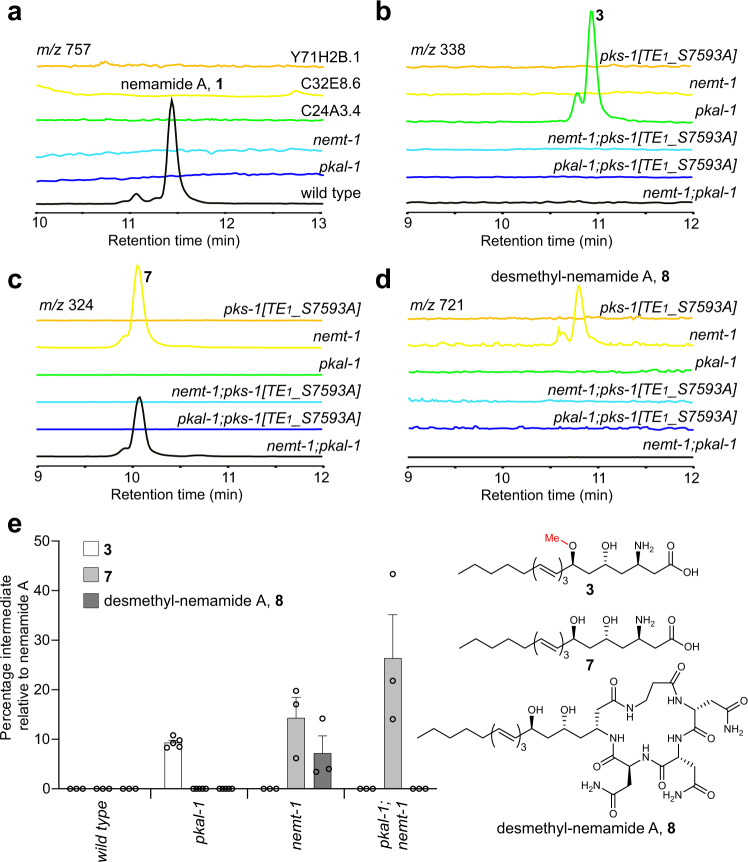
Analysis of nemamides and biosynthetic intermediates in mutant strains in which *trans*-acting enzymes have been inactivated. **a**–**d** Representative extracted ion chromatograms of nemamide A, **1**, (**a**) in mutant strains for the *trans*-acting enzymes and intermediate **3** (**b**), intermediate **7** (**c**), and desmethyl-nemamide **8** (**d**) in the *pks-1(reb11[TE*_*1*_*_S7593A])*, *pkal-1(reb28)*, *nemt-1(reb15)*, and double mutant strains. **e** The amounts of intermediates **3**, **7**, and **8** in mutant strains relative to the amount of nemamide A in wild-type worms were based on the UV absorbance of the compounds and were normalized by the total mass of dried worms used for extraction. Data in (**e**) represent the mean ± SEM, *n* = 5 independent experiments for *pkal-1* mutant and *n* = 3 independent experiments for wild type and other mutants. Source data are provided as a Source Data file.

We hypothesized that certain steps in nemamide biosynthesis would require additional enzymes beyond PKS-1 and NRPS-1 and that these enzymes would likely also be expressed in the CANs. Single-cell gene expression profiling has been performed in multiple cell types in *C. elegans*, including the CANs^23,24^. 38 genes, including *pks-1* and *nrps-1*, show selective expression in the CANs that is at least fivefold higher than in the next tissue in which they are expressed (Supplementary Table 2)^23^. Based on their homology and predicted enzymatic activities, we screened a number of these genes by analyzing nemamide production in loss-of-function mutant worm strains. These data show that at least five additional genes are required for nemamide biosynthesis, F49C12.10, T20F7.7, C32E8.6, C24A3.4, and Y71H2B.1 (Fig. 4a; Supplementary Fig. 10). F49C12.10, which we named NEMT-1 (NEmamide *O*-MethylTransferase-1), is predicted to be an *O*-methyltransferase since its closest non-nematode homolog is phthiotriol methyltransferase (with 31% identity), which methylates a precursor to cell surface-associated apolar lipids in mycobacteria^25,26^. T20F7.7 and C32E8.6 are homologous to acyl-CoA synthetases, but the failure of the T20F7.7 and C32E8.6 genes to inter-rescue suggests that they play distinct roles in nemamide biosynthesis (Supplementary Fig. 11). T20F7.7 was previously named ACS-9 due to its homology to acyl-CoA synthetases. However, we have renamed this enzyme PKAL-1 (PolyKetide-ACP Ligase-1) for reasons described below, namely that it does not have acyl-CoA synthetase activity, but instead loads a polyketide intermediate in nemamide biosynthesis directly onto an ACP domain. Lastly, C24A3.4 is annotated as an α-methylacyl-CoA racemase or CoA transferase, and Y71H2B.1 is annotated as a fatty acyl-CoA binding protein. For all five genes, nemamide production could be rescued in loss-of-function strains by complementing the genes by expressing them under the control of their own promoter (Supplementary Fig. 12). By generating translational reporter strains, we were able to verify that all of the additional genes required for nemamide biosynthesis are primarily expressed in the CANs, although C24A3.4 is also expressed in the intestine (Supplementary Fig. 13). Interestingly, unlike natural product biosynthetic genes in bacteria and fungi, which are often clustered, the nemamide biosynthetic genes are scattered across the *C. elegans* genome with C32E8.6 on chromosome I, *nrps-1* and Y71H2B.1 on different ends of chromosome III, *nemt-1* on chromosome IV, and *pks-1*, *pkal-1*, and C24A3.4 spread across the X chromosome (Fig. 1b).

### Trafficking of intermediates between PKS-1 and NRPS-1

To investigate the role of the *trans*-acting enzymes in nemamide biosynthesis, we characterized the intermediates that accumulate in the corresponding mutant worm strains. The *pkal-1* mutant strain accumulates **3**, which is the same intermediate that accumulated in the strain with the ACP_7_ domain of NRPS-1 mutated (Fig. 2a, f; Fig. 4b, e). On the other hand, the *pkal-1; pks-1[TE*_*1*_*_S7593A]* double mutant strain does not accumulate **3**, indicating that PKAL-1 functions downstream of the PKS-1 TE_1_ domain (Fig. 4b,e). We hypothesized that PKAL-1 might be involved in loading **3** onto the ACP_7_ domain of NRPS-1 for further extension.

The *nemt-1* mutant strain accumulates intermediate **7**, which is similar to **3**, but lacks the methyl group (Fig. 4c, e; Supplementary Fig. 14). This result indicates that NEMT-1 is the *O*-methyltransferase that is responsible for the methoxy group that is present in the nemamides. The *nemt-1* mutant strain also produces **8**, which is similar to nemamide A, but lacks the methyl group (Fig. 4d, e; Supplementary Fig. 15). The fact that the *nemt-1* mutant can make desmethyl-nemamide **8**, suggests that PKAL-1 can load **7** onto the ACP_7_ domain of NRPS-1, even though it is lacking the methyl group. However, because the *nemt-1* mutant accumulates intermediate **7**, PKAL-1 and/or NRPS-1 likely prefers methylated substrates over unmethylated ones (Fig. 4e). Unlike the *nemt-1* single mutant, the *nemt-1; pks-1[TE*_*1*_*_S7593A]* double mutant does not accumulate intermediate **7** or desmethyl-nemamide **8**, suggesting a possible model in which NEMT-1 functions downstream of the PKS-1 TE_1_ domain. Thus, our genetic data suggest that NEMT-1 methylates intermediate **7** to form **3**, and then PKAL-1 is involved in loading **3** onto the ACP_7_ domain of NRPS-1 for further extension.

No biosynthetic intermediates were found in Y71H2B.1, C24A3.4, or C32E8.6 loss-of-function mutant worm strains. Given that intermediates also do not accumulate in *pks-1* mutant worm strains, these three genes may function in earlier stages of nemamide biosynthesis, potentially either in the initiation or in other steps involving PKS-1.

### Biochemical activity of PKAL-1

**Fig. 5:**
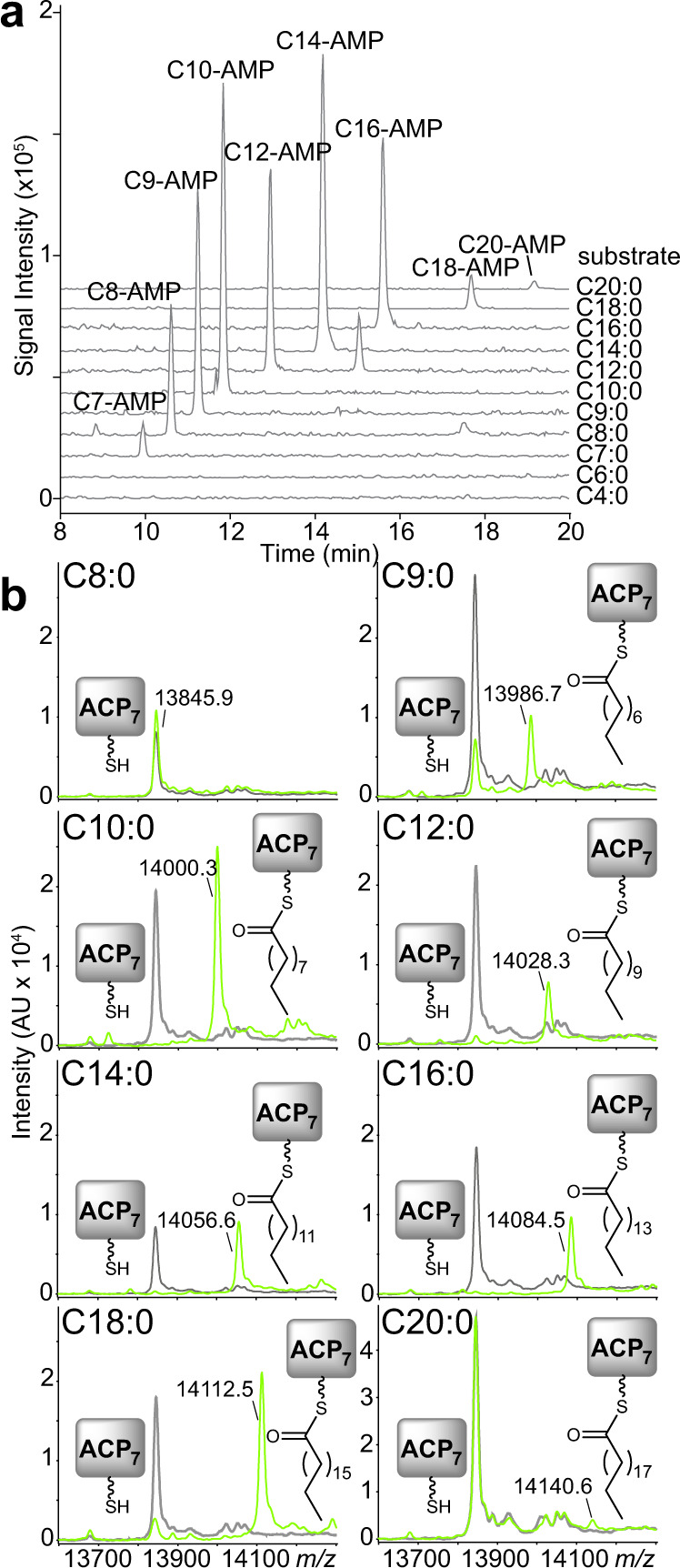
In vitro activity of PKAL-1 against fatty acid substrates. **a** Activity of PKAL-1 towards fatty acids of various lengths in the presence of ATP and CoA. Extracted ion chromatograms are representative of at least three independent experiments. The identities of the peaks were verified based on their masses in both positive and negative modes, as well as their UV spectra. The catalytic mutant PKAL-1(K488A) did not show any product formation. The inability of PKAL-1 to form CoA-thioesters was verified with synthetic standards (Supplementary Fig. 16). **b** MALDI-TOF analysis of the holo-ACP_7_ domain from NRPS-1 incubated with PKAL-1 (light green) or PKAL-1(K488A) (gray), fatty acids of various lengths, and ATP. MALDI spectra are representative of at least three independent experiments.

Based on its sequence homology, we hypothesized that PKAL-1 might function similarly to a fatty acyl-CoA ligase (FACL). That is, PKAL-1 might activate **3** as the adenylate and then react that intermediate with CoA to form a CoA-thioester that is subsequently loaded onto the ACP_7_ domain of NRPS-1. To biochemically characterize the role of PKAL-1 in trafficking intermediates between PKS-1 and NRPS-1, we cloned the enzyme from a cDNA library, expressed it in *E. coli*, and purified it for biochemical characterization. We then incubated PKAL-1 with fatty acids of various lengths, ATP, and CoA and analyzed the products by LC–MS. PKAL-1 can activate a variety of medium and long-chain fatty acids as the corresponding fatty acyl-AMP but cannot further convert them to the corresponding fatty acyl-CoA (Fig. 5a, Supplementary Fig. 16). A negative control, PKAL-1(K488A), in which a lysine predicted to be important for catalysis was mutated^27^, showed no activity. These results suggest that PKAL-1 is analogous to a fatty acyl-AMP ligase (FAAL) rather than a FACL. FAAL enzymes activate fatty acids as the corresponding fatty acyl-AMP but then transfer the fatty acyl group to the phosphopantetheinyl arm of a carrier protein instead of CoA^28–30^. FAAL enzymes typically have an insertion motif that prevents the movement between the larger N-terminal domain and the smaller C-terminal domain that occurs between the adenylation and CoA-ligase reactions^28,29^. Certain FAAL enzymes lack the insertion motif but instead have additional interactions between the N-terminal and C-terminal domains that are thought to prevent the CoA-ligase reaction from occurring^31^. Although sequence alignment of PKAL-1 demonstrates that it is missing the insertion motif (Supplementary Fig. 17), structural modeling of PKAL-1 indicates that it might lack CoA ligase activity due to the absence of an effective binding site for CoA (Supplementary Fig. 18).

FAAL enzymes play an important role in the biosynthesis of lipid-modified polyketides and nonribosomal peptides, such as mycolic acids that are essential for mycobacterial growth, isonitrile lipopeptides that promote mycobacterial virulence, and diverse lipopeptides that are widespread in cyanobacteria^28–33^. In order to determine whether PKAL-1 can load fatty acids that are similar in length to intermediate **3** onto the ACP_7_ domain of NRPS-1, the excised ACP_7_ domain was first co-expressed in *E. coli* with the promiscuous phosphopantetheinyl transferase Sfp^34^, enabling an efficient purification of the holo-ACP_7_ domain. This holo-ACP_7_ domain was incubated with PKAL-1, ATP, and fatty acids of various lengths, and the products were analyzed by matrix-assisted laser desorption ionization time-of-flight (MALDI-TOF). PKAL-1 could efficiently load fatty acids that were 9–18 carbons in length and could load a minor amount of C20 fatty acids. (Fig. 5b). This result is consistent with a role for PKAL-1 in activating **3**, which is 18 carbons in length. PKAL-1 was unable to load fatty acids onto a different ACP, ACP_1_ from PKS-1 (Supplementary Fig. 19). Thus, PKAL-1 must engage in specific interactions with the ACP_7_ domain of NRPS-1 in order to load fatty acids onto it. To characterize further PKAL-1’s substrate preferences, we utilized an enzyme-coupled continuous kinetic assay in which hydroxylamine was used instead of an ACP as the substrate acceptor^35^. Importantly, PKAL-1 is much more active towards long-chain fatty acids than short- and medium-chain fatty acids, with C16 being preferred over C14, which is in turn preferred over C12, which is in turn preferred over C10 and C8 (Supplementary Fig. 20). Fatty acids longer than C16 could not be tested due to solubility issues and/or detergent effects. The kinetic assay thus demonstrates that the substrate preferences of PKAL-1 are consistent with its proposed role in activating and loading **3**. While FAAL enzymes have been shown to activate fatty acids to initiate polyketide and nonribosomal peptide biosynthesis^27–29,33,36^, PKAL-1 is unusual in that it activates an advanced polyketide intermediate for loading onto an NRPS for further elaboration.

## Discussion

Using comparative metabolomics, we have mapped the biosynthetic pathway to the nemamides, a remarkable family of hybrid polyketide-nonribosomal peptides biosynthesized in the CANs of *C. elegans*. Furthermore, we have determined the biosynthetic roles of five additional enzymes from *C. elegans* that function in trans in nemamide biosynthesis. Although the different genes that are required for nemamide biosynthesis are encoded in disparate locations across the worm genome, these genes share a common feature in that they are expressed in the CANs. Our work has shown that at least 7 of the 38 genes that have enriched expression in the CANs^23^ are involved in nemamide biosynthesis, suggesting that nemamide biosynthesis may be a primary function of these enigmatic neurons.

**Fig. 6:**
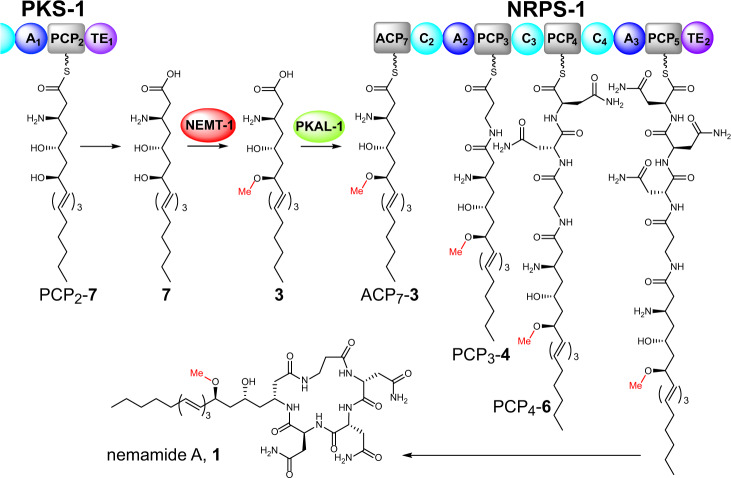
Proposed model for the biosynthesis of the nemamides. The methylated intermediate **3** is activated by PKAL-1 as the adenylate and loaded onto the ACP_7_ domain of NRPS-1 in order to biosynthesize nemamide A, **1**. An analogous pathway biosynthesizes nemamide B, **2**. Our data suggest that PKS-1 produces and releases **7**, which is then methylated by NEMT-1 to generate **3**. However, it is also possible that NEMT-1 acts earlier in the pathway on a polyketide intermediate attached to PKS-1, such that PKS-1 produces and releases **3**, not **7** (see “Discussion”). Domain abbreviations: acyl carrier protein (ACP), peptidyl carrier protein (PCP), adenylation (A), condensation (C), and thioesterase (TE).

Our data show that all of the amino acids in the nemamides, including the β-alanine, are incorporated by NRPS-1 and that the C-terminal NRPS module of PKS-1 has other functions in nemamide biosynthesis (Fig. 6). Mutation of the PCP_2_ domain of PKS-1 completely blocks nemamide production, and mutation of the A_1_ domain of PKS-1 enables only trace amounts of nemamides to be produced; thus, the NRPS module of PKS-1 does play a role in nemamide biosynthesis. Furthermore, both the domain organization of this NRPS module in PKS-1 and the sequence of the A domain have been conserved across nematode species^8^. As we have not be able to identify an aminotransferase for incorporation of the amino group into the nemamides, this module may be involved in this process. A possible mechanism for the incorporation of an amino group into the nemamides is suggested by the biosynthesis of the β-amino fatty acid starter unit in the macrolactam family of antibiotics that includes BE-14106 and ML-449^37–40^. The biosynthesis of the β-amino fatty acid is thought to require a free-standing A domain, a free-standing PCP domain, and a free-standing glycine oxidase to incorporate the amino group from glycine into an α-β unsaturated fatty acyl precursor^37,38^. It should be noted that a C domain is not involved in this proposed mechanism, potentially explaining why the C_1_ domain of PKS-1 is not required for nemamide biosynthesis. Importantly, however, we have not yet identified a candidate glycine oxidase in the worm genome, and thus, it is unclear whether the amino group in the nemamides is incorporated in an analogous fashion as the amino group is incorporated in BE-14106 and ML-449.

Trafficking between PKS and NRPS enzymes typically involves protein–protein interactions between the megasynthetases that enable direct passage of intermediates from the carrier domain on an upstream PKS or NRPS to the KS or C domain on the downstream PKS or NRPS, respectively^16^. Here, we have shown that trafficking of intermediates between PKS-1 and NRPS-1 involves production of a complex polyketide intermediate by PKS-1 and NEMT-1 (i.e., **3**) that is activated and loaded by PKAL-1 onto the N-terminal ACP domain of NRPS-1 for subsequent production of the nemamides (Fig. 6). Specifically, we have shown that mutant worms containing mutations in either PKAL-1 or the ACP domain of NRPS-1 accumulate **3**. Furthermore, through biochemical experiments, we have demonstrated that PKAL-1 activates and loads fatty acid substrates of similar length to **3** onto the ACP domain of NRPS-1, but not onto other ACP domains. Since an unmethylated version of **3** (i.e., **7**) accumulates in the *nemt-1* mutant strain, it is likely that PKS-1 produces **7**, which is then methylated by NEMT-1 to make **3** (Fig. 6). It remains possible, however, that NEMT-1 acts earlier in the biosynthetic pathway on an intermediate that is attached to the PKS-1 assembly line. However, for this alternative model to be correct, we must assume that in the *nemt-1* mutant, the PKS-1 assembly line can continue to process unmethylated intermediates as well as it can the methylated ones that are present in wild-type worms. Furthermore, we must assume that in the *nemt-1* mutant the unmethylated polyketide product **7** accumulates simply due to it being a non-preferred substrate for PKAL-1. This model seems less likely given that the *nemt-1* strain is able to produce desmethyl-nemamide A (**8**), indicating that PKAL-1 (and NRPS-1) can process desmethyl intermediates. It is unclear why only small amounts of **7** and **3** accumulate in the *nemt-1* and *pkal-1* mutants, respectively, relative to the amount of nemamides in wild type worms. This result could indicate that these biosynthetic intermediates are less stable than the nemamides, or it could indicate that there is some type of feedback mechanism that prevents these intermediates from accumulating. The mechanism of trafficking intermediates between PKS-1 and NRPS-1 may be important for the movement of intermediates in nemamide biosynthesis across membranes or between cellular compartments.

The PKAL-1 enzyme is unique in that it activates an advanced polyketide intermediate as the adenylate and loads it onto the ACP of the next megasynthetase in the assembly line, NRPS-1. There are many examples of FAAL enzymes activating fatty acids as AMP-esters for loading onto a carrier protein for an extension by either a PKS or NRPS^28,29,31^. However, to our knowledge, PKAL-1 is unique in that it activates an advanced polyketide substrate and loads it onto a carrier protein for further assembly-line processing on an NRPS. Furthermore, PKAL-1 is unusual in that it does not contain the insertion motif that characterizes most FAAL enzymes^28–33^. Thus, other structural features, such as an inability to bind CoA, must explain its failure to act as a CoA ligase. Interestingly, PKAL-1 is upregulated under starvation conditions in a manner that is independent of the transcription factor DAF-16, which promotes survival during starvation-induced larval arrest^41,42^. Previously, we showed that the nemamides promote survival during starvation-induced larval arrest in a manner that is at least partially DAF-16-independent and that bacterial food suppresses the production of the nemamides^8^. Thus, the requirement of PKAL-1 for nemamide production may help to couple nemamide production to food availability.

In summary, we have shown that nemamide biosynthesis requires at least seven different genes distributed throughout the worm genome that are united by their common expression in the CANs. Furthermore, we have shown that the PKAL-1 enzyme is required for the trafficking of intermediates between PKS-1 and NRPS-1. Specifically, PKAL-1 loads an advanced polyketide intermediate produced by PKS-1 and NEMT-1 onto NRPS-1. This unique trafficking mechanism may facilitate the regulation of nemamide biosynthesis.

## Methods

### Worm strains

Worms were maintained on OP50 using standard methods. Strains used in this study were obtained from the Caenorhabditis Genetics Center or were generated through genome editing by CRISPR–Cas9 or through transgenesis (Supplementary Table 3). Double mutants were generated from single mutants, using standard genetic crossing methods. The presence of alleles was verified through single worm polymerase chain reaction (PCR) using the primers in Supplementary Table 4, and the PCR products were analyzed by restriction site digestion, as shown in Supplementary Table 5.

### Single worm PCR and CRISPR–Cas9

Most mutant strains containing deletions and point mutations were generated based on the Fire lab’s CRISPR–Cas9 protocol^43,44^. The concentration of the Cas9 vector used was 50 ng/µL. The plasmid for expressing the *dpy-10* sgRNA was used at 25 ng/µL, and the other plasmids for expressing the sgRNA sequences for target genes were used at 50–100 ng/µL (see Supplementary Table 6 for sequences). The *dpy-10(cn64)* donor oligonucleotide was used at a concentration of 500 nM, and other donor oligonucleotides for generating desired mutations were used at 500–750 nM (see Supplementary Table 7 for sequences). To generate deletion mutants, no donor oligonucleotide was used for either *dpy-10* or targeted genes. After microinjection, F1 worms with *dpy* (for deletions) and *roller* (for point mutations) phenotypes were picked for single worm PCR using the primers listed in Supplementary Table 4 and restriction digestion (only for mutants with point mutations) of the PCR products as shown in Supplementary Table 5. The PCR products of positive candidates were sequenced to confirm their alleles, and *dpy* worms were backcrossed with wild-type (N2) worms.

The *nrps-1(reb32[A*_*2*_*_G964D])* and *nrps-1(reb31[A*_*3*_*_G2337D])* strains were generated using the Mello lab’s CRISPR-Cas9 protocol^45^. All reagents to generate the Cas9 ribonucleoprotein (RNP) complex were ordered from IDT. Each injection mixture contained 25 ng/μL Cas9, 100 ng/μL tracrRNA, 56 ng/μL crRNA (see Supplementary Table 6 for sequences), 110 ng/μL of the ssDNA donor (see Supplementary Table 7 for sequences), and 40 ng/μL pRF4*(rol-6(su1006))*. The RNP complex was generated by incubating Cas9, tracrRNA, and crRNA for 15 min at 37 °C. The ssDNA donor and pRF4*(rol-6(su1006)* were combined with the RNP and centrifuged at 16,000 × *g* for 5 min. After microinjection, F1 worms exhibiting the roller phenotype were picked for single worm PCR using the primers listed in Supplementary Table 4 and subsequent restriction digestion of the PCR products as shown in Supplementary Table 5. The PCR products of positive candidates were sequenced to confirm their alleles.

### Transgenesis

Transcriptional reporter plasmids were made by amplifying the promoter from genomic DNA using the primers in Supplementary Table 8 and inserting the product into pPD114.108. Translational reporter plasmids were made by amplifying the promoter and gene from genomic DNA using the primers in Supplementary Table 8 and inserting the product into pBS77-SL2-mCherry. The reporter plasmids were injected into the corresponding mutant worm strains at 50 ng/µL. The *coel::dsred* plasmid was used as a co-injection marker at 25 ng/μL, and the total concentration of DNA injected was 100 ng/μL including pUC18. At least three independent transgenic lines were analyzed.

### Small-scale worm extraction for nemamide production

Wild-type and mutant worm strains were grown at room temperature on two NGM agar plates (10 cm) spread with 0.75 mL 25X OP50 until the food on the plates was almost gone. Then, the worms were transferred to a 1 L Erlenmeyer flask containing S medium (350 mL). The worm cultures were grown at 22.5 °C for 3–5 d until no food was left and were fed with 3.5 mL of 25X OP50 every day. For sample collection, the culture flasks were placed in an ice bath for 30 min to 1 h to settle the worms. Then, the worms were transferred from the bottom of the flasks to a 50 mL centrifuge tube and were centrifuged (220 × *g* for 5 min) to separate the worms from the culture medium. The process was repeated until most of the worms were removed from the flasks. The collected worms were washed with water three times and centrifuged (220 × *g* for 5 min), and then they were soaked in 10 mL of water for 1 h in a shaking incubator (22.5 °C, 225 rpm) to remove bacteria from their digestive tract. The worms were collected by centrifugation and freeze-dried. The dried worm pellets were ground with sea sand (2 g sand per 200 mg dried worms) using a mortar and pestle. The ground worms were extracted with 15 mL of 190 proof ethanol for 3.5 h, and the extract was centrifuged (2700 × *g* for 20 min). The supernatant was collected and dried using a speed vac. The dried worm samples were each resuspended in 100 μL of methanol, sonicated (if needed), and centrifuged (18,400 × *g* for 1 min) before analysis by LC–MS. The samples were analyzed using a Luna 5 μm C18 (2) column (100 × 4.6 mm; Phenomenex) coupled with an Agilent 6130 single quad mass spectrometer operating in single ion monitoring (SIM) mode for nemamide A ([M + Na]^+^ *m/z* 757) and nemamide B ([M + Na]^+^ *m/z* 755). The following solvent gradient was used with a flow rate of 0.7 mL/min: 95% buffer A, 5% buffer B, 0 min; 0% buffer A, 100% buffer B, 20 min; 0% buffer A, 100% buffer B, 22 min; 95% buffer A, 5% buffer B, 23 min; 95% buffer A, 5% buffer B, 26 min (buffer A, water with 0.1% formic acid; buffer B, acetonitrile with 0.1% formic acid). Two *pkal-1* mutants, RAB58 (*reb21* allele) and RAB59 (*reb28* allele) were analyzed on small scale, and neither of them produced any nemamides. Two C32E8.6 mutants, RAB60 (*reb23* allele) and RAB61 (*reb24* allele), were also analyzed, and neither produced any nemamides.

### Large-scale worm extraction for biosynthetic intermediates

Egg prep of wild-type or a mutant strain was performed in order to inoculate 5 mL of CeHR medium, which was grown for 7–10 d. This 5 mL culture was then used to inoculate 50 mL of CeHR medium, which was grown for 7–10 d. This 50 mL culture was then used to inoculate 500 mL of CeHR medium in a 2.8 L baffled flask, and the culture was grown for 7–10 d. CeHR medium contained 20% cow’s milk^46^, and cultures were grown at 180 rpm at 20 °C. Worms were collected by centrifugation, washed with water, shaken in water for 30 min to clear their intestines, and washed again with water. Worms were stored frozen at −20 °C until needed. For the extraction and fractionation process, worms from 2 to 3 l-worth of culture (around 3–5 g of dried worms) were processed at a time. After freeze-drying, worms were ground for 15 min with 30–50 g of sand using a mortar and pestle. The pulverized worms were transferred to a 1 L Erlenmeyer flask, and 300 mL of 190-proof ethanol was added to the flask. The flask was shaken at 300 rpm for 3.5 h. The extract was filtered using a Buchner funnel and filter paper and evaporated with a rotavap at 25 °C. The extract was then subjected to silica gel chromatography (50–100 g silica gel) and eluted with a gradient of hexane, ethyl acetate, ethyl acetate/methanol (1:1), and methanol (350 mL each) to give four fractions (A–D). Fractions C and D were processed separately, evaporated with a rotavap at 27 °C, redissolved in 12 mL of methanol, and centrifuged at 2700 × *g* for 10 min. The supernatant was dried and dissolved in 10 mL of 70% methanol/water. The resulting cloudy sample was then applied to an HP-20ss column (100 g HP-20ss resin), eluting with methanol/water (7:3 to 9:1 to 10:0) to give twelve subfractions (1–12, 125 mL each). Each fraction was dried by rotavap and analyzed by LC–MS for peaks with the same characteristic triene/tetraene UV spectrum found in the nemamides. The amount of nemamide or intermediate was calculated using ChemStation (Agilent), based on the total UV area of the LC traces (UV at 280 nm for nemamide A-like molecules and UV at 320 nm for nemamide B-like molecules), normalized by sample dilutions and the total amount (g) of dried worms used for extraction. UV peaks were verified by masses in both positive and negative modes. The percentage for each compound in each sample was compared to the mean amount of nemamide A in wild type. *pkal-1* or C32E8.6 mutants containing different alleles were analyzed, and they showed very similar intermediate patterns. For the *pkal-1* mutant, the *reb28* allele was used for most analyses of single and double mutants. For the C32E8.6 mutant, the *reb23* allele was used for most analyses of single mutants. The extracts were analyzed using a Luna 5 μm C18 (2) column (100 × 4.6 mm; Phenomenex) coupled with an Agilent 6130 single quad mass spectrometer operating in both positive and negative mode under full scan and SIM modes. The following solvent gradient was used with a flow rate of 0.7 mL/min: 95% buffer A, 5% buffer B, 0 min; 0% buffer A, 100% buffer B, 20 min; 0% buffer A, 100% buffer B, 22 min; 95% buffer A, 5% buffer B, 23 min; 95% buffer A, 5% buffer B, 26 min (buffer A, water with 0.1% formic acid; buffer B, acetonitrile with 0.1% formic acid). Retention times were normalized based on the retention time of nemamide A. Intermediate **3**, ESI (*m/z*): [M+H]^+^ = 340, [M + H-CH_3_OH]^+^ 308, [M − H]^−^ 338; Intermediate **4**, ESI (*m/z*): [M + H]^+^ 411, [M + H-CH_3_OH]^+^ 379, [M − H]^−^ 409; Intermediate **5**, ESI (*m/z*): [M + H]^+^ 525, [M − H]^−^ 523; Intermediate **6**, ESI (*m/z*): [M + H]^+^ 639, [M − H]^−^ 637; Intermediate **7**, ESI (*m/z*): [M + H]^+^ 326, [M + H-H_2_O]^+^ 308, [M − H]^−^ 324; desmethyl-nemamide A, **8**, ESI (*m/z*): [M + Na]^+^ 743, [M + H]^+^ 721, [M + H-H_2_O]^+^ 703, [M − H]^−^ 719.

### Identification of nemamide biosynthetic intermediates by qTOF

Extracted nemamide biosynthetic intermediates were analyzed by HR–LC–MS and HR–LC–MS/MS in positive ion mode. A 5 μL of the sample was analyzed on a Capillary LCMS Solutions 3 μm 200 Å (0.3 × 150 mm) ProtoSIL C18AQ + column attached to an UltiMate 3000 capillary RSLC System and a Bruker Impact II QTOF mass spectrometer equipped with an Apollo II ion funnel ESI source with the following settings: mass range of *m/z* 80–1300 at a rate of 1 Hz, the capillary voltage at 2.5 kV, source temperature at 200 °C, drying gas at 4.0 L/min, and nebulizer gas at 0.3 bar. Mobile phase A was water with 10 mM ammonium formate and 0.1% formic acid, and mobile phase B was acetonitrile with 0.1% formic acid. The following solvent gradient was used for separation with a flow rate of 5 μL/min: 98% buffer A, 2% buffer B, 0 min; 98% buffer A, 2% buffer B, 5 min; 40% buffer A, 60% buffer B, 35 min; 5% buffer A, 95% buffer B, 55 min; 5% buffer A, 95% buffer B, 67 min; 98% buffer A, 2% buffer B, 70 min; 98% buffer A, 2% buffer B, 75 min. HR–LC–MS/MS was performed for each sample with a collision energy of 30 or 15 eV. Data were analyzed using Compass DataAnalysis software (Bruker). Intermediate **3**, HR-ESIMS (*m/z*): [M + H]^+^ calcd. for C_19_H_34_NO_4_ 340.2482, found 340.2483; intermediate **4**, HR-ESIMS (*m/z*): [M + H]^+^ calcd. for C_22_H_39_N_2_O_5_ 411.2853, found 411.2849; intermediate **5**, HR-ESIMS (*m/z*): [M + H]^+^ calcd. for C_26_H_45_N_4_O_7_ 525.3283, found 525.3281; intermediate **6**, HR-ESIMS (*m/z*): [M + H]^+^ calcd. for C_30_H_51_N_6_O_9_ 639.3712, found 639.3716; intermediate **7**, HR-ESIMS (*m/z*): [M + H]^+^ calcd. for C_18_H_32_NO_4_ 326.2326, found 326.2326; desmethyl-nemamide A, **8**, HR-ESIMS (*m/z*): [M + Na]^+^ calcd. for C_33_H_52_N_8_O_10_Na 743.3699, found 743.3694, [M + H]^+^ calcd. for C_33_H_53_N_8_O_10_ 721.3879, found 721.3879, [M-H_2_O + H]^+^ calcd. for C_33_H_51_N_8_O_9_ 703.3774, found 703.3769.

### Plasmid construction, protein overexpression, and purification

All genes and excised domains were amplified by PCR using Phusion polymerase (New England Biolabs) from a *C. elegans* cDNA library. Specifically, *pkal-1*, *pks-1_ACP*_*1*_, and *nrps-1_ACP*_*7*_ were amplified with specific primers (Supplementary Table 9), and the PCR products were inserted using *Nco*I and *Not*I into the pET16b-KH01 vector (a modified version of pET-16b)^47^ such that the expressed proteins were expressed with C-terminal His tag. All of the sequences were verified by sequencing. The PKAL-1(K488A) mutation was generated via Q5 site-directed mutagenesis kit (New England Biolabs) using a specific primer pair (Supplementary Table 9) and verified through sequencing. Additional mutations were introduced into ACP_1_ and ACP_7_ to allow visualization of the carrier proteins by UV/vis, enabling purification by FPLC and concentration estimation by NanoDrop. The second and third residues (after the start codon) in ACP_1_ were modified to Tyr and Trp, respectively, and the second residue (after the start codon) in ACP_7_ was modified to Trp and Tyr, via the Q5 site-directed mutagenesis kit (Supplementary Table 9). The PKAL-1 construct was transformed into BL21 (DE3) cells, and the cells were grown in LB broth with 150 mg/L ampicillin at 37 °C to OD_600_ 0.6–0.8, cooled down on the ice for 20 min, and induced with 0.3 mM IPTG at 16 °C for 20 h. The ACP_1_ and ACP_7_ constructs were each co-transformed with pACYCDuet-sfp into BL21 (DE3) for co-expression. The cells were grown in LB broth with 150 mg/L ampicillin and 34 mg/L chloramphenicol at 37 °C to OD_600_ 0.3–0.4, and the temperature was lowered to 16 °C for expression. Totally, 30 min prior to induction, cultures were supplemented with 2.5 mM calcium pantothenate, and once cells reached OD_600_ 0.6–0.8, protein expression was induced with 0.6 mM IPTG at 16 °C for 20 h. All purification steps were carried out at 4 °C. Briefly, cells were collected by centrifugation at 3000 × *g* for 10 min, and resuspended in lysis buffer (20 mM Tris, 500 mM NaCl, pH 7.5). The cells were then lysed by a microfluidizer three times and centrifuged at 20,000 × *g* for 20 min. The supernatant was incubated with 1 mL of pre-equilibrated Nickel-resin (Thermo Scientific) for 1 h by shaking on ice. The resin was washed with 15 mL of lysis buffer, 15 mL of wash buffer (20 mM Tris, 500 mM NaCl, 20 mM imidazole, pH 7.5), and eluted with wash buffer containing 250 mM imidazole. During the purification of PKAL-1 for use in the kinetic assay, 1 mM ATP was included in the wash buffer^48^. For PKAL-1, the eluted sample was concentrated and loaded onto an FPLC connecting to a Superdex 200 gel filtration column (GE healthcare) with buffer (20 mM Tris, 100 mM NaCl, pH 7.5). Protein concentrations were determined by using Quick Start Dye reagent (Bio-Rad) with 2 mg/mL bovine serum albumin used as a standard (for PKAL-1) or by Nanodrop (for the carrier proteins). Purified proteins were flash-frozen in 10% glycerol and stored at −80 °C. Mass analysis of the carrier proteins expressed individually compared to co-expression with Sfp showed complete conversion of the carrier proteins from the apo to the holo form.

### LC–MS-based PKAL-1 activity assay

To examine the activity of PKAL-1 and the PKAL-1(K488A) mutant, an LC–MS-based assay was used^49^. A 50 µL reaction mixture contained 100 mM potassium phosphate at pH 7.0, 5 mM MgCl_2_, 5 mM CoA, 5 mM ATP, 0.1% Triton X-100, and 1 mM fatty acid substrate. The reaction was initiated by adding 2 µL of 2 mg/mL purified PKAL-1 or PKAL-1(K488A) enzyme at 25 °C for 2 h. 50 µL methanol was added to quench the reaction, and the reaction was vortexed and centrifuged. A 5 µL supernatant was used for LC–MS analysis on an Agilent 6130 single quadrupole mass spectrometer in both positive and negative full-scan modes, mass range 150–1500, 125 V fragmentor voltage, 0.15 min peak width, and 2.20 s cycle length. Mobile phase A was water with 10 mM ammonium acetate, and mobile phase B was acetonitrile. The LC gradient was started from 95% A for 2 min and then ramped up to 100% B over 24 min.

### MALDI-TOF MS analysis

A 15 µL reaction mixture contained 100 mM Tris buffer at pH 7.8, 10 mM MgCl_2_, 1 mM TCEP, 1 mM ATP, 100 μM fatty acid substrate, and 100 µM holo-ACP_7_ or 100 µM holo-ACP_1_. The reactions were initiated by adding 2 µL of 2 mg/mL purified PKAL-1 or PKAL-1(K488A) at 25 °C for 2 h. Samples were diluted 1:10 in ultrapure water and spotted onto a ground plate 1:1 with a matrix containing saturated sinapinic acid in 70% acetonitrile. A Bruker AutoFlex LRF MALDI-TOF (Bruker Daltonics) equipped with a Smartbeam-II UV laser was used to analyze the ACP masses, using the positive linear mode at a mass range of 5000–20,000 Da. Laser power was used at the threshold level required to generate the signal until suitable data were obtained. The instrument was calibrated with Protein Calibration Standard I (Bruker Daltonics) bracketing the molecular weights of the samples (typically, mixtures of apo myoglobin and bovine serum albumin using doubly charged, singly charged, and dimer peaks, as appropriate). All data were analyzed using flexAnalysis software (Bruker). Every single spectrum was an average of 500 laser shots, and the final spectra were generated using the sum of at least three single spectra.

### Enzyme-coupled continuous kinetic assay for PKAL-1

The enzyme kinetics of PKAL-1 were determined through an enzyme-coupled spectrophotometric assay^35^. Each 100 μL assay mixture contained 100 mM Tris buffer pH 7.4, 1 mM dithiothreitol, 10 mM MgCl_2_, 4 mM ATP, 0.9 mM phosphoenolpyruvate, 0.3 mM NADH, 2.5 U pyruvate kinase, 3.5 U lactate dehydrogenase, 10 U adenylate kinase (Sigma M3003, prepared according to manufacturer’s protocol), 100 mM buffered hydroxylamine, and the tested substrates in DMSO (final volume 2.5%). The kinetic assay was initiated by the addition of an enzyme and run at 22 °C. No activity was detected in the assay when initiating with PKAL-1(K488A). GraphPad Prism was used to calculate the apparent kinetic constants.

### Reporting summary

Further information on research design is available in the Nature Research Reporting Summary linked to this article.

## Supplementary information

Supplementary Information

Reporting Summary

## Data Availability

The LC–MS/MS for **3**, **4**, **5**, **6**, **7**, and **8** have been deposited in the MetaboLights^50^ database under accession code MTBLS2313. Source data are provided with this paper. All other data supporting the findings of this study are available within the article and its Supplementary Information files or from the corresponding author upon request. Source data are provided with this paper.

## Source data

Source Data

## References

[1] Torres JP, Schmidt EW (2019). The biosynthetic diversity of the animal world. J. Biol. Chem..

[2] Ganley, J. G. & Derbyshire, E. R. Linking genes to molecules in eukaryotic sources: an endeavor to expand our biosynthetic repertoire. *Molecules* **25**, 625 10.3390/molecules25030625 (2020). 10.3390/molecules25030625 PMC7036892 32023950

[3] O’Brien RV, Davis RW, Khosla C, Hillenmeyer ME (2014). Computational identification and analysis of orphan assembly-line polyketide synthases. J. Antibiot..

[4] Wang H, Fewer DP, Holm L, Rouhiainen L, Sivonen K (2014). Atlas of nonribosomal peptide and polyketide biosynthetic pathways reveals common occurrence of nonmodular enzymes. Proc. Natl Acad. Sci. USA.

[5] Nivina A, Yuet KP, Hsu J, Khosla C (2019). Evolution and diversity of assembly-line polyketide synthases. Chem. Rev..

[6] Richardt A (2003). Ebony, a novel nonribosomal peptide synthetase for beta-alanine conjugation with biogenic amines in Drosophilia. J. Biol. Chem..

[7] Osborn, A. R. et al. De novo synthesis of a sunscreen compound in vertebrates. *Elife* **4**, e05919 10.7554/eLife.05919 (2015). 10.7554/eLife.05919 PMC4426668 25965179

[8] Shou Q (2016). A hybrid polyketide-nonribosomal peptide in nematodes that promotes larval survival. Nat. Chem. Biol..

[9] Cooke TF (2017). Genetic mapping and biochemical basis of yellow feather pigmentation in budgerigars. Cell.

[10] Lancaster J (2018). *De novo* formation of an aggregation pheromone precursor by an isoprenyl diphosphate synthase-related terpene synthase in the harlequin bug. Proc. Natl Acad. Sci. USA.

[11] Torres JP, Lin Z, Winter JM, Krug PJ, Schmidt EW (2020). Animal biosynthesis of complex polyketides in a photosynthetic partnership. Nat. Commun..

[12] Butcher RA (2019). Natural products as chemical tools to dissect complex biology in *C. elegans*. Curr. Opin. Chem. Biol..

[13] Baugh LR (2013). To grow or not to grow: nutritional control of development during *Caenorhabditis elegans* L1 arrest. Genetics.

[14] Fischbach MA, Walsh CT (2006). Assembly-line enzymology for polyketide and nonribosomal Peptide antibiotics: logic, machinery, and mechanisms. Chem. Rev..

[15] Khosla C, Herschlag D, Cane DE, Walsh CT (2014). Assembly line polyketide synthases: mechanistic insights and unsolved problems. Biochemistry.

[16] Miyanaga A, Kudo F, Eguchi T (2018). Protein-protein interactions in polyketide synthase-nonribosomal peptide synthetase hybrid assembly lines. Nat. Prod. Rep..

[17] Rottig M (2011). NRPSpredictor2–a web server for predicting NRPS adenylation domain specificity. Nucleic Acids Res..

[18] Balibar CJ, Vaillancourt FH, Walsh CT (2005). Generation of D amino acid residues in assembly of arthrofactin by dual condensation/epimerization domains. Chem. Biol..

[19] Weber T (2015). antiSMASH 3.0—a comprehensive resource for the genome mining of biosynthetic gene clusters. Nucleic Acids Res.

[20] Waterhouse A (2018). SWISS-MODEL: homology modelling of protein structures and complexes. Nucleic Acids Res..

[21] Horsman ME, Hari TP, Boddy CN (2016). Polyketide synthase and non-ribosomal peptide synthetase thioesterase selectivity: logic gate or a victim of fate?. Nat. Prod. Rep..

[22] Kotowska M, Pawlik K (2014). Roles of type II thioesterases and their application for secondary metabolite yield improvement. Appl. Microbiol. Biotechnol..

[23] Cao J (2017). Comprehensive single-cell transcriptional profiling of a multicellular organism. Science.

[24] Hutter H, Ng MP, Chen N (2009). GExplore: a web server for integrated queries of protein domains, gene expression and mutant phenotypes. BMC Genomics.

[25] Perez E (2004). Molecular dissection of the role of two methyltransferases in the biosynthesis of phenolglycolipids and phthiocerol dimycoserosate in the *Mycobacterium tuberculosis* complex. J. Biol. Chem..

[26] Purwantini E, Daniels L, Mukhopadhyay B (2016). F_420_H_2_ is required for phthiocerol dimycocerosate synthesis in mycobacteria. J. Bacteriol..

[27] Wang N (2018). Natural separation of the acyl-CoA ligase reaction results in a non-adenylating enzyme. Nat. Chem. Biol..

[28] Trivedi OA (2004). Enzymic activation and transfer of fatty acids as acyl-adenylates in mycobacteria. Nature.

[29] Arora P (2009). Mechanistic and functional insights into fatty acid activation in *Mycobacterium tuberculosis*. Nat. Chem. Biol..

[30] Leger M (2009). The dual function of the *Mycobacterium tuberculosis* FadD32 required for mycolic acid biosynthesis. Chem. Biol..

[31] Liu Z, Ioerger TR, Wang F, Sacchettini JC (2013). Structures of *Mycobacterium tuberculosis* FadD10 protein reveal a new type of adenylate-forming enzyme. J. Biol. Chem..

[32] Galica T, Hrouzek P, Mares J (2017). Genome mining reveals high incidence of putative lipopeptide biosynthesis NRPS/PKS clusters containing fatty acyl-AMP ligase genes in biofilm-forming cyanobacteria. J. Phycol..

[33] Harris NC (2017). Biosynthesis of isonitrile lipopeptides by conserved nonribosomal peptide synthetase gene clusters in Actinobacteria. Proc. Natl Acad. Sci. USA.

[34] Lambalot RH (1996). A new enzyme superfamily—the phosphopantetheinyl transferases. Chem. Biol..

[35] Drake EJ (2016). Structures of two distinct conformations of holo-non-ribosomal peptide synthetases. Nature.

[36] Estrada P (2017). The pimeloyl-CoA synthetase BioW defines a new fold for adenylate-forming enzymes. Nat. Chem. Biol..

[37] Jorgensen H (2009). Biosynthesis of macrolactam BE-14106 involves two distinct PKS systems and amino acid processing enzymes for generation of the aminoacyl starter unit. Chem. Biol..

[38] Jorgensen H (2010). Insights into the evolution of macrolactam biosynthesis through cloning and comparative analysis of the biosynthetic gene cluster for a novel macrocyclic lactam, ML-449. Appl. Environ. Microbiol..

[39] Amagai K, Takaku R, Kudo F, Eguchi T (2013). A unique amino transfer mechanism for constructing the beta-amino fatty acid starter unit in the biosynthesis of the macrolactam antibiotic cremimycin. Chembiochem.

[40] Kudo F, Miyanaga A, Eguchi T (2014). Biosynthesis of natural products containing β-amino acids. Nat. Prod. Rep..

[41] Uno M (2013). A fasting-responsive signaling pathway that extends life span in *C. elegans*. Cell Rep..

[42] Hibshman, J. D. et al. *daf-16*/FoxO promotes gluconeogenesis and trehalose synthesis during starvation to support survival. *Elife* **6**, e30057 10.7554/eLife.30057 (2017). 10.7554/eLife.30057 PMC5655125 29063832

[43] Arribere JA (2014). Efficient marker-free recovery of custom genetic modifications with CRISPR/Cas9 in *Caenorhabditis elegans*. Genetics.

[44] Cong L, Zhang F (2015). Genome engineering using CRISPR-Cas9 system. Methods Mol. Biol..

[45] Dokshin GA, Ghanta KS, Piscopo KM, Mello CC (2018). Robust genome editing with short single-stranded and long, partially single-stranded DNA donors in *Caenorhabditis elegans*. Genetics.

[46] Nass, R. & Hamza, I. The nematode *C. elegans* as an animal model to explore toxicology in vivo: solid and axenic growth culture conditions and compound exposure parameters. *Curr. Protoc. Toxicol*. Chapter 1, Unit1 9 (2007). 10.1002/0471140856.tx0109s31 20922756

[47] Zhang X (2015). Acyl-CoA oxidase complexes control the chemical message produced by *Caenorhabditis elegans*. Proc. Natl Acad. Sci. USA.

[48] Rial DV, Ceccarelli EA (2002). Removal of DnaK contamination during fusion protein purifications. Protein Expr. Purif..

[49] Zhou, Y. et al. Biosynthetic tailoring of existing ascaroside pheromones alters their biological function in *C. elegans*. *Elife* **7**, e33286 10.7554/eLife.33286 (2018). 10.7554/eLife.33286 PMC5986272 29863473

[50] Haug K (2020). MetaboLights: a resource evolving in response to the needs of its scientific community. Nucleic Acids Res..

