# Supplementary material for: Mapping the biosynthetic pathway of a hybrid polyketide-nonribosomal peptide in a metazoan
Source: Nat Commun. 2021 Aug 13;12:4912. doi: 10.1038/s41467-021-24682-9 (PMC8363725; doi:10.1038/s41467-021-24682-9)
Supplement: Supplementary file 1 — Supplementary Information [file 41467_2021_24682_MOESM1_ESM.pdf]

Supplementary Information

Mapping the biosynthetic pathway of a hybrid polyketide-nonribosomal peptide in a metazoan

Likui Feng[1,2], Matthew T. Gordon[1], Ying Liu[1], Kari B. Basso[1], Rebecca A. Butcher[1*]

[1]Department of Chemistry, University of Florida, Gainesville, FL, 32611
[2]Present address: Lulu and Anthony Wang Laboratory of Neural Circuits and Behavior, The Rockefeller University, New York, NY 10065
[*]Correspondence to: butcher@chem.ufl.edu

a Thioesterase (TE) domains

```
PKS-1_TE1      .....DLKYPSNDLRELAHFYAEEIAA...HAGNKRIFVMGHSMGGIMSREIVAELKIWG
NRPS-1_TE2     ..........GDTVDEVAKLYRLQIEESAENIETSKLVFIGASSAGTFAFSTSQLFADDD
Pik_TE         GTGTGTALLPADLDTALDAQARAILR....AAGDAPVVLLGHSGGALLAHELAFRLERAH
Sur_TE         NQ.TS....AIEDLEELTDLYKQELN....LRPDRPFVLFGHSMGGMITFRLAQKLEREG
Ybt_TE         LRHLE....PLRSITQLAALLANELEA..SVSPDTPLLLAGHSMGAQVAFETCRLLEQRG
Rif_TE         RRHEP....PVDSIGGLTNRLLEVLR....PFGDRPLALFGHSMGAIIGYELALRMPEAG
                                                         *
```

b Condensation (C) domains

```
NRPS-1_C2      .............EKSSVSNPLSAI...QCSKLSPETLQLIFHHISIDGRSLAIFYQQFK
NRPS-1_C3      ..............PSGASRPKDQFSIQLWMSSKNKLLTISIHHLICDGRSLQILEHQLQ
NRPS-1_C4      IR.LLCDEPINV....LEGSPMIRASFI..SSPEKHVAFLHLHHLISDARSTQLTNSTMK
PKS-1_C1       .........NHLFE..IGKSTPLRVRVAEDCDNSRIHIVFNQHHILTDGWSMTVLSDTVS
ArfA-C2        FSARR....Y...RLDVSQAPLMRLVYARDPALDRVVGILLFHHLAMDHIALEV....MR
VibH_C         QIEQDLQRSSTLIDAPITSHQVYRLSHS......EHLIYTRAHHIVLDGYGMMLFEQRLS
CDA_C1         WMDRDRATPLPLDRPGLSSHALFTLGGG......RHLYYLGVHHIVIDGTSMALFYERLA
                                                          *   *
```

c Acyl-carrier protein (ACP) domains

```
PKS-1_ACP3     ....KTLQMAVRHKVCLAVGDVIESGLDIDESQLSTGFSE.LGIDSLATVDLLNRLNQKY
PKS-1_ACP4     ESDATVDRTEIRRKVSLAVFDLATETLSAEDLQ.SKGFTE.LGMDSLSIVDFVNRLNDKY
PKS-1_ACP6     ....KVKEEIKKKSLNFEEIFFEIVGITDISSKLNIPFMD.LGIDSLCMENLRYSLNKN.
Bac_ACP        ..........ADTLERVTKIIVDRLGVDEADVKLEASFKEDLGADSLDVVELVMELEDE.
PKS-1_ACP5     ...MNFSVEDEEEVLELIKEKVSSILMCSPTKLKNNKNIMDMGLDSKLIVEFLNFINST.
NRPS-1_ACP7    .....CTARPSRSMEILISILKDQMKLLSTSHEVETTPLPYLGIDSLRLAELEYHVASH.
PKS-1_ACP1     .NIVDEQTNSSLSDAEIESTVRTIVKQFLDIEEDDINLLETGAVDSLTSIEMVEAFGTAV
PKS-1_ACP2     .KITKKVENEDQKRASKNMLHVWFEENFGWTDIDNTTGFFDLGLTSIQAVKLRNAIKSN.
                                                            *
```

d Adenylation (A) domains

```
EntE_A         FFALLKLGVAPVL..ALFSHQRSELNAYASQIEPALLIADRQH....ALFSGDDFLNTFV
PKS-1_A1       LLACVFLGLPYAPIDPTWPEPRQLFVKSKVSFTLE....N...............CF...
SidN_A3        IVGIMKSGNTYVPIEAGLPNDRKSFLLRDSRAAMAFVCDN........NFDGVELPP...
Grs_A          ILAVLKAGGAYVPIDIEYPKERIQYILDDSQARMLLTQKHLVHLIHNIQFNGQVEIF...
NRPS-1_A2      VLAAWEAGLYPVPMHKDSKEAQIEKTLEALGIEEA..............FDSKDLCQ...
NRPS-1_A3      LAVQFT.GAAYLPIDASYPEERKKTILKDSVFNFE..............YNGRVDQE...

EntE_A         TEHSSIRVVQLLNDSGEHNLQDAINHPAEDFTATPSPADEVAYFQLSGGTTGTPKLIPRT
PKS-1_A1       ........................SCNLKLRNFNSRTQFGSIYSIFTSGSTGVPKGVLMA
SidN_A3        .ET...KV...LD.TKNQSFIENLSTQDTSDILNNYPENLDAYLLYTSGSTGTPKGVRVS
Grs_A          .EE...DT...IK............IREGTNLHVPSKSTDLAYVIYTSGTTGNPKGTMLE
NRPS-1_A2      ...........................TKLRIFNKSILYDLAYVTSTSGSTGTPKLVGTS
NRPS-1_A3      ...........................PRHRHFAISTDYCLSYIITTSGTTGTPKSVAIG
                                                               *

EntE_A         HNDYYYSVRRSVE........ICQFTQQTRYLCAIPAAHNYAMSSPGSLGVFLAGGTVVL
PKS-1_A1       EQSVSSFMTSASK........QCMFRSNIRVLDSVKQVFD..VSVSNIIGSVLNGGVLIS
SidN_A3        RHNLSSFSDAWGKLIGNVAPKSLELGGVGKFLCLASRAFD..VHIGEMFLAWRFGLCAVT
Grs_A          HKGISNLKVFFEN........SLNVTEKDRIGQFASISFD..ASVWEMFMALLTGASLYI
NRPS-1_A2      FEGHSNLARQYTT........TYQISSRDTVGQVVDPSFD..IFFADIVK......TLVN
NRPS-1_A3      AKSLLNLFLSSTL........TMKCSSSSRTYQFTNFVFD..NSVLEVSMSIASQGTLVY
```

e Peptidyl-carrier protein (PCP) domains

```
NRPS-1_PCP3    .LPHPTLPNSLESDLSSIWTSLLNCPEPSPSDH.FFLIGGHSLLLVRLRHLIETKLGISL
NRPS-1_PCP1    KAAISIARLSLTSSLKHWLEHYSNLEIPSDDDD.IFTLGVDSIAVMLAMQKLRSEN.IEI
PKS-1_PCP2     KREIVVMKNSLEEKVINVFSKILGR.NVAPTDK.FESIGGNSLNAIQIAHRLAEELKIEI
NRPS-1_PCP2    KLLGLVEVRKKLELIIAAFKKFLDNTDVTKSTD.FFQAGGHSLTAMRLIDHLSDLLEVEI
Yer_PCP1       .AEADLPQGDIEKQVAALWQQLLSTGNVTRETD.FFQQGGDSLLATRLTGQLHQAG.YEA
PKS-1_PCP1     ESLKLPKSTSCEFVIAEIWKETLGISILNDANPNFFSLGGDSLSALQVVWKVQKKTDRIV
                                                        *
```

Supplementary Figure 1. Domain sequence alignments of PKS-1 and NRPS-1 with known functional domains. **a** Sequence alignment of TE domains with Pik_TE (Pikromycin TE domain, PDB ID: 2H7Y), Sur_TE (Surfactin TE domain, PDB ID: 2RON), Yer_TE (Yersiniabactin TE domain, PDB ID: 6BA8), and Rif_TE (Rifamycin TE domain, PDB ID: 3FLA). Sequences used for TE domains are PKS-1_TE_1 (7559-7610) and NRPS-1_TE_2 (2771-2820). The catalytic serine residue is labeled with an asterisk. **b** Sequence alignment of C domains with ArfA_C_2 (Arthrofactin module A C_2 domain), VibH_C (Vibriobactin free-standing C domain VibH, PDB ID: 1L5A), CDA_C_1 (Calcium-dependent antibiotic synthase C_1 domain, PDB ID: 4JN3). Sequences used for C domains are PKS-1_C_1 (6653-6701), NRPS-1_C_2 (520-563), NRPS-1_C_3 (1457-1502), and NRPS-1_C_4 (1895-1947). In the HHxxxDG motif, the second histidine (asterisk) serves as a catalytic base, and the aspartate (asterisk) is critical for the structural integrity of the active site. **c** Sequence alignment of NRPS-1_ACP_7 with PKS-1_ACP domains and *Bacillus subtilis* ACP (GenBank accession no. P80643, PDB ID: 1HY8). The conserved active site serine is marked with asterisk. Protein sequences used are PKS-1_ACP_1 (719-776), PKS-1_ACP_2 (1776-1833), PKS-1_ACP_3 (2792-2846), PKS-1_ACP_4 (2940-2997), PKS-1_ACP_5 (3471-3526), PKS-1_ACP_6 (5154-5207), and NRPS-1_ACP_7 (267-320). **d** Sequence alignment of A domains with EntE_A (Enterobactin module E, PDB ID: 3RG2), SidN_A_3 (A_3 domain in Siderophore N synthetase, PDB ID: 3ITE), Grs_A (Gramicidin S, PDB ID: 1AMU). Sequences used for A domains are PKS-1_A_1 (7044-7167), NRPS-1_A_2 (900-1019), and NRPS-1_A_3 (2274-2398). The conserved glycine residue in the flexible loop involved in the interaction with the pyrophosphate leaving group during amino acid loading is marked with an asterisk.[1] **e** Sequence alignment of PKS-1_PCP domains, NRPS-1_PCP domains, and Yer_PCP_1 (Yersiniabactin synthetase PCP1 domain, GenBank accession no. Q7CI41, PDB ID: 5U3H). The serine residue for phosphopantetheinyl postranslational modification is located in the conserved PCP motif DXFFXLGGDSL and is marked with an asterisk. Protein sequences are PKS-1_PCP_1 (6462-6521), PKS-1_PCP_2 (7424-7481), NRPS-1_PCP_3 (1289-1346), NRPS-1_PCP_4 (1700-1758), and NRPS-1_PCP_5 (2648-2705). Alignment was generated by Clustal Omega 1.2.4 and ESPript 3.0.[2]

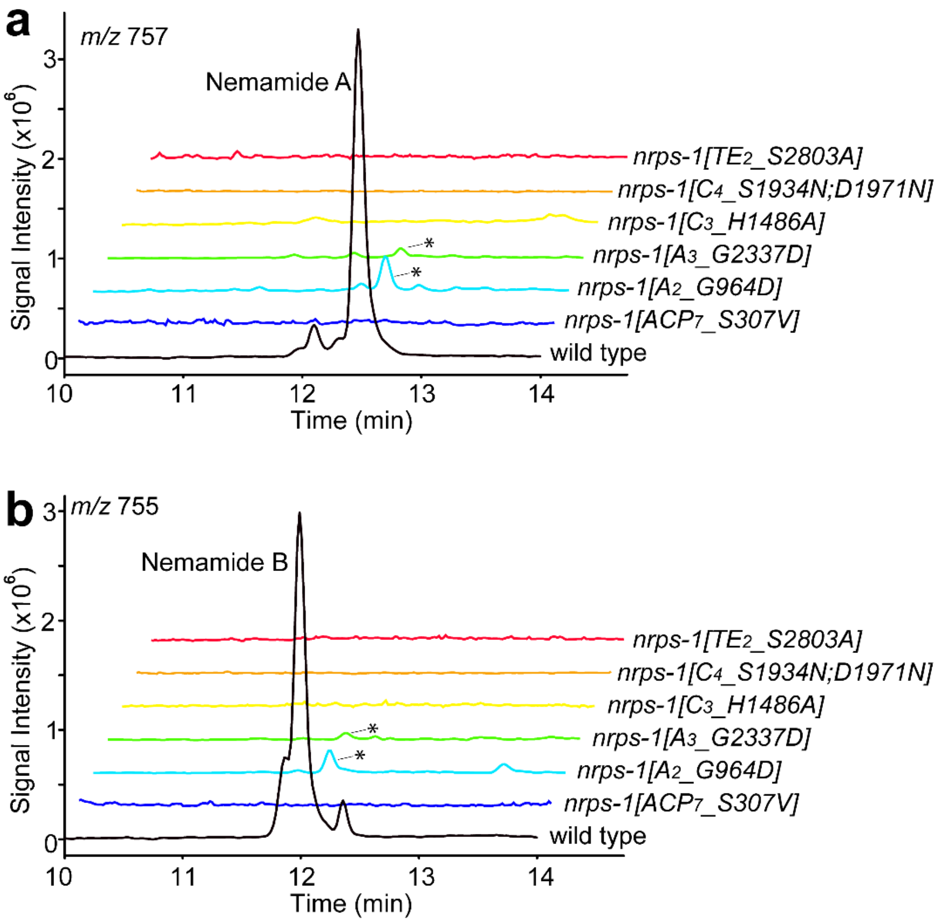

Supplementary Figure 2. Nemamide production in wild-type and *nrps-1* domain mutants. Extracted ion chromatograms for nemamide A (**a**) and nemamide B (**b**) in wild type, *nrps-1(reb8[ACP$_7$_S307V])*, *nrps-1(reb32[A$_2$_G964D])*, *nrps-1(reb31[A$_3$_G2337D])*, *nrps-1(reb10[C$_3$_H1486A])*, *nrps-1(gk186409[C$_4$_S1934N]; gk186410[C$_4$_D1971N]),* and *nrps-1(reb12[TE$_2$_S2803A])* worms. The *nrps-1(gk186409[C$_4$_S1934N]; gk186410[C$_4$_D1971N])* mutant was obtained from the Caenorhabditis Genetics Center and backcrossed four times with wild type. As indicated by the asterisks (*), both the *nrps-1(reb32[A$_2$_G964D])* and the *nrps-1(reb31[A$_3$_G2337D])* strains produced small amounts of nemamides; the *nrps-1(reb32[A$_2$_G964D])* strain produced 15.7±3.7% nemamide A relative to wild type, and the *nrps-1(reb31[A$_3$_G2337D])* strain produced 2.7±0.8% nemamide A relative to wild type, suggesting that the A domains in these mutants have some residual activity. Note that the retention times of the nemamides in the Supplementary Information are different than in the main text due to the fact that the samples were analyzed on different columns.

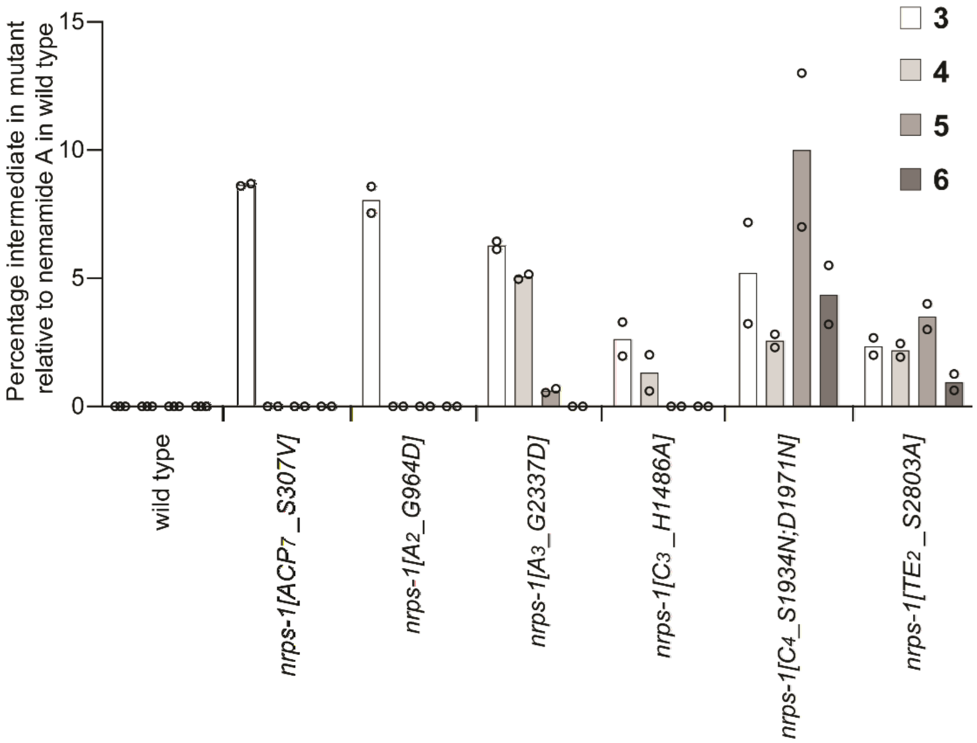

Supplementary Figure 3. Production of intermediates in wild type and *nrps-1* domain mutants. The production of nemamide A (**1**) and intermediates **3**, **4**, **5**, and **6** in wild type, *nrps-1(reb8[ACP$_7$_S307V])*, *nrps-1(reb32[A$_2$_G964D])*, *nrps-1(reb31[A$_3$_G2337D])*, *nrps-1(reb10[C$_3$_H1486A])*, *nrps-1(gk186409[C$_4$_S1934N]; gk186410[C$_4$_D1971N]),* and *nrps-1(reb12[TE$_2$_S2803A])* worms. The *nrps-1(gk186409[C$_4$_S1934N]; gk186410[C$_4$_D1971N])* mutant was obtained from the Caenorhabditis Genetics Center and backcrossed four times with wild type. Percentage for each intermediate in each strain was determined by comparing the amount of the intermediate (as gauged by UV at 280 nm) relative to the mean amount of nemamide A (as gauged by UV at 280 nm) in wild type. Data represent the mean of data points (indicated as open circles), n = 3 independent experiments for wild type and n = 2 independent experiments for all mutants. In each experiment, the worm strain was grown in large-scale culture, and the worms were collected for extraction of nemamide (for wild type) and intermediates (for wild type and mutants). Source data are provided as a Source Data file.

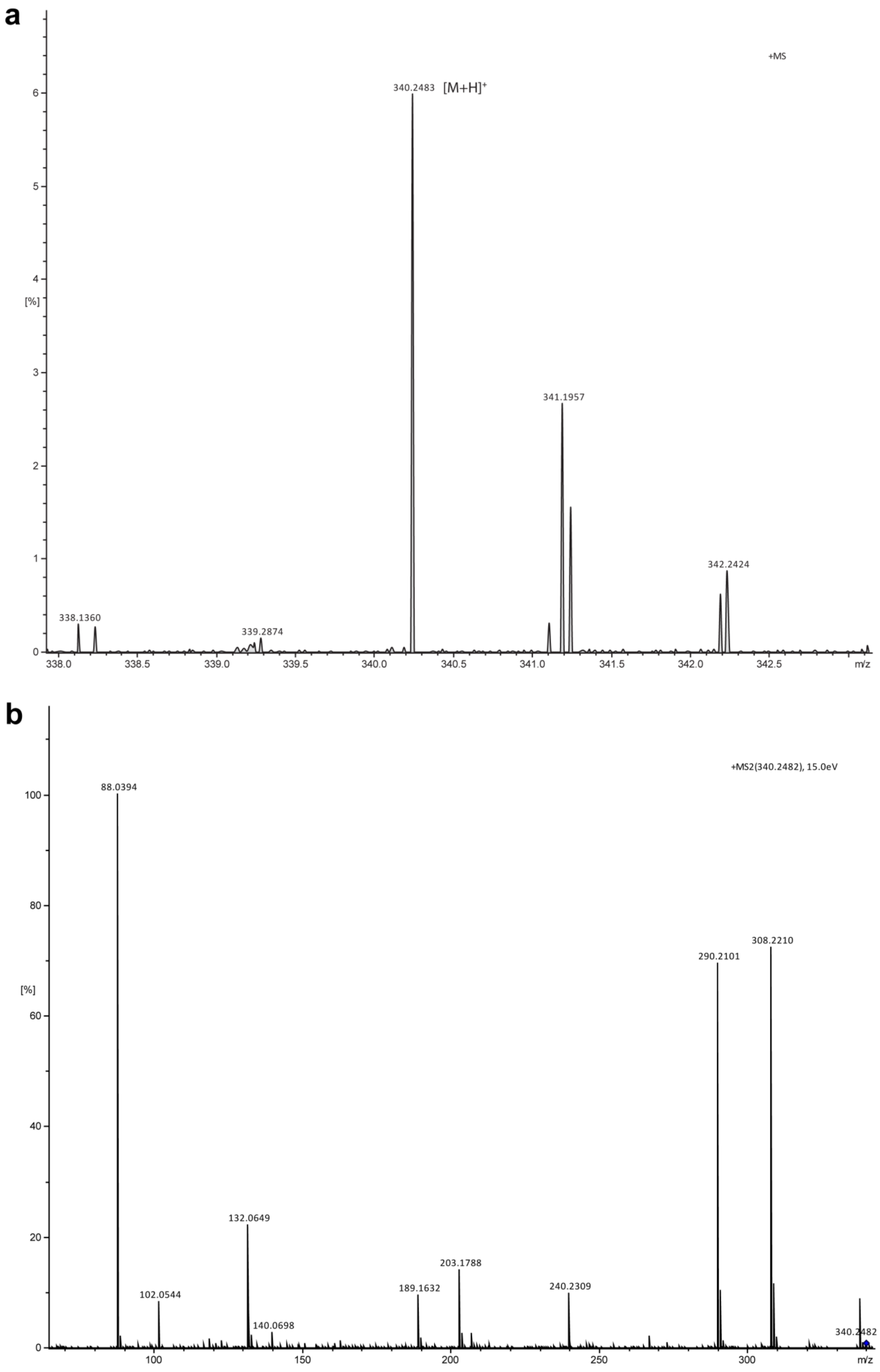
a
+MS
340.2483 [M+H]+
341.1957
342.2424
338.1360
339.2874
[%]
m/z
b
+MS2(340.2482), 15.0eV
88.0394
290.2101
308.2210
132.0649
102.0544
140.0698
189.1632
203.1788
240.2309
340.2482
[%]
m/z

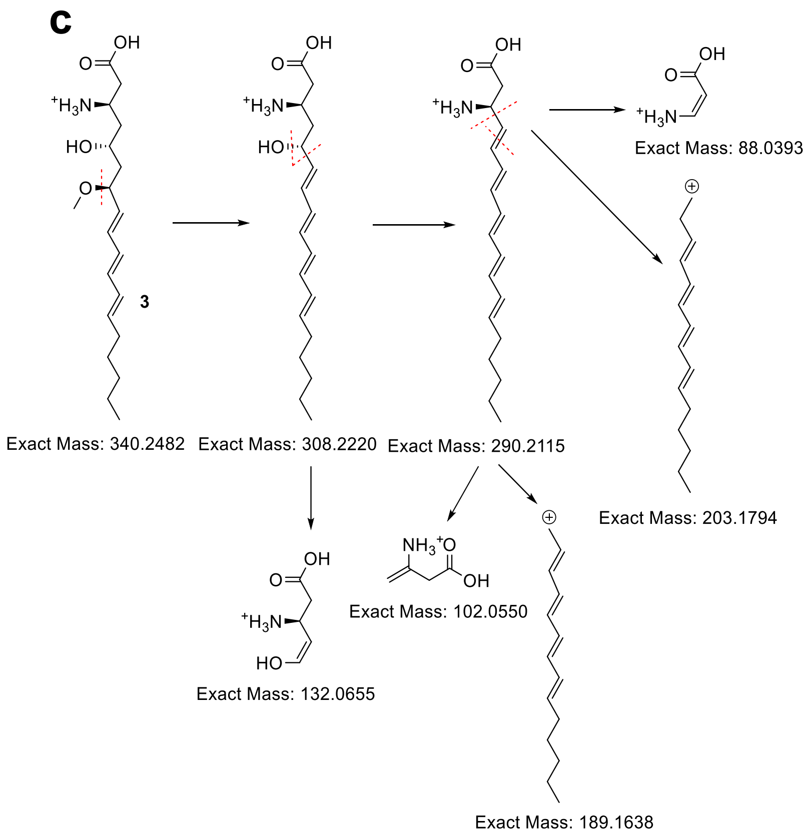

Supplementary Figure 4. Mass spectrometry analysis of intermediate 3. a,b HR-LC-MS/MS of intermediate **3**. **c** Analysis of the fragmentation patterns.

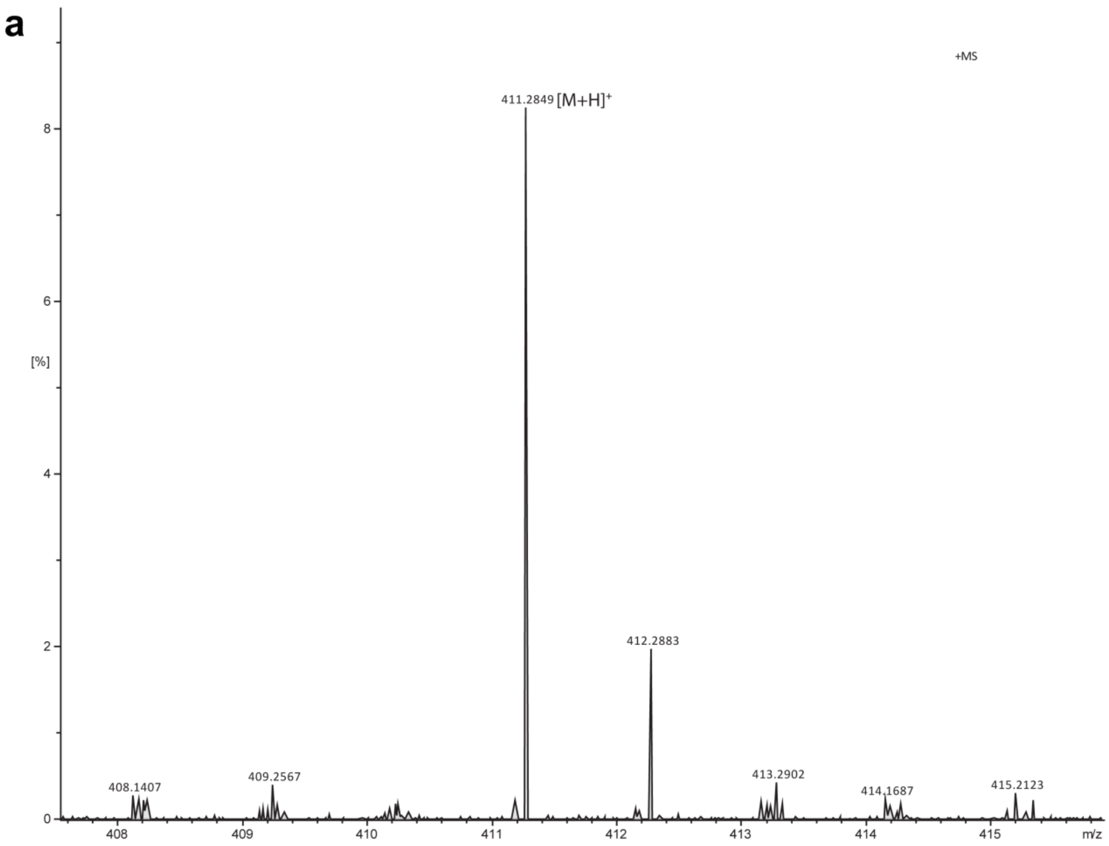
a
+MS
411.2849 [M+H]+
412.2883
408.1407
409.2567
413.2902
414.1687
415.2123
[%]
m/z

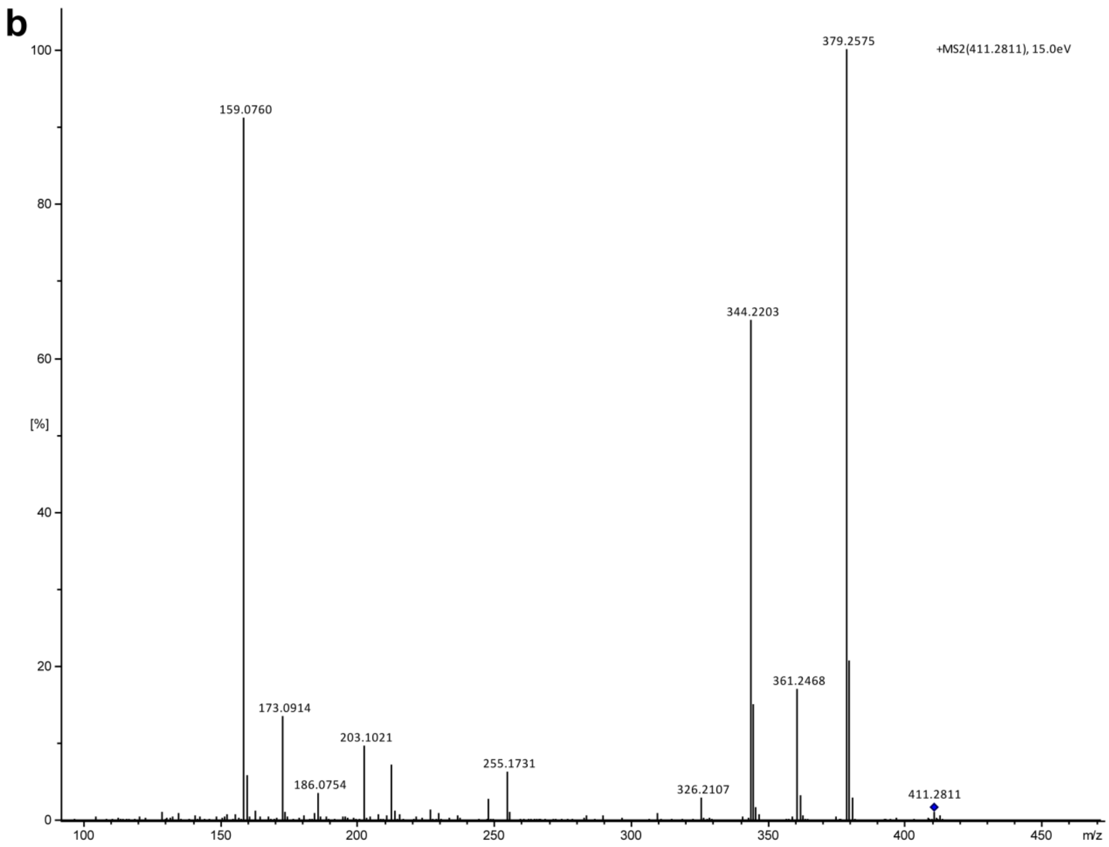
b
+MS2(411.2811), 15.0eV
379.2575
159.0760
344.2203
361.2468
173.0914
203.1021
186.0754
255.1731
326.2107
411.2811
[%]
m/z

c

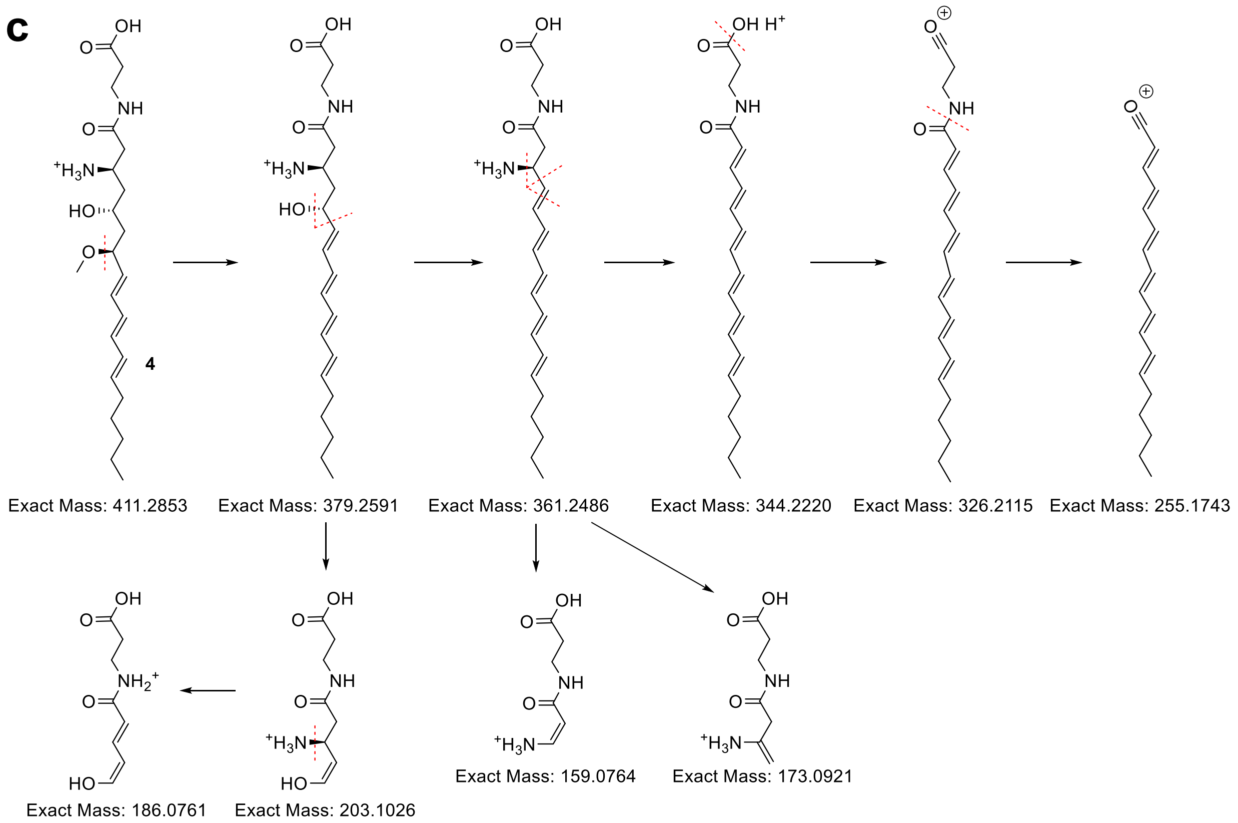

Supplementary Figure 5. Mass spectrometry analysis of intermediate 4. **a**,**b** HR-LC-MS/MS of intermediate **4**. **c** Analysis of the fragmentation patterns.

a

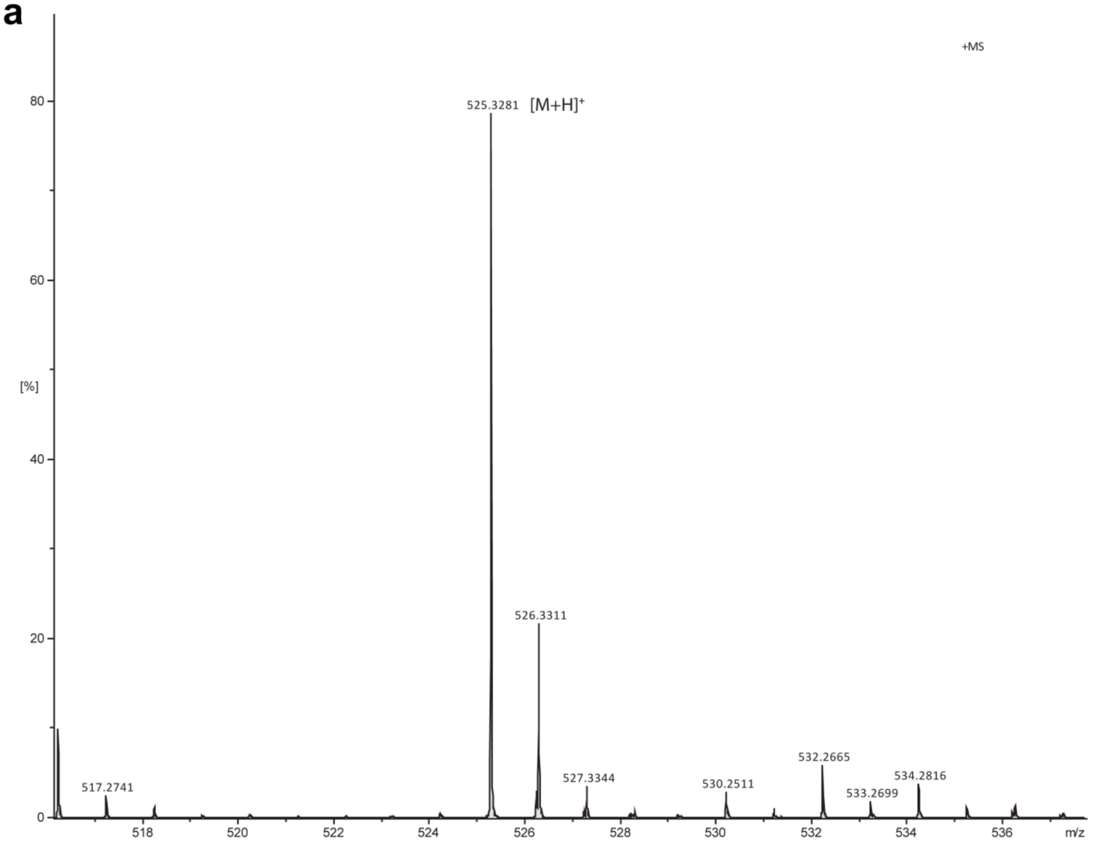
+MS
525.3281 [M+H]+
526.3311
517.2741
527.3344
530.2511
532.2665
533.2699
534.2816
[%]
m/z

b

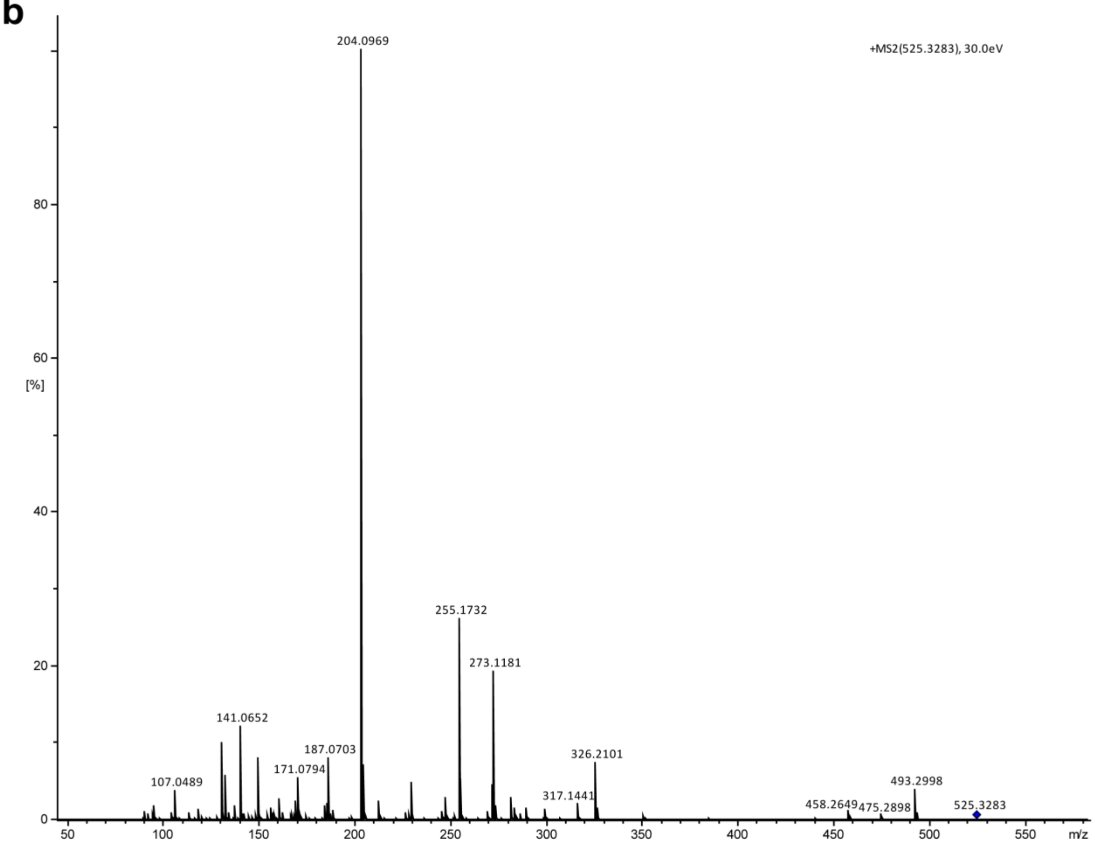
+MS2(525.3283), 30.0eV
204.0969
255.1732
273.1181
141.0652
187.0703
171.0794
107.0489
326.2101
317.1441
458.2649
475.2898
493.2998
525.3283
[%]
m/z

c

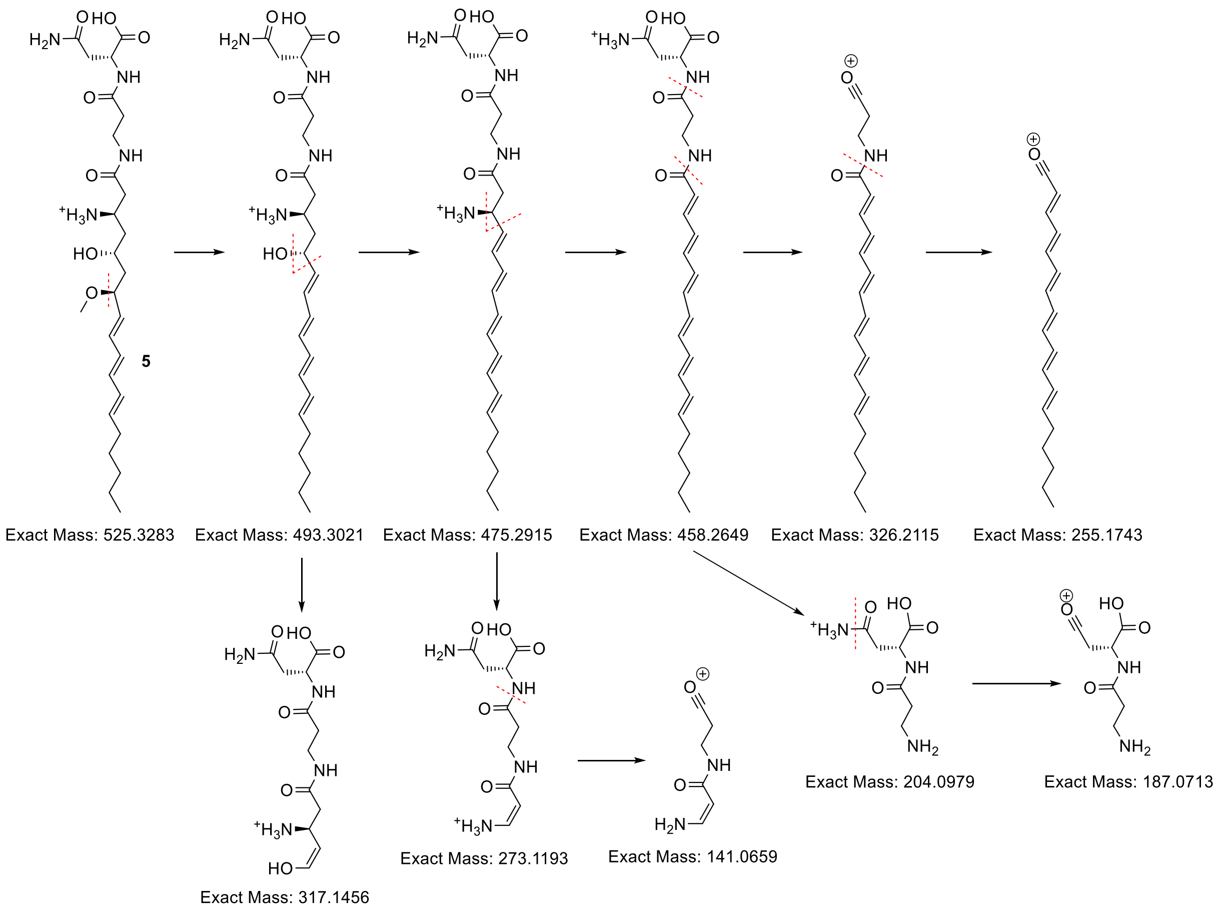

Supplementary Figure 6. Mass spectrometry analysis of intermediate 5. **a,b** HR-LC-MS/MS of intermediate **5**. **c** Analysis of the fragmentation patterns.

a

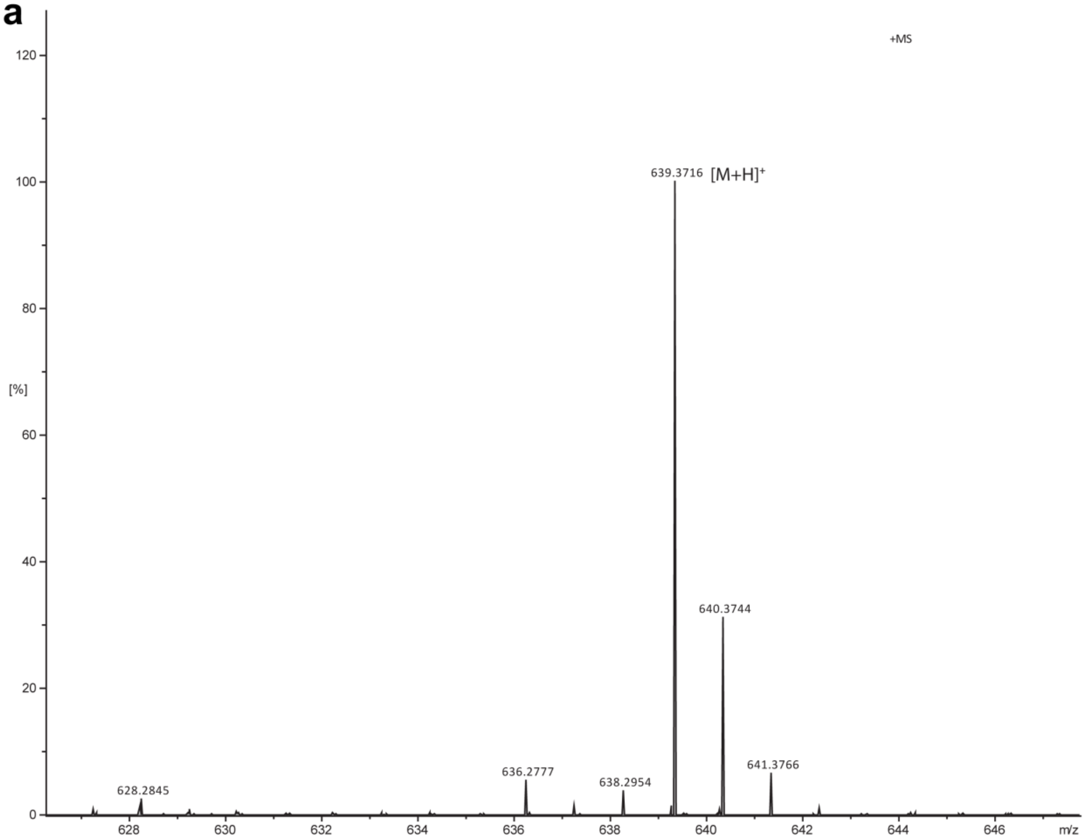
+MS
639.3716 [M+H]+
640.3744
641.3766
638.2954
636.2777
628.2845
[%]
m/z

b

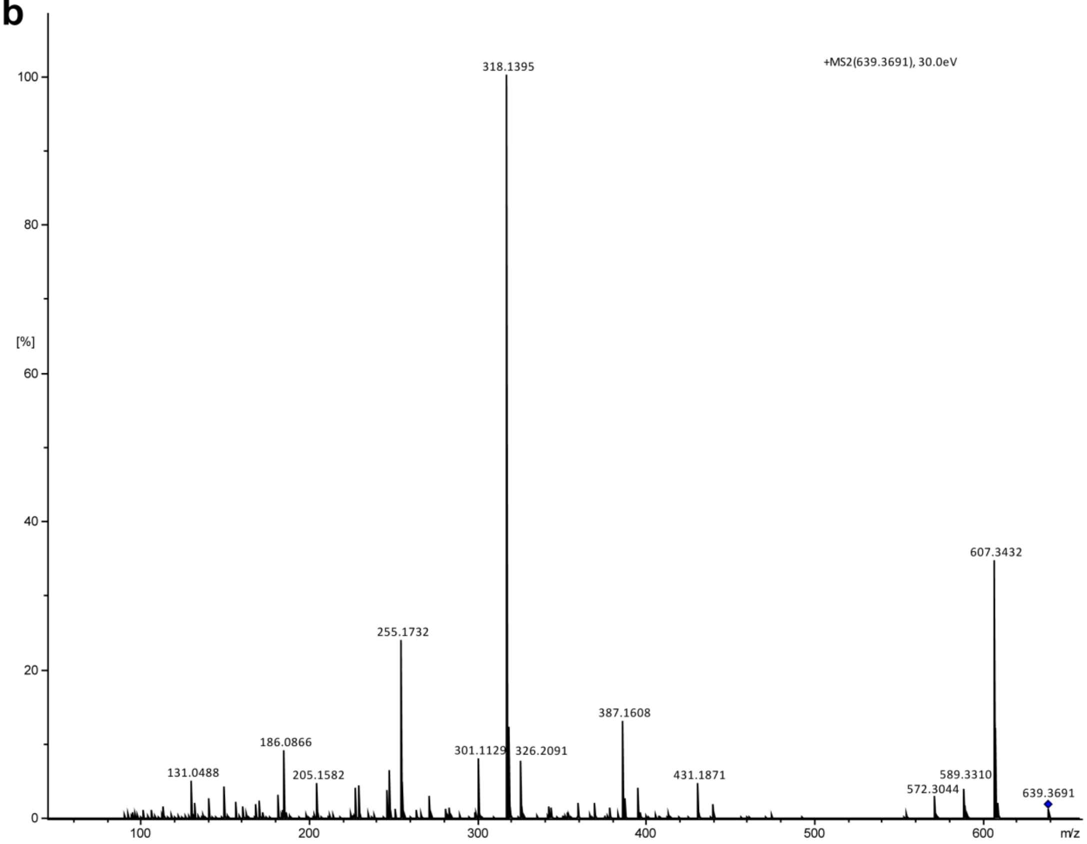
+MS2(639.3691), 30.0eV
318.1395
607.3432
255.1732
387.1608
186.0866
131.0488
205.1582
301.1129
326.2091
431.1871
572.3044
589.3310
639.3691
[%]
m/z

c

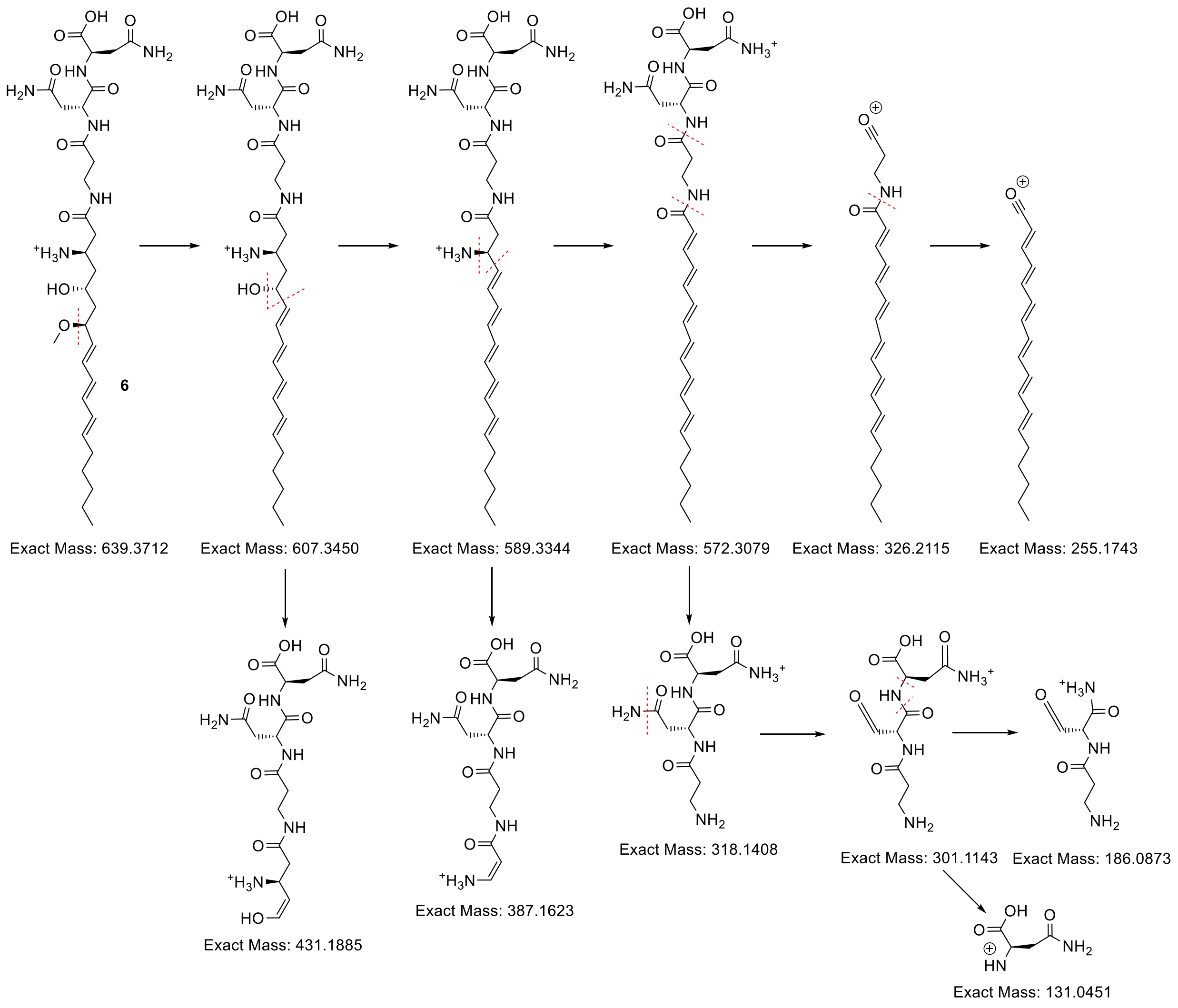

Supplementary Figure 7. Mass spectrometry analysis of intermediate 6. a,b HR-LC-MS/MS of intermediate **6**. **c** Analysis of the fragmentation patterns.

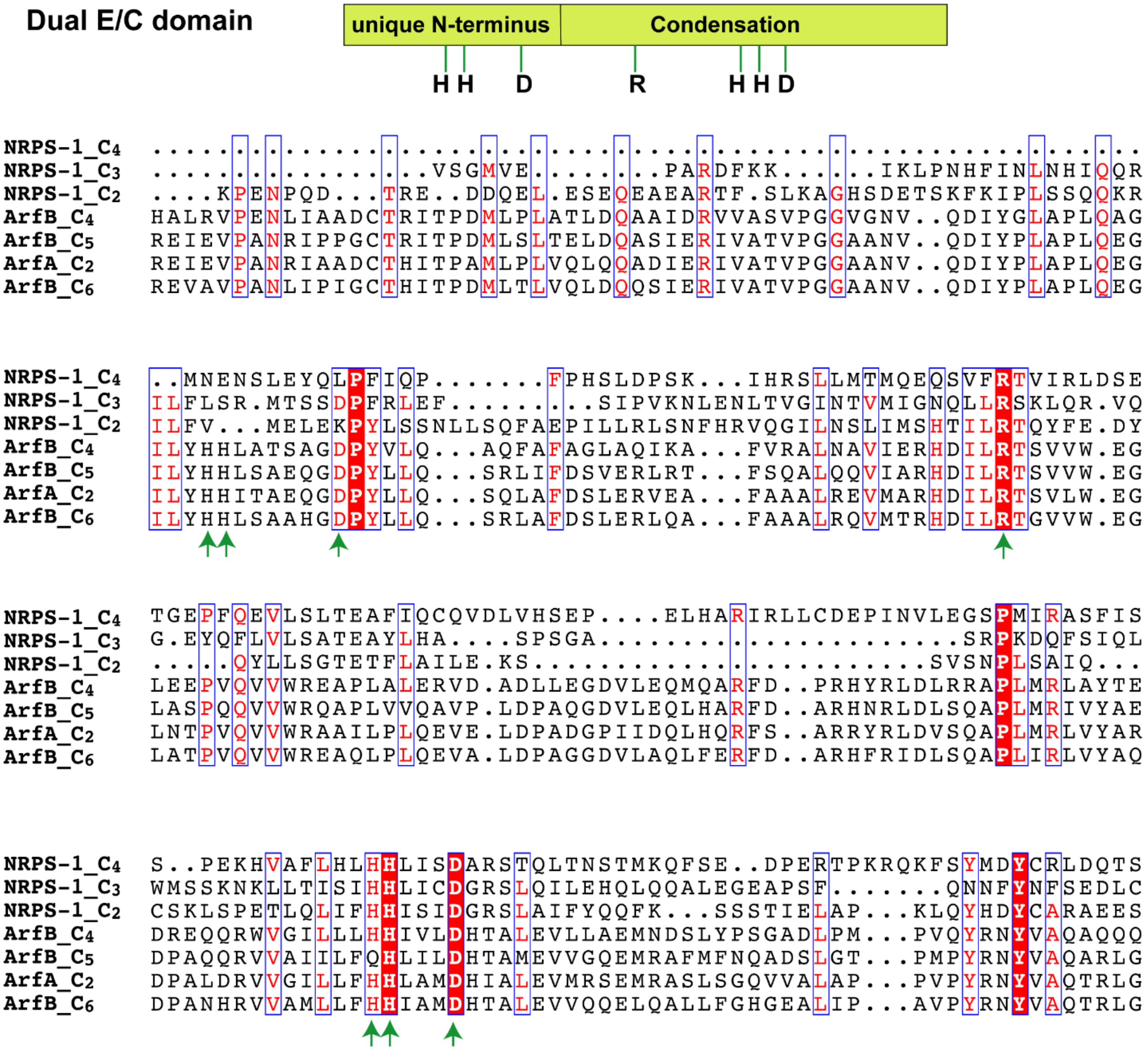

Supplementary Figure 8. Domain sequence alignments of the C domains in NRPS-1 with the dual epimerase (E)/C domains from the arthrofactin biosynthetic pathway. Dual E/C domains have a unique N-terminus with catalytic residues HH-D, followed by a common C domain with a catalytic motif R-HHXXXD.[3] The sequences of the C_2 domain from NRPS-1 (residues 401-586), the C_3 domain from NRPS-1 (residues 1361-1524), and the C_4 domain from NRPS-1 (residues 1815-1974) were compared to the sequences of the C domains of ArfB from the arthrofactin biosynthetic pathway. Essential residues for catalysis are indicated by arrows. The C domains in NRPS-1 are missing the catalytic residues in the N-terminus that are characteristic of dual E/C domains.

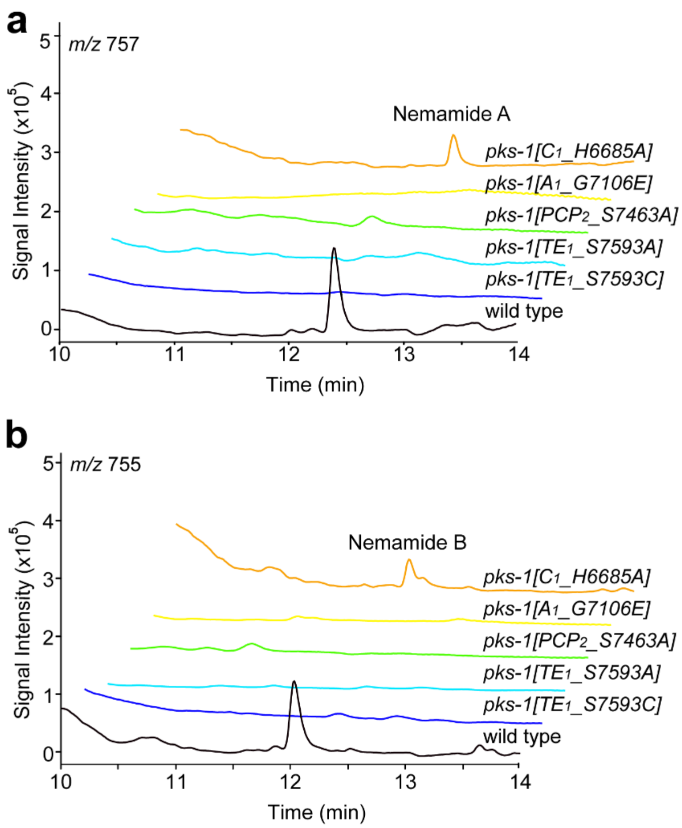

Supplementary Figure 9. Nemamide production in wild type and *pks-1* mutant strains. Extracted ion chromatogram for nemamide A (**a**) and nemamide B (**b**) in wild type and *pks-1(reb29[PCP$_2$_S7463A])*, *pks-1(reb22[A$_1$_G7106E])*, *pks-1(reb9[C$_1$_H6685A])*, *pks-1(reb11[TE$_1$_S7593A]),* and *pks-1(reb13[TE$_1$_S7593C])* mutant worms, containing mutations in the C-terminal NRPS module of PKS-1. Note that the nemamides could not be detected in the crude extracts of small-scale cultures of *pks-1(reb22[A$_1$_G7106E])* (as shown here), but they could be detected in very small amounts in the partially purified extracts of large-scale cultures of *pks-1(reb22[A$_1$_G7106E])* (as shown in Fig. 3). Note that the retention times of the nemamides in the Supplementary Information are different than in the main text due to the fact that the samples were analyzed on different columns.

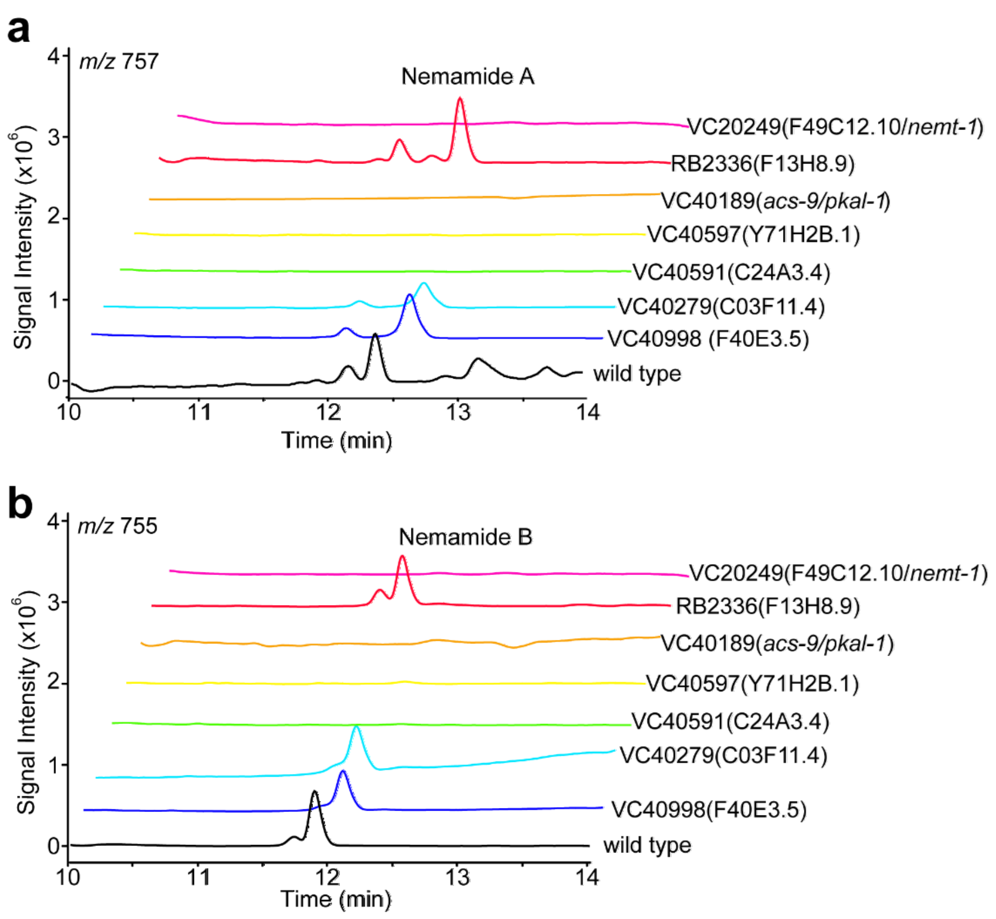

Supplementary Figure 10. Initial screen of available mutants for nemamide production. Extracted ion chromatogram for nemamide A (**a**) and nemamide B (**b**) in wild-type and the indicated mutant strains from Supplementary Table 2. Note that the retention times of the nemamides in the Supplementary Information are different than in the main text due to the fact that the samples were analyzed on different columns.

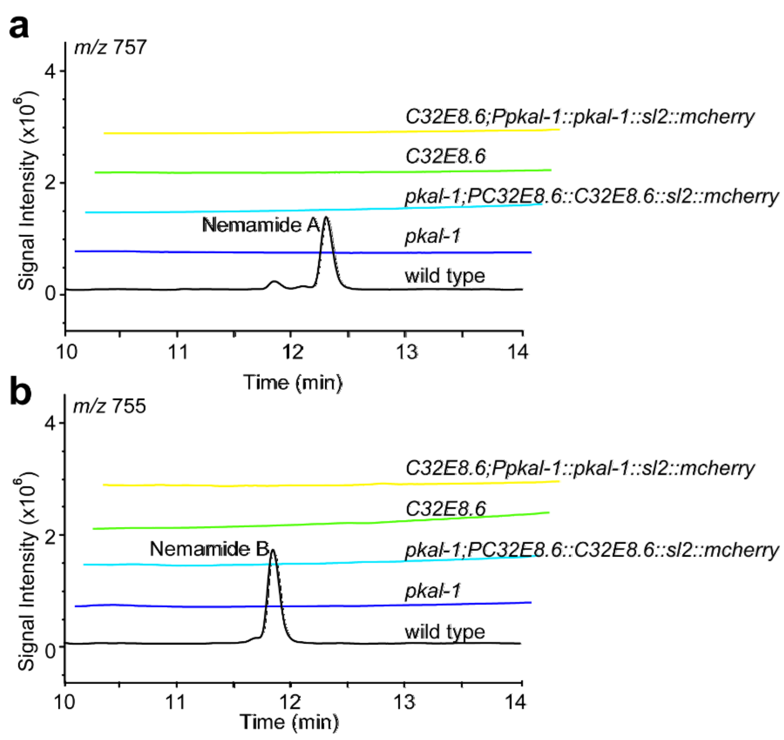

Supplementary Figure 11. Failure of T20F7.7 (*pkal-1*) and C32E8.6 to rescue each other. Extracted ion chromatogram for nemamide A (**a**) and nemamide B (**b**) in wild-type, the *pkal-1* mutant, the *pkal-1* mutant in which C32E8.6 was expressed under the control of its own promoter, the C32E8.6 mutant, and the C32E8.6 mutant in which *pkal-1* was expressed under the control of its own promoter. Note that the retention times of the nemamides in the Supplementary Information are different than in the main text due to the fact that the samples were analyzed on different columns.

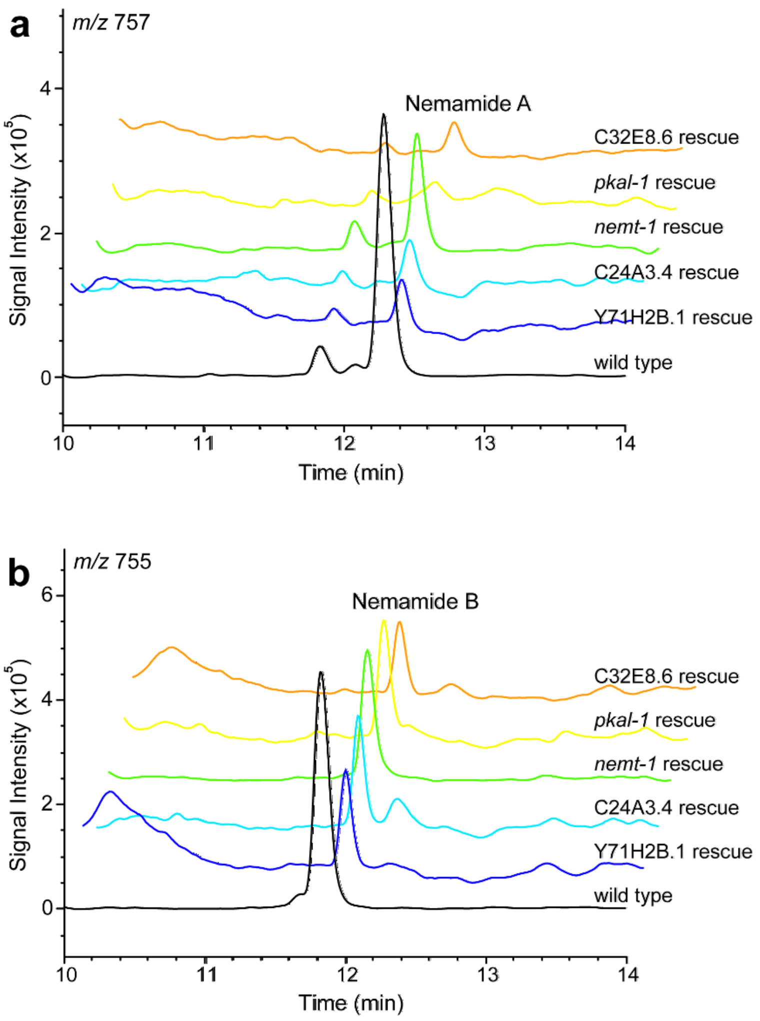

Supplementary Figure 12. Nemamide production in wild type and mutant rescue strains. Extracted ion chromatograms for nemamide A (**a**) and nemamide B (**b**). Mutants were rescued by complementing with *sl2::mCherry* plasmids under control of gene promoters::genes. Note that the retention times of the nemamides in the Supplementary Information are different than in the main text due to the fact that the samples were analyzed on different columns.

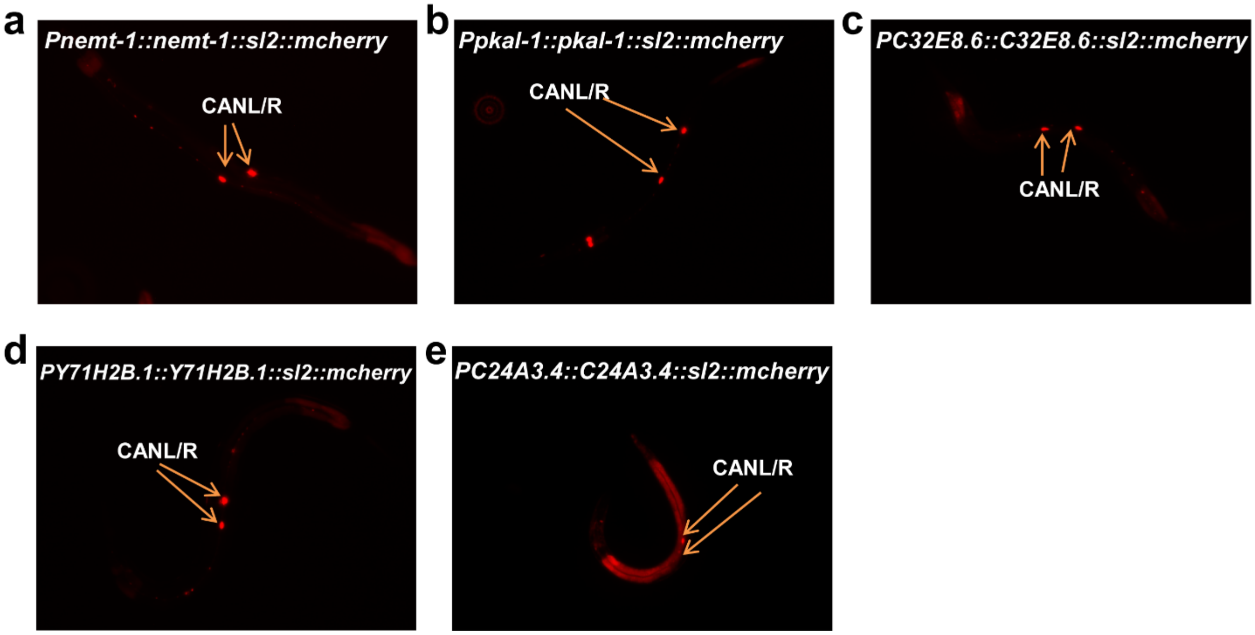

Supplementary Figure 13. Imaging of translational reporter strains. Analysis of in translational reporter worm strains *Pnemt-1::nemt-1::sl2::mcherry* (**a**), *Ppkal-1::pkal-1::sl2::mcherry* (**b**), *PC32E8.6::C32E8.6::sl2::mcherry* (**c**), *PY71H2B.1::Y71H2B.1::sl2::mcherry* (**d**), and *PC24A3.4::C24A3.4::sl2::mcherry* (**e**) demonstrated expression of nemamide biosynthetic genes primarily in the CANs.

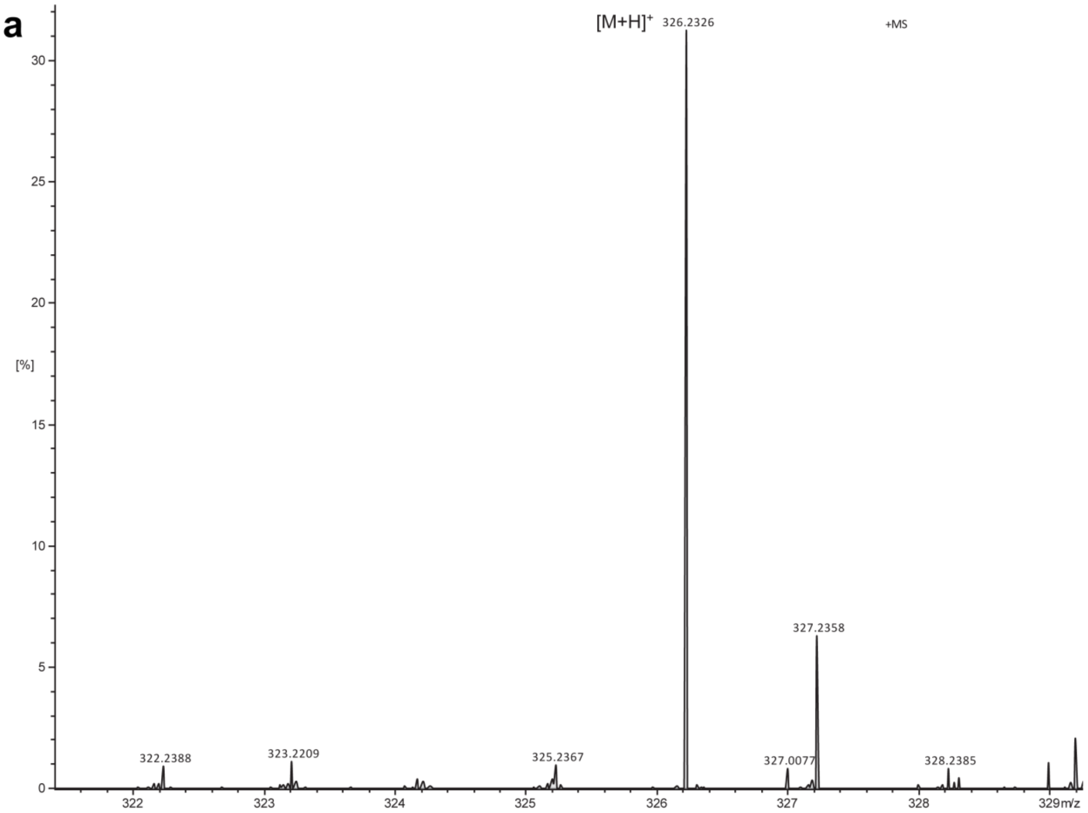
a
[M+H]+ 326.2326
+MS
327.2358
322.2388
323.2209
325.2367
327.0077
328.2385
[%]
m/z

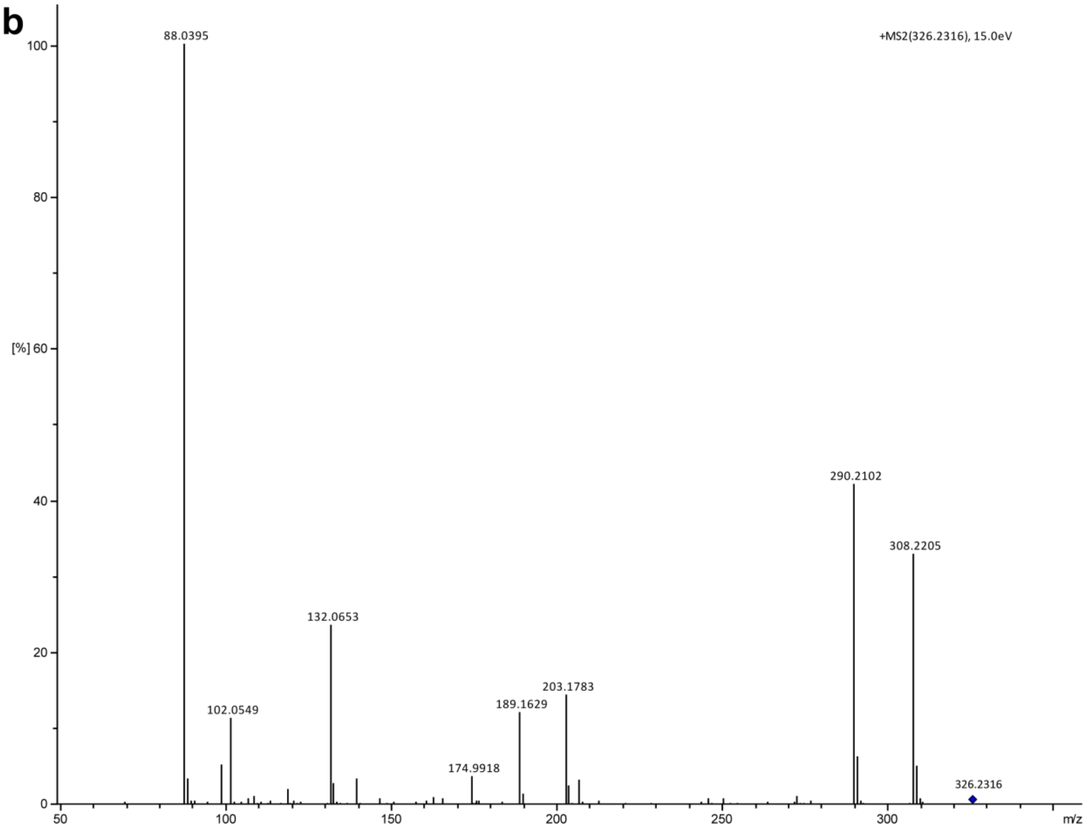
b
88.0395
+MS2(326.2316), 15.0eV
290.2102
308.2205
132.0653
102.0549
189.1629
203.1783
174.9918
326.2316
[%]
m/z

c

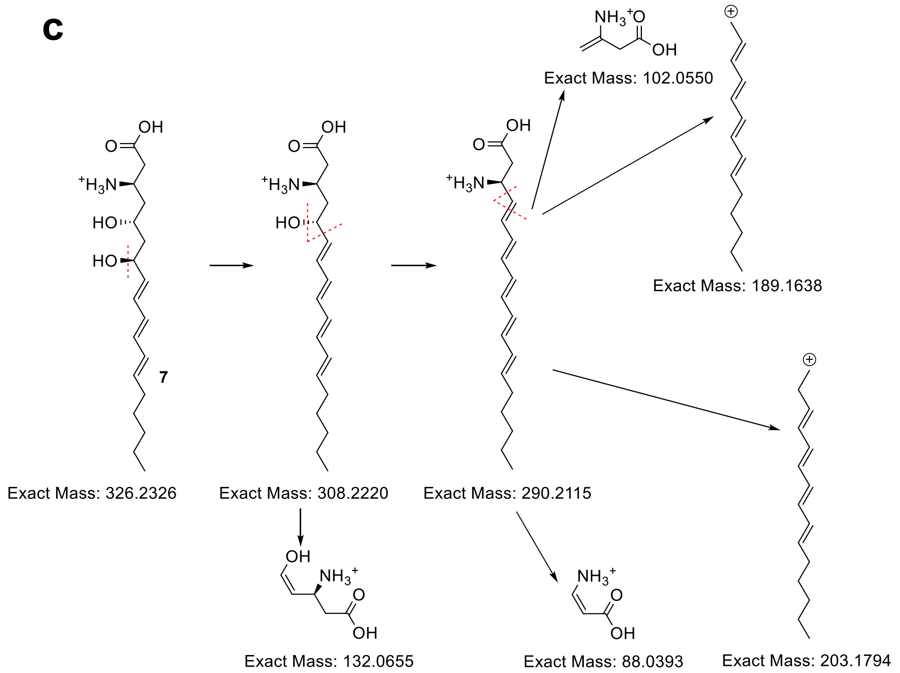

Supplementary Figure 14. Mass spectrometry analysis of intermediate 7. **a**,**b** HR-LC-MS/MS of intermediate **7**. **c** Analysis of the fragmentation patterns.

a

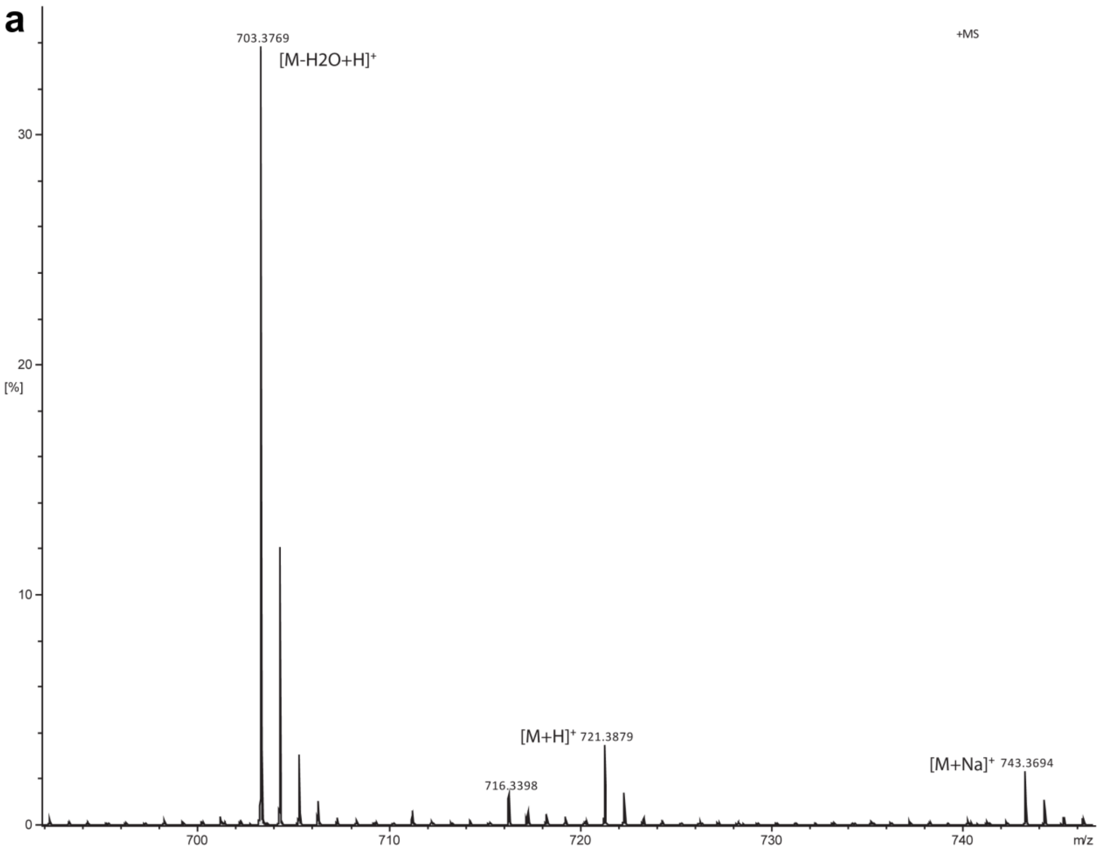
703.3769
[M-H2O+H]+
+MS
[%]
716.3398
[M+H]+ 721.3879
[M+Na]+ 743.3694
m/z

b

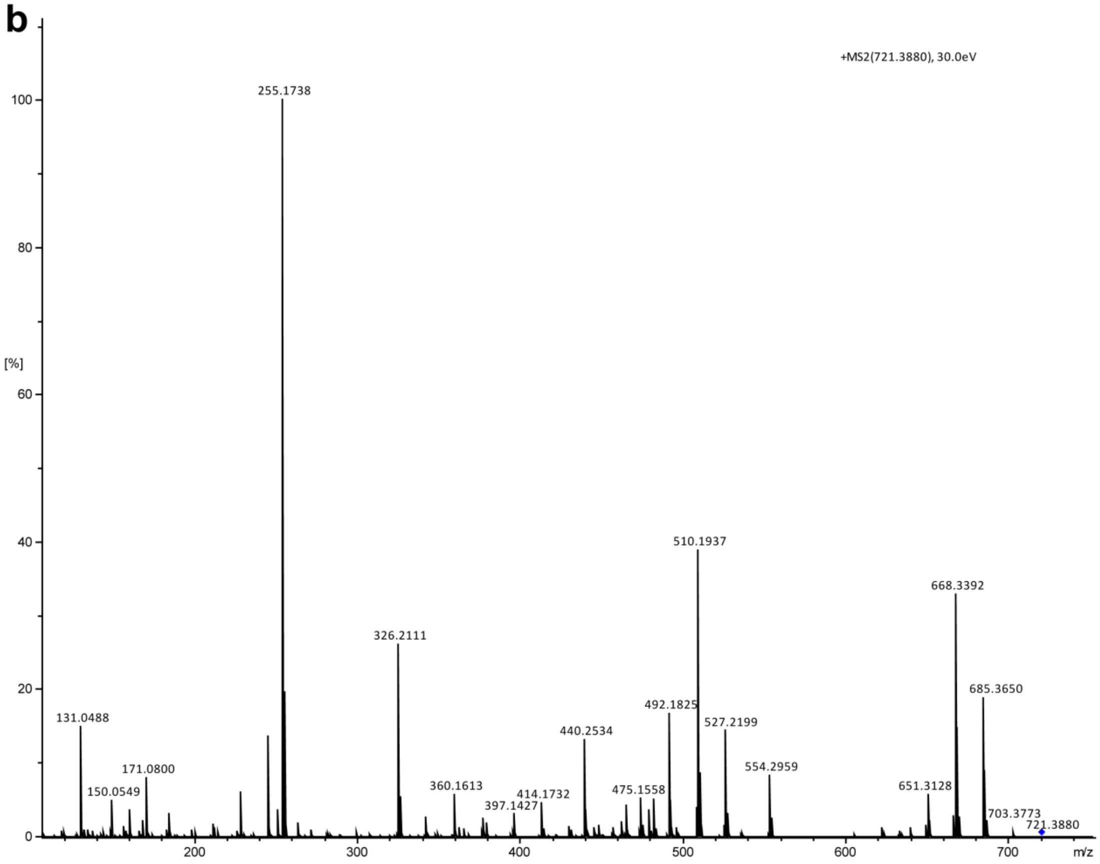
+MS2(721.3880), 30.0eV
255.1738
[%]
510.1937
668.3392
326.2111
685.3650
131.0488
492.1825
527.2199
440.2534
171.0800
554.2959
150.0549
360.1613
414.1732
475.1558
651.3128
397.1427
703.3773
721.3880
m/z

c

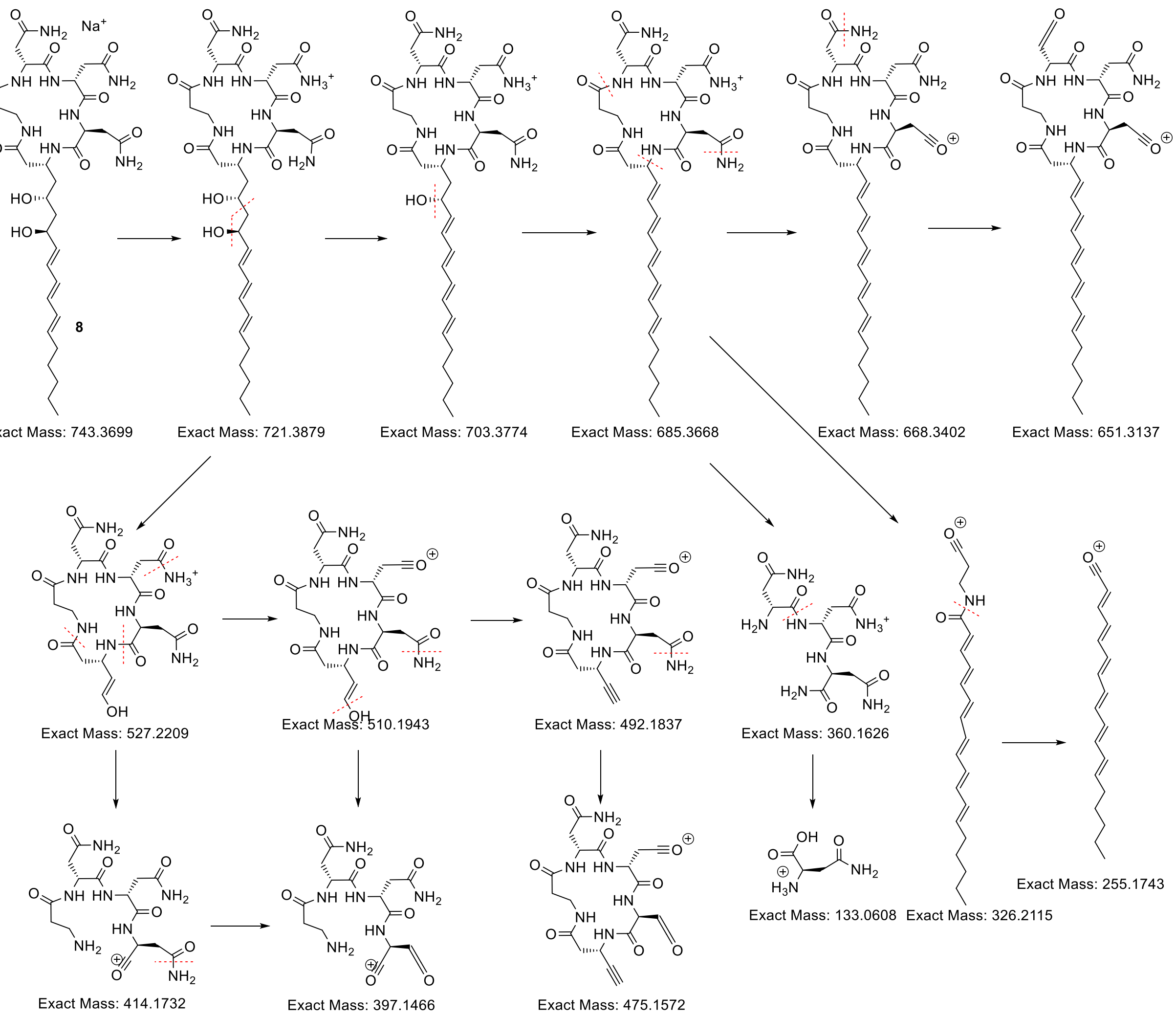

Supplementary Figure 15. Mass spectrometry analysis of desmethyl-nemamide, 8. a,b HR-LC-MS/MS of desmethyl-nemamide, **8**. **c** Analysis of the fragmentation patterns.

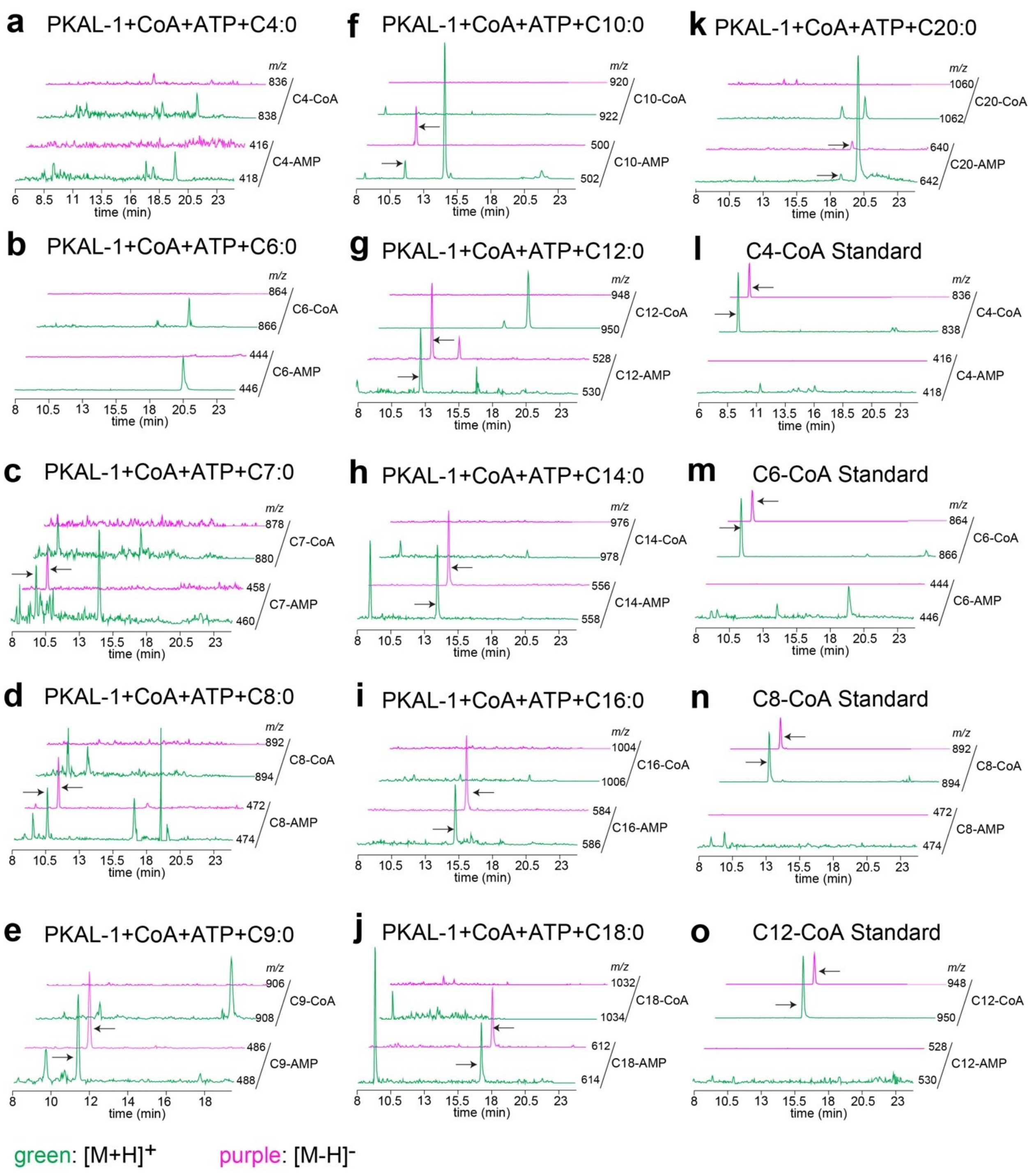

Supplementary Figure 16. PKAL-1 is not an FACL. Reaction of PKAL-1 with fatty acids of various lengths, ATP, and CoA did not result in the corresponding fatty acyl-CoAs. Several fatty acyl-CoAs were used as standards.

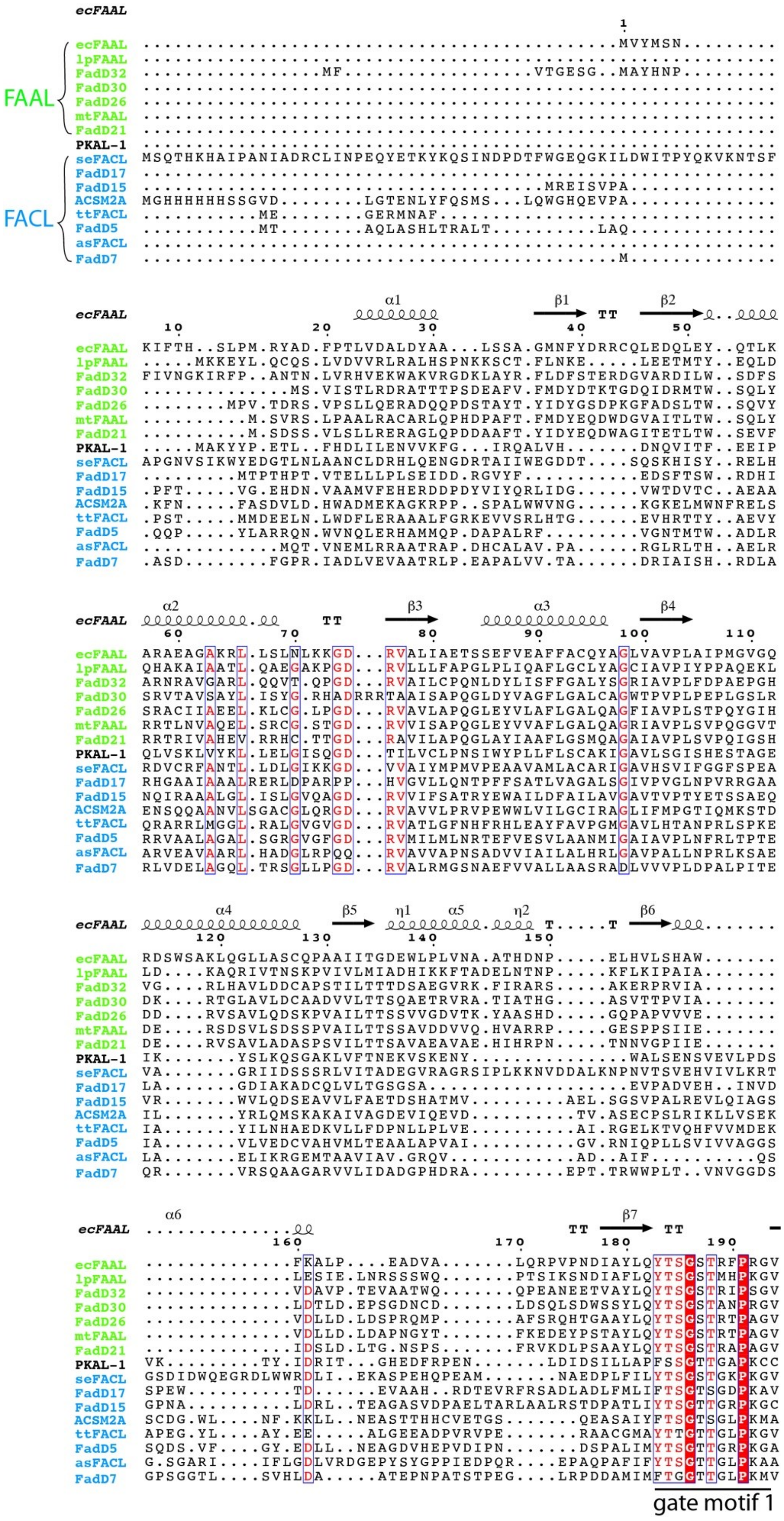
ecFAAL
1
ecFAAL ....MVYMSN.........
lpFAAL ....
FadD32 ...............MF.................VTGESG..MAYHNP.........
FadD30 ....
FadD26 ....
mtFAAL ....
FadD21 ....
PKAL-1 ....
seFACL MSQTHKHAIPANIADRCLINPEQYETKYKQSINDPDTFWGEQGKILDWITPYQKVKNTSF
FadD17 ....
FadD15 ....MREISVPA.............
ACSM2A MGHHHHHHSSGVD........LGTENLYFQSMS...LQWGHQEVPA.............
ttFACL ..........ME........GERMNAF.............................
FadD5 ..........MT........AQLASHLTRALT..........LAQ.............
asFACL ....
FadD7 ....M..............
FAAL
FACL
ecFAAL α1 β1 TT β2 10 20 30 40 50
ecFAAL KIFTH..SLPM.RYAD.FPTLVDALDYAA...LSSA.GMNFYDRRCQLEDQLEY..QTLK
lpFAAL .....MKKEYL.QCQS.LVDVVRLRALHSPNKKSCT.FLNKE.....LEETMTY..EQLD
FadD32 FIVNGKIRFP..ANTN.LVRHVEKWAKVRGDKLAYR.FLDFSTERDGVARDILW..SDFS
FadD30 .............MS.VISTLRDRATTTPSDEAFV.FMDYDTKTGDQIDRMTW..SQLY
FadD26 ........MPV.TDRS.VPSLLQERADQQPDSTAYT.YIDYGSDPKGFADSLTW..SQVY
mtFAAL .........M.SVRS.LPAALRACARLQPHDPAFT.FMDYEQDWDGVAITLTW..SQLY
FadD21 .........M.SDSS.VLSLLRERAGLQPDDAAFT.YIDYEQDWAGITETLTW..SEVF
PKAL-1 .....MAKYYP.ETL..FHDLILENVVKFG...IRQALVH.......DNQVITF..EEIP
seFACL APGNVSIKWYEDGTLNLAANCLDRHLQENGDRTAIIWEGDDT....SQSKHISY..RELH
FadD17 ........MTPTHPT.VTELLLPLSEIDD.RGVYF..........EDSFTSW..RDHI
FadD15 .PFT.....VG.EHDN.VAAMVFEHERDDPDYVIYQRLIDG......VWTDVTC..AEAA
ACSM2A .KFN.....FASDVLD.HWADMEKAGKRPP..SPALWWVNG......KGKELMWNFRELS
ttFACL .PST.....MMDEELN.LWDFLERAAALFGRKEVVSRLHTG......EVHRTTY..AEVY
FadD5 .QQP.....YLARRQN.WVNQLERHAMMQP.DAPALRF.........VGNTMTW..ADLR
asFACL ..........MQT.VNEMLRRAATRAP.DHCALAV.PA......RGLRLTH..AELR
FadD7 .ASD........FGPR.IADLVEVAATRLP.EAPALVV.TA......DRIAISH..RDLA
ecFAAL α2 TT β3 α3 β4 60 70 80 90 100 110
ecFAAL ARAEAGAKRL.LSLNLKKGD...RVALIAETSSEFVEAFFACQYAGLVAVPLAIPMGVGQ
lpFAAL QHAKAIAATL.QAEGAKPGD...RVLLLFAPGLPLIQAFLGCLYAGCIAVPIYPPAQEKL
FadD32 ARNRAVGARL.QQVT.QPGD...RVAILCPQNLDYLISFFGALYSGRIAVPLFDPAEPGH
FadD30 SRVTAVSAYL.ISYG.RHADRRRTAAISAPQGLDYVAGFLGALCAGWTPVPLPEPLGSLR
FadD26 SRACIIAEEL.KLCG.LPGD...RVAVLAPQGLEYVLAFLGALQAGFIAVPLSTPQYGIH
mtFAAL RRTLNVAQEL.SRCG.STGD...RVVISAPQGLEYVVAFLGALQAGRIAVPLSVPQGGVT
FadD21 RRTRIVAHEV.RRHC.TTGD...RAVILAPQGLAYIAAFLGSMQAGAIAVPLSVPQIGSH
PKAL-1 QLVSKLVYKL.LEL GISQGD...TILVCLPNSIWYPLLFLSCAKIGAVLSGISHESTAGE
seFACL RDVCRFANTL.LDLGIKKGD...VVAIYMPMVPEAAVAMLACARIGAVHSVIFGGFSPEA
FadD17 RHGAAIAAALRERLDPARPP...HVGVLLQNTPFFSATLVAGALSGIVPVGLNPVRRGAA
FadD15 NQIRAAALGL.ISLGVQAGD...RVVIFSATRYEWAILDFAILAVGAVTVPTYETSSAEQ
ACSM2A ENSQQAANVLSGACGLQRGD...RVAVVLPRVPEWWLVILGCIRAGLIFMPGTIQMKSTD
ttFACL QRARRLMGGL.RALGVGVGD...RVATLGFNHFRHLEAYFAVPGMGAVLHTANPRLSPKE
FadD5 RRVAALAGAL.SGRGVGFGD...RVMILMLNRTEFVESVLAANMIGAIAVPLNFRLTPTE
asFACL ARVEAVAARL.HADGLRPQQ...RVAVVAPNSADVVIAILALHRLGAVPALLNPRLKSAE
FadD7 RLVDELAGQL.TRSGLLPGD...RVALRMGSNAEFVVALLAASRADLVVVPLDPALPITE
ecFAAL α4 β5 η1 α5 η2 T.....T β6 120 130 140 150
ecFAAL RDSWSAKLQGLLASCQPAAIITGDEWLPLVNA.ATHDNP.....ELHVLSHAW.......
lpFAAL LD....KAQRIVTNSKPVIVLMIADHIKKFTADELNTNP.....KFLKIPAIA.......
FadD32 VG....RLHAVLDDCAPSTILTTTDSAEGVRK.FIRARS.....AKERPRVIA.......
FadD30 DK....RTGLAVLDCAADVVLTTSQAETRVRA.TIATHG.....ASVTTPVIA.......
FadD26 DD....RVSAVLQDSKPVAILTTSSVVGDVTK.YAASHD.....GQPAPVVVE.......
mtFAAL DE....RSDSVLSDSSPVAILTTSSAVDDVVQ.HVARRP.....GESPPSIIE.......
FadD21 DE....RVSAVLADASPSVILTTSAVAEAVAE.HIHRPN.....TNNVGPIIE.......
PKAL-1 IK.......YSLKQSGAKLVFTNEKVSKENY................WALSENSVEVLPDS
seFACL VA.......GRIIDSSSRLVITADEGVRAGRSIPLKKNVDDALKNPNVTSVEHVIVLKRT
FadD17 LA.......GDIAKADCQLVLTGSGSA..................EVPADVEH..INVD
FadD15 VR.......WVLQDSEAVVLFAETDSHATMV.........AEL.SGSVPALREVLQIAGS
ACSM2A IL.......YRLQMSKAKAIVAGDEVIQEVD.........TV.ASECPSLRIKLLVSEK
ttFACL IA.......YILNHAEDKVLLFDPNLLPLVE..........AI.RGELKTVQHFVVMDEK
FadD5 IA.......VLVEDCVAHVMLTEAALAPVAI..........GV.RNIQPLLSVIVVAGGS
asFACL LA.......ELIKRGEMTAAVIAV.GRQV............AD..AIF..........QS
FadD7 QR.......VRSQAAGARVVLIDADGPHDRA.........EPT.TRWWPLT..VNVGGDS
ecFAAL α6 TT β7 TT 160 170 180 190
ecFAAL ..............FKALP....EADVA.......LQRPVPNDIAYLQYTSGSTRFPRGV
lpFAAL ..............LESIE.LNRSSSWQ.......PTSIKSNDIAFLQYTSGSTMHPKGV
FadD32 ..............VDAVP.TEVAATWQ.......QPEANEETVAYLQYTSGSTRIPSGV
FadD30 ..............LDTLD.EPSGDNCD.......LDSQLSDWSSYLQYTSGSTANPRGV
FadD26 ..............VDLLD.LDSPRQMP.......AFSRQHTGAAYLQYTSGSTRTPAGV
mtFAAL ..............VDLLD.LDAPNGYT.......FKEDEYPSTAYLQYTSGSTRTPAGV
FadD21 ..............IDSLD.LTG.NSPS.......FRVKDLPSAAYLQYTSGSTRAPAGV
PKAL-1 VK.........TY.IDRIT...GHEDFRPEN.......LDIDSILLAPFSSGTTGAPKCC
seFACL GSDIDWQEGRDLWWRDLI..EKASPEHQPEAM.......NAEDPLFILYTSGSTGKPKGV
FadD17 SPEW..........TD......EVAAH..RDTEVRFRSADLADLFMLIFTSGTSGDPKAV
FadD15 GPNA..........LDRL..TEAGASVDPAELTARLAALRSTDPATLIYTSGTTGRPKGC
ACSM2A SCDG.WL....NF.KKLL..NEASTTHHCVETGS.......QEASAIYFTSGTSGLPKMA
ttFACL APEG.YL....AY.EE......ALGEEADPVRVPE.......RAACGMAYTTGTTGLPKGV
FadD5 SQDS.VF....GY.EDLL..NEAGDVHEPVDIPN.......DSPALIMYTSGTTGRPKGA
asFACL G.SGARI....IFLGDLVRDGEPYSYGPPIEDPQR....EPAQPAFIFYTSGTTGLPKAA
FadD7 GPSGGTL....SVHLDA.....ATEPNPATSTPEG....LRPDDAMIMFTGGTTGLPKMV
gate motif 1

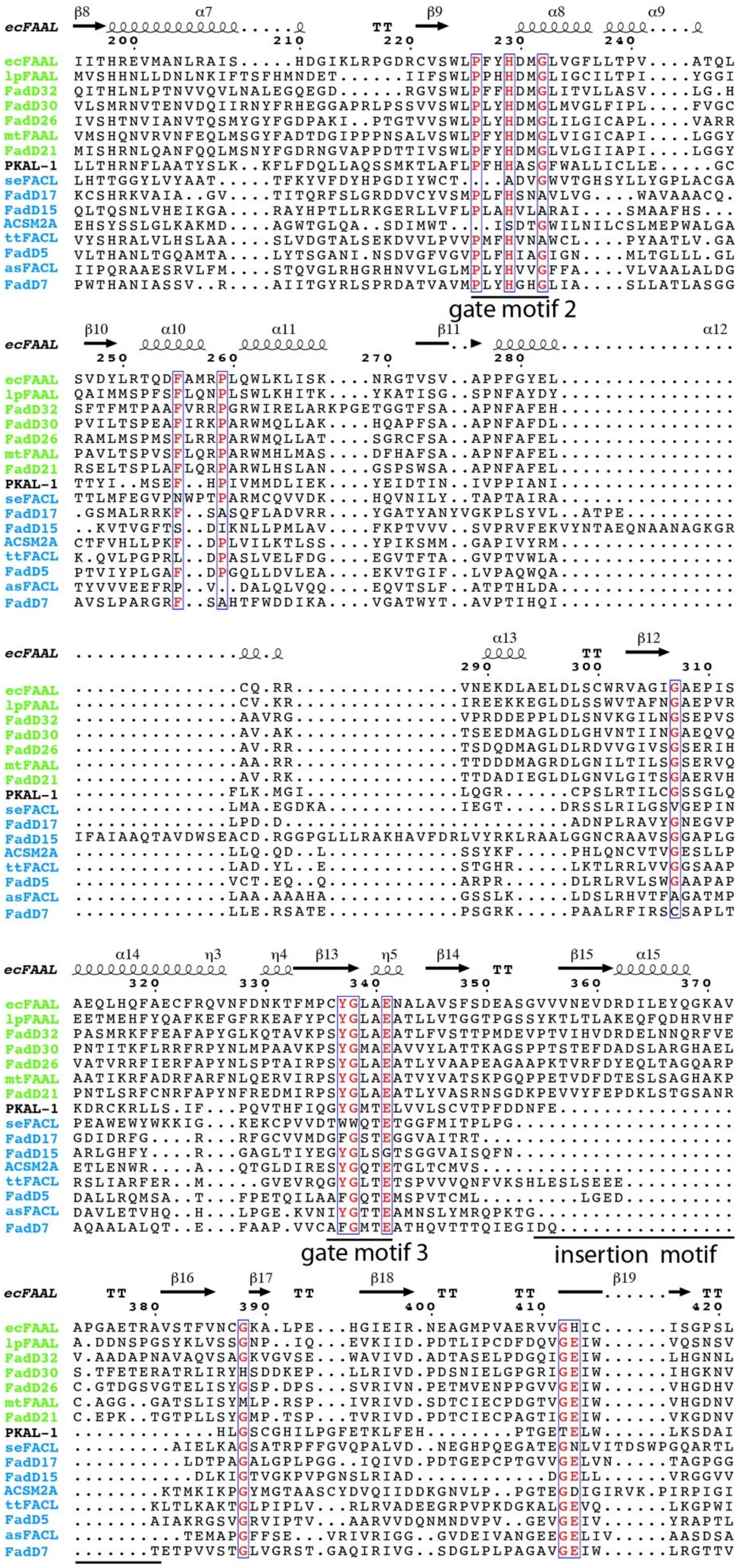

β8 α7 β9 α8 α9
ecFAAL TT 200 210 220 230 240
ecFAAL IITHREVMANLRAIS.....HDGIKLRPGDRCVSWLPFYHDMGLVGFLLTPV....ATQL
lpFAAL MVSHHNLLDNLNKIFTSFHMNDET......IIFSWLPPHHDMGLIGCILTPI....YGGI
FadD32 QITHLNLPTNVVQVLNALEGQEGD......RGVSWLPFFHDMGLITVVLLASV....LG.H
FadD30 VLSMRNVTENVDQIIRNYFRHEGGAPRLPSSVVSWLPLYHDMGLMVGLFIPL....FVGC
FadD26 IVSHTNVIANVTQSMYGYFGDPAKI..PTGTVVSWLPLYHDMGLILGICAPL....VARR
mtFAAL VMSHQNVRVNFEQLMSGYFADTDGIPPPNSALVSWLPFYHDMGLVIGICAPI....LGGY
FadD21 MISHRNLQANFQQLMSNYFGDRNGVAPPDTTIVSWLPFYHDMGLVLGIIAPI....LGGY
PKAL-1 LLTHRNFLAATYSLK..KFLFDQLLAQSSMKTLAFLPFHHASGFWALLICLLE.....GC
seFACL LHTTGGYLVYAAT.....TFKYVFDYHPGDIYWCT....ADVGWVTGHSYLLYGPLACGA
FadD17 KCSHRKVAIA..GV....TITQRFSLGRDDVCYVSMPLFHSNAVLVG....WAVAAACQ.
FadD15 QLTQSNLVHEIKGA....RAYHPTLLRKGERLLVFLPLAHVLARAI....SMAAFHS...
ACSM2A EHSYSSLGLKAKMD....AGWTGLQA..SDIMWT....ISDTGWILNILCSLMEPWALGA
ttFACL VYSHRALVLHSLAA....SLVDGTALSEKDVVLPVVPMFHVNAWCL....PYAATLV.GA
FadD5 VLTHANLTGQAMTA....LYTSGANI.NSDVGFVGVPLFHIAGIGN....MLTGLLL.GL
asFACL IIPQRAAESRVLFM....STQVGLRHGRHNVVLGLMPLYHVVGFFA....VLVAALALDG
FadD7 PWTHANIASSV..R....AIITGYRLSPRDATVAVMPLYHGHGLIA....SLLATLASGG
gate motif 2
β10 α10 α11 β11 α12
ecFAAL 250 260 270 280
ecFAAL SVDYLRTQDFAMRPLQWLKLISK....NRGTVSV..APPFGYEL................
lpFAAL QAIMMSPFSFLQNPLSWLKHITK....YKATISG..SPNFAYDY................
FadD32 SFTFMTPAAFVRRPGRWIRELARKPGETGGTFSA..APNFAFEH................
FadD30 PVILTSPEAFIRKPARWMQLLAK....HQAPFSA..APNFAFDL................
FadD26 RAMLMSPMSFLRRPARWMQLLAT....SGRCFSA..APNFAFEL................
mtFAAL PAVLTSPVSFLQRPARWMHLMAS....DFHAFSA..APNFAFEL................
FadD21 RSELTSPLAFLQRPARWLHSLAN....GSPSWSA..APNFAFEL................
PKAL-1 TTYI..MSEF..HPIVMMDLIEK....YEIDTIN..IVPPIANI................
seFACL TTLMFEGVPNWPTPARMCQVVDK....HQVNILY..TAPTAIRA................
FadD17 .GSMALRRKF..SASQFLADVRR....YGATYANYVGKPLSYVL..ATPE..........
FadD15 ..KVTVGFTS..DIKNLLPMLAV....FKPTVVV..SVPRVFEKVYNTAEQNAANAGKGR
ACSM2A CTFVHLLPKF..DPLVILKTLSS....YPIKSMM..GAPIVYRM................
ttFACL K.QVLPGPRL..DPASLVELFDG....EGVTFTA..GVPTVWLA................
FadD5 PTVIYPLGAF..DPGQLLDVLEA....EKVTGIF..LVPAQWQA................
asFACL TYVVVEEFRP..V..DALQLVQQ....EQVTSLF..ATPTHLDA................
FadD7 AVSLPARGRF..SAHTFWDDIKA....VGATWYT..AVPTIHQI................
α13 TT β12
ecFAAL 290 300 310
ecFAAL ...............CQ.RR..............VNEKDLAELDLSCWRVAGIGAEPIS
lpFAAL ...............CV.KR..............IREEKKEGLDLSSWVTAFNGAEPVR
FadD32 ....AAVRG..............VPRDDEPPLDLSNVKGILNGSEPVS
FadD30 ..............AV.AK..............TSEEDMAGLDLGHVNTIINGAEQVQ
FadD26 ..............AV.RR..............TSDQDMAGLDLRDVVGIVSGSERIH
mtFAAL ..............AA.RR..............TTDDDMAGRDLGNILTILSGSERVQ
FadD21 ..............AV.RK..............TTDADIEGLDLGNVLGITSGAERVH
PKAL-1 ..............FLK.MGI.............LQGR......CPSLRTILCGSSGLQ
seFACL ..............LMA.EGDKA...........IEGT.....DRSSLRILGSVGEPIN
FadD17 ..............LPD.D.......................ADNPLRAVYGNEGVP
FadD15 IFAIAAQTAVDWSEACD.RGGPGLLLRAKHAVFDRLVYRKLRAALGGNCRAAVSGGAPLG
ACSM2A ..............LLQ.QD..L...........SSYKF.....PHLQNCVTVGESLLP
ttFACL ..............LAD.YL..E...........STGHR.....LKTLRRLVVGGSAAP
FadD5 ..............VCT.EQ..Q...........ARPR......DLRLRVLSWGAAPAP
asFACL ..............LAA.AAAHA...........GSSLK.....LDSLRHVTFAGATMP
FadD7 ..............LLE.RSATE...........PSGRK.....PAALRFIRSCSAPLT
α14 η3 η4 β13 η5 β14 TT β15 α15
ecFAAL 320 330 340 350 360 370
ecFAAL AEQLHQFAECFRQVNFDNKTFMPCYGLAENALAVSFSDEASGVVVNEVDRDILEYQGKAV
lpFAAL EETMEHFYQAFKEFGFRKEAFYPCYGLAEATLLVTGGTPGSSYKTLTLAKEQFQDHRVHF
FadD32 PASMRKFFEAFAPYGLKQTAVKPSYGLAEATLFVSTTPMDEVPTVIHVDRDELNNQRFVE
FadD30 PNTITKFLRRFRPYNLMPAAVKPSYGMAEAVVYLATTKAGSPPTSTEFDADSLARGHAEL
FadD26 VATVRRFIERFAPYNLSPTAIRPSYGLAEATLYVAAPEAGAAPKTVRFDYEQLTAGQARP
mtFAAL AATIKRFADRFARFNLQERVIRPSYGLAEATVYVATSKPGQPPETVDFDTESLSAGHAKP
FadD21 PNTLSRFCNRFAPYNFREDMIRPSYGLAEATLYVASRNSGDKPEVVYFEPDKLSTGSANR
PKAL-1 KDRCKRLLS.IF...PQVTHFIQGYGMTELVVLSCVTPFDDNFE................
seFACL PEAWEWYWKKIG...KEKCPVVDTWWQTETGGFMITPLPG....................
FadD17 GDIDRFG....R...RFGCVVMDGFGSTEGGVAITRT.......................
FadD15 ARLGHFY....R...GAGLTIYEGYGLSGTSGGVAISQFN....................
ACSM2A ETLENWR....A...QTGLDIRESYGQTETGLTCMVS.......................
ttFACL RSLIARFER..M.....GVEVRQGYGLTETSPVVVQNFVKSHLESLSEEE..........
FadD5 DALLRQMSA..T...FPETQILAAFGQTEMSPVTCML.........LGED..........
asFACL DAVLETVHQ..H...LPGE.KVNIYGTTEAMNSLYMRQPKTG..................
FadD7 AQAALALQT..E...FAAP.VVCAFGMTEATHQVTTTQIEGIDQ................
gate motif 3
insertion motif
TT β16 β17 TT β18 TT TT β19 TT
ecFAAL 380 390 400 410 420
ecFAAL APGAETRAVSTFVNCGKA.LPE...HGIEIR.NEAGMPVAERVVGHIC......ISGPSL
lpFAAL A.DDNSPGSYKLVSSGNP..IQ...EVKIID.PDTLIPCDFDQVGEIW......VQSNSV
FadD32 V.AADAPNAVAQVSAGKVGVSE...WAVIVD.ADTASELPDGQIGEIW......LHGNNL
FadD30 S.TFETERATRLIRYHSDDKEP...LLRIVD.PDSNIELGPGRIGEIW......IHGKNV
FadD26 C.GTDGSVGTELISYGSP.DPS...SVRIVN.PETMVENPPGVVGEIW......VHGDHV
mtFAAL C.AGG..GATSLISYMLP.RSP...IVRIVD.SDTCIECPDGTVGEIW......VHGDNV
FadD21 C.EPK..TGTPLLSYGMP.TSP...TVRIVD.PDTCIECPAGTIGEIW......VKGDNV
PKAL-1 .............HLGSCGHILPGFETKLFEH........PTGETELW......LKSDAI
seFACL .........AIELKAGSATRPFFGVQPALVD..NEGHPQEGATEGNLVITDSWPGQARTL
FadD17 ..........LDTPAGALGPLPGG..IQIVD.PDTGEPCPTGVVGELVN.....TAGPGG
FadD15 ...........DLKIGTVGKPVPGNSLRIAD...........DGELL......VRGGVV
ACSM2A ........KTMKIKPGYMGTAASCYDVQIIDDKGNVLP..PGTEGDIGIRVK.PIRPIGI
ttFACL .......KLTLKAKTGLPIPLV...RLRVADEEGRPVPKDGKALGEVQ......LKGPWI
FadD5 .......AIAKRGSVGRVIPTV...AARVVDQNMNDVPV..GEVGEIV......YRAPTL
asFACL ..........TEMAPGFFSE...VRIVRIGG..GVDEIVANGEEGELIV.....AASDSA
FadD7 .......TETPVVSTGLVGRST.GAQIRIVG..SDGLPLPAGAVGEIW......LRGTTV

```
                        α16                                  β20          β21
ecFAAL                                                  ....►TT
            430                     440              450          460

ecFAAL  MSGYFGDQVS...................QDEI.AATGWLDTGDLGYL.LDGYLYVTGRI
lpFAAL  AKGYWNQPEE.TRHAFAGKIKDD.........E.RSAIYLRTGDLGFL.HENELYVTGRI
FadD32  GTGYWGKEEE.SAQTFKNILKSRISESRAEGAP.DDALWVRTGDYGTY.FKDHLYIAGRI
FadD30  STGYHNADDALNRDKFQASIREA.....SAGT.PRSPWLRTGDLGFI.VGDEFYIVGRM
FadD26  TMGYWQKPKQ.TAQVFDAKLVDP......APAA.PEGPWLRTGDLGVI.SDGELFIMGRI
mtFAAL  ANGYWQKPDE.SERTFGGKIVTP.....SPGT.PEGPWLRTGDSGFV.TDGKMFIIGRI
FadD21  AEGYWNKPDE.TRHTFGAMLVHP.....SAGT.PDGSWLRTGDLGFL.SEDEMFIVGRM
PKAL-1  MKAYKNGTP.......................NLDEDDWLHTGDIV.TEKGGFFYVVDRM
seFACL  FGDHERFEQT...................YF.S.TFKNMYFSGDGARRDEDGYYWITGRV
FadD17  FEGYYNDEAA...................EAER.MAGGVYHSGDLAYRDDAGYAYFAGRL
FadD15  FSGYWRNEQA...................TTEA.FTDGWFKTGDLGAVDEDGFLTITGRK
ACSM2A  FSGYVDNPDK...................TAAN.IRGDFWLLGDRGIKDEDGYFQFMGRA
ttFACL  TGGYYGNEEA...................TRSALTPDGFFRTGDIAVWDEEGYVEIKDRL
FadD5   MSCYWNNPEA...................TAEA.FAGGWFHSGDLVRMDSDGYVWVVDRK
asFACL  FVGYLNQPQA...................TAEK.LQDGWYRTSDVAVWTPEGTVRILGRV
FadD7   VRGYLGDPTI...................TAAN.FTDGWLRTGDLGSLSAAGDLSIRGRI

           β22  β23    α17             β24                    β25
ecFAAL     .►TT                  TT          TT          ................
                470      480          490       500

ecFAAL  KDLII.IRGRNIWPQDIEYIAEQEPEIH.SGDAIAFVTAQEKII................
lpFAAL  KDLII.IYGKNHYPQDIEFSLMHSPLHHVLGKCAAFVIQEEHEY................
FadD32  KDLVI.IDGRNHYPQDLECTAQESTKALRVGYAAAFSVPANQLPQTVFDDSHAGLKFDPE
FadD30  KDLII.QDGVNHYPDDIETTVKEFTG....GRVAAFSVSDDG.V................
FadD26  KDLLI.VDGRNHYPDDIEATIQEITG....GRAAAIAVPDDI.T................
mtFAAL  KDLLI.VYGRNHSPDDIEATIQEITR....GRCAAISVPGDRST................
FadD21  KDMLI.VYGRNHYPEDIESTVQEITG....GRVAAISVPVDH.T................
PKAL-1  KDLIK.LNGYQVSPTEIENVILTLPK...VAEVAVVGIEDELCG................
seFACL  DDVLN.VSGHRLGTAEIESALVAHPK...IAEAAVVGIPHAIKG................
FadD17  GDWMR.VDGENLGTAPIERVLMRYPD...ATEVAVYPVPDPVVG................
FadD15  KEIIVTAGGKNVAPAVLEDQLRAHPL...ISQAVVVGDAKPFIG................
ACSM2A  DDIIN.SSGYRIGPSEVENALMEHPA...VVETAVISSPDPVRG................
ttFACL  KDLIK.SGGEWISSVDLENALMGHPK...VKEAAVVAIPHPKWQ................
FadD5   KDMII.SGGENIYCAELENVLASHPD...IAEVAVIGRADEKWG................
asFACL  DDMII.SGGENIHPSEIERVLGTAPG...VTEVVVIGLADQRWG................
FadD7   KELIN.RGGEKISPERVEGVLASHPN...VMEAAVFGVPHQLYG................
        hinge region

                                                α18
ecFAAL  .....                        ....                      .....
                 510                        520             530

ecFAAL  .....LQIQCRIS....D..........E....ERRGQLIHALAAR.....IQSEFGV.T
lpFAAL  ....KLTVMCEVKNRFMD..........D....VAQDNLFNEIFEL.....VYENHQLEV
FadD32  DTSEQLVIVGER.....A..........AGTHKLDHQPIVDDIRAA.....IAVGHGVTV
FadD30  ...EHLVIAAEVRTEHGP..........DKVTIMDFSTIKRLVVSA.....LSKLHGLHV
FadD26  ...EQLVAIIEFKRRGST..........AEEVMLKLRSVKREVTSA.....ISKSHSLRV
mtFAAL  ...EKLVAIIELKKRGDS..........DQDAMARLGAIKREVTSA.....LSSSHGLSV
FadD21  ...EKLVTVIELKLLGDS..........AGEAMDELDVIKNNVTAA.....ISRSHGLNV
PKAL-1  ....QLPKAYIVLEKNADE.................LLFLKHLEHTMKEKL........S
seFACL  ....QAIYAYVTLNHGEEP...............SPELYA.EVRNWVRK.........EI
FadD17  ....DQVMAALVLAPGTKF...................DADKFRAFLTE.......QPDL
FadD15  ....ALITIDPEAFEGWKQRNSKTAGASVGDLATDPDLIA.EIDAAVKQANLAVSHAESI
ACSM2A  ....EVVKAFVV..............LASQFLSHDPEQLTKELQQH.VKSV........T
ttFACL  ....ERPLAVVV..............PRGEKP......TPEELNEHLLKAG........F
FadD5   ....EVPIAVAA..............VTNDDL......RIEDLGE.FLTDR........L
asFACL  ....QSVTACVV..............PRLGET.....LSADALDTFCRSSE........L
FadD7   ....EAVAAVIV..............PRESAP.....PTREELVQFCRER.........L

              β26                     α19
ecFAAL      ....      TT      TT
                  540       550       560       570

ecFAAL  AA....IDLLPPHSIPRTSSGKPARAEAKKRYQKAYAASLNVQESLA.............
lpFAAL  HT....IVLIPLKAMPHTTSGKIRRNFCRKHLLDKTLPIVATWQLNKI...EE.......
FadD32  RD....VLLVSAGTIPRTSSGKIGRRACRAAYLDGSLRSGVGSPTVFA...TS...D...
FadD30  TD....FLLVPPGALPKTTSGKISRAACAKQYGANKLQRVATFP................
FadD26  AD....LVLVSPGSIPITTSGKIRRSACVERYRSDGFKRLDVAV................
mtFAAL  AD....LVLVAPGSIPITTSGKVRRGACVEQYRQDQFARLDA..................
FadD21  AD....LVLVPPGSIPTTTSGKIRRAACVEQYRLQQFTRLDG..................
PKAL-1  AVKQLRGGVSIIKEMPKSSSGKIQKNRLMYY.............................
seFACL  GPLATPDVLHWTDSLPKTRSGKIMRRILRKIAAGDTSNLGDTSTLADPGVVEKLLEEKQA
FadD17  GHKQWPSYVRVSAGLPRTMTFKVIKRQLSAEGVACADPVWP...IRR.............
FadD15  RKFRILPVDFTEDTGELTPTMKVKRKVVAEKFASDIEAIYN...KE..............
ACSM2A  APYKYPRKIEFVLNLPKTVTGKIQRAKLRDKE............................
ttFACL  AKWQLPDAYVFAEEIPRTSAGKFLKRALREQYKNYYGGA.....................
FadD5   ARYKHPKALEIVDALPRNPAGKVLKTELRLRYGACVNVERR...SASAGFTERRE.NRQK
asFACL  ADFKRPKRYFILDQLPKNALNKVLRRQLVQQVSS..........................
FadD7   AAFEIPASFQEASGLPHTAKGSLDRRAVAERFGHSV........................
                          highly conserved
                          loop region in
                          C-domain
ecFAAL

ecFAAL  .....
lpFAAL  .....
FadD32  .....
FadD30  .....
FadD26  .....
mtFAAL  .....
FadD21  .....
PKAL-1  .....
seFACL  IAMPS
FadD17  .....
FadD15  .....
ACSM2A  .....
ttFACL  .....
FadD5   L....
asFACL  .....
FadD7   .....
```

Supplementary Figure 17. Sequence alignment of PKAL-1 with FAAL enzymes. The FAAL enzymes (in green) are ecFAAL from *Escherichia coli*, lpFAAL from *Legionella pneumophila*, mtFAAL from *Mycobacterium tuberculosis*, and FadD21, FadD26, FadD30, and FadD32 from *M. tuberculosis*, and FACL enzymes (in blue), including seFACL from *Salmonella enterica*, ttFACL from *Thermus thermophilus*, asFACL from *Alcaligenes sp.*, ACSM2A from human, and FadD5, FadD7, FadD15, and FadD17 from *M. tuberculosis*. The conserved motifs, including the insertion motif that is present in the FAAL enzymes, but missing in PKAL-1 and FACL enzymes, are indicated.

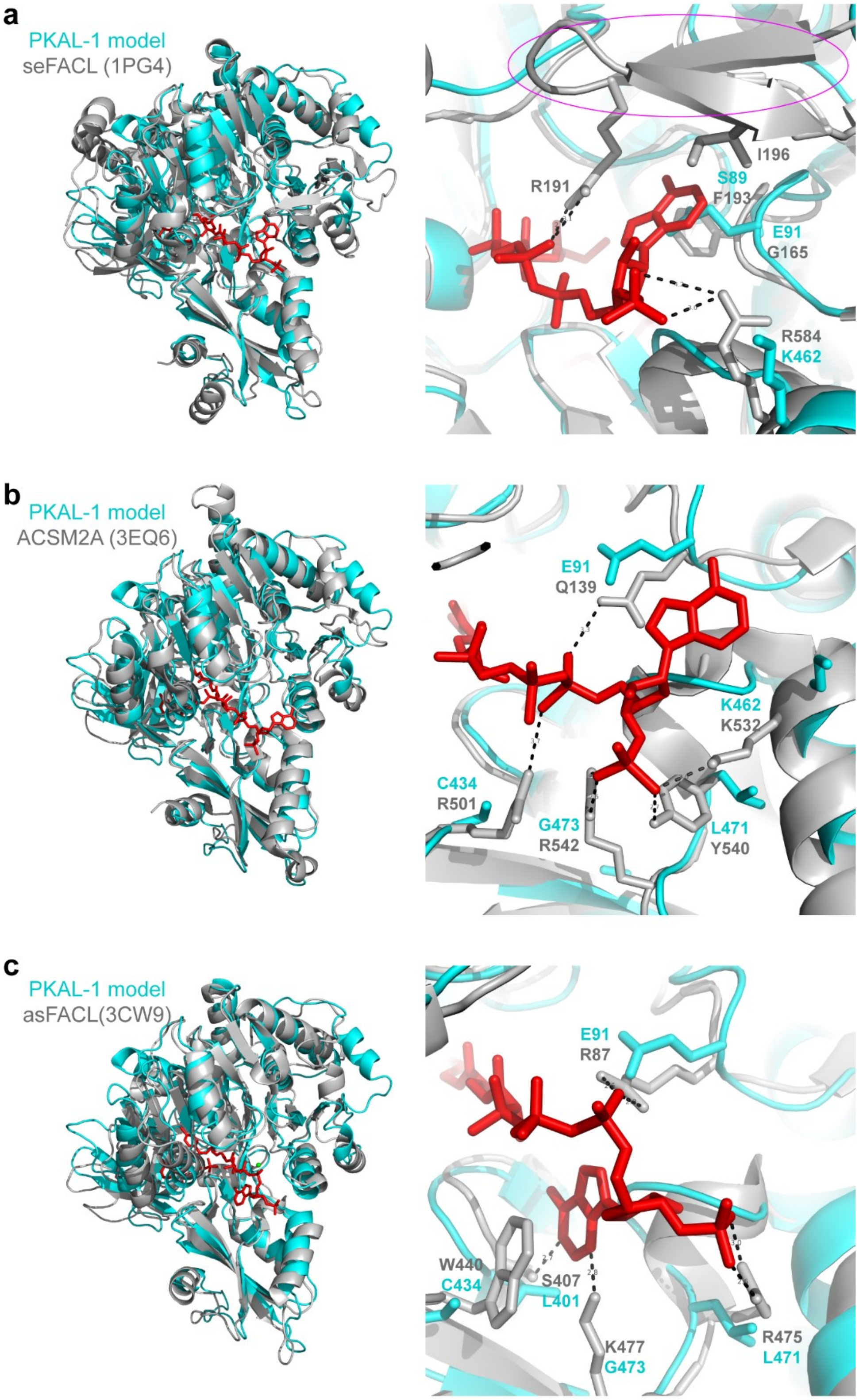

Supplementary Figure 18. Comparison of the modeled PKAL-1 structure to the structures of FACL enzymes bound to CoA substrates. PKAL-1 was modeled using Phyre2[4], and six

templates 5GXD, 6EQO, 6PLJ, 5ES8, 6MFZ, and 5U89 were selected for modeling, enabling 100% of residues to be modeled at >90% confidence. Using Pymol 2.3.3, the PKAL-1 structural model was then overlaid with the structures of three FACL enzymes in the thioester-forming conformation: (**a**) 1PG4, the structure of acetyl-CoA synthetase from *Salmonella enterica* (seFACL), bound to adenosine-5'-propylphosphate and CoA (in red), (**b**) 3EQ6, the structure of the human medium-chain acyl-CoA synthetase (ACSM2A) bound to AMP and butyryl-CoA (in red), and (**c**) 3CW9, the structure of 4-chlorobenzoate:CoA ligase from *Alcaligenes sp.* (asFACL) bound to 4-chlorophenyacyl-CoA (in red). In (**a**), the seFACL structure has a beta hairpin (circled in pink), which contains R191 that binds CoA and which is missing in the PKAL-1 model. A hydrophobic pocket for the adenine ring of CoA that is formed by I196 in the beta hairpin and F193 and G165 is also missing in the PKAL-1 model. In (**b**), the ACSM2A structure has several residues, including R501, R542, Y540, and Q139, which are important for binding of the CoA substrate and which are replaced with C434, G473, L471, and E91, respectively, in the PKAL-1 model. In (**c**), the asFACL structure has several residues, including W440, S407, K477, R475, and R87, which are important for binding of the CoA substrate and which are replaced with C434, L401, G473, L471, and E91, respectively, in the PKAL-1 model.

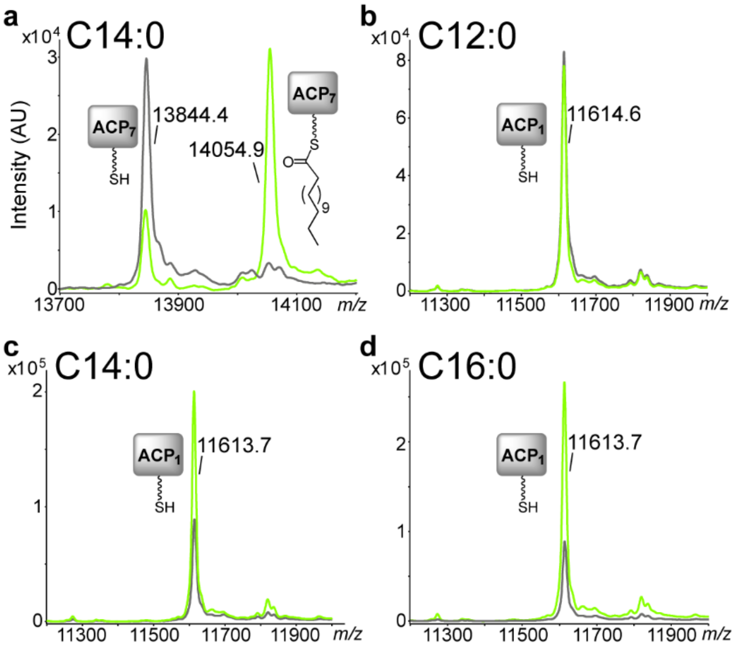

Supplementary Figure 19. Loading of PKS-1_ACP$_1$ or NRPS-1_ACP$_7$ by PKAL-1. To determine the carrier protein specificity of PKAL-1, PKAL-1 was incubated with (**a**) holo-ACP$_7$ (positive control), ATP, and C14:0 fatty acid, or with (**b**-**d**) holo-ACP$_1$ from PKS-1, ATP, and C12:0 fatty acid (**b**), C14:0 fatty acid (**c**), or C16:0 fatty acid (**d**). Samples were analyzed by MALDI to determine if the fatty acid substrates were loaded onto the respective carrier proteins.

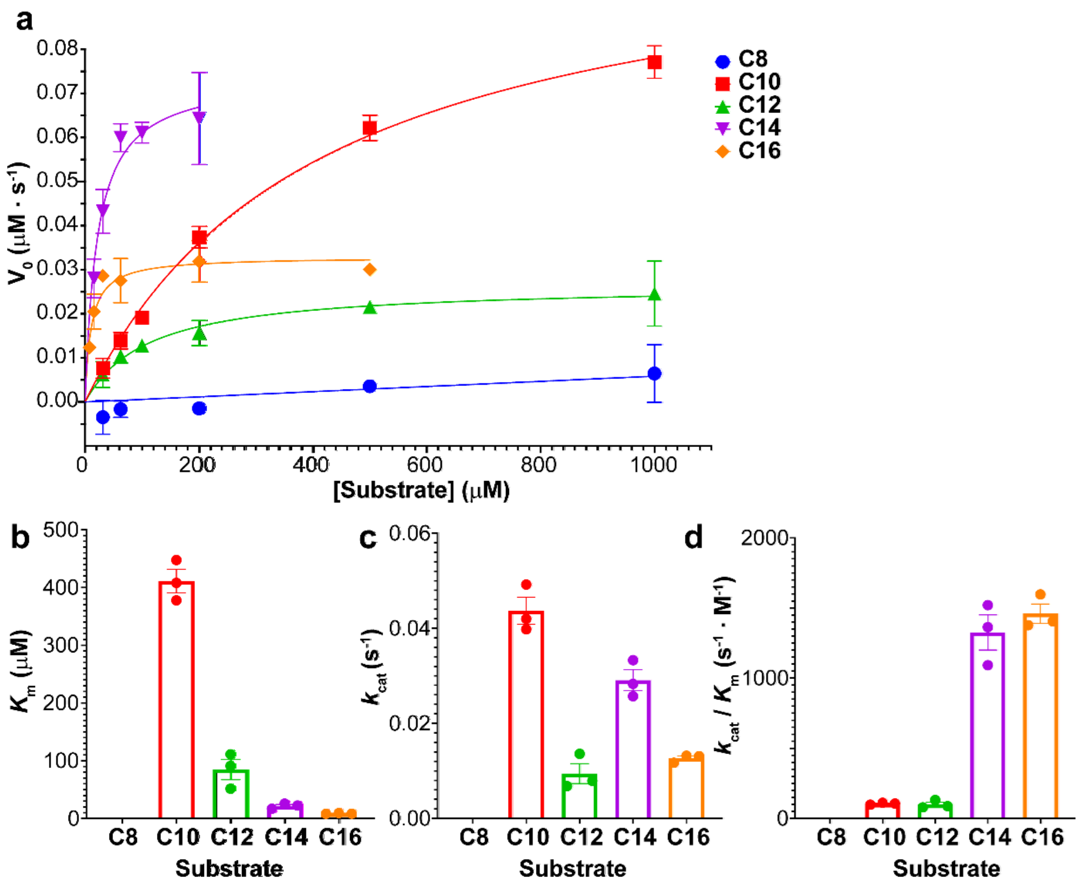

Supplementary Figure 20. Kinetic data for PKAL-1 against various fatty acid substrates. **a** PKAL-1 was analyzed in an enzyme-coupled continuous kinetic assay using C8:0, C10:0, C12:0, C14:0, and C16:0 fatty acids as substrates. Kinetic constants, including K_m (**b**), k_{cat} (**c**), and k_{cat} / K_m (**d**), were obtained using GraphPad software. Data represent the mean ± SEM of three independent experiments. Source data are provided as a Source Data file.

Supplementary Table 1. A domain selectivity codes for the PKS-1 A_1 domain from various nematode species.

Species	235	236	239	278	299	301	322	330	331	517
C. elegans	D	V	S	F	T	G	I	I	W	K
C. angaria	D	V	S	F	T	G	I	I	W	K
C. japonica	D	V	A	F	T	G	I	V	W	K
C. brenneri	D	V	S	F	T	G	I	I	W	K
C. remanei	D	V	S	F	T	G	I	V	W	K
C. briggsae	D	V	S	F	T	G	I	V	W	K
C. tropicalis	D	V	S	F	T	G	I	I	W	K
A. suum	D	V	M	Y	F	G	I	I	W	K
T. canis	D	V	M	F	F	G	I	I	W	K
D. immitis	D	V	M	F	Y	G	I	I	W	K
O. volvulus	D	V	V	F	Y	G	I	V	W	K
L. loa	D	V	V	F	Y	G	I	V	W	K
B. malayi	D	V	M	F	F	G	I	I	W	K
P. pacificus	D	V	F	F	I	G	I	I	W	K
P. exspectatus	D	V	F	F	I	G	I	I	W	K
S. carpocapsae	D	V	F	F	Y	G	I	I	W	K
B. xylophilus	D	V	F	F	I	G	I	I	W	K
A. ceylanicum	D	V	M	F	F	G	I	V	W	K
A. duodenale	D	V	M	F	L	G	I	I	W	K
O. dentatum	D	V	L	F	F	G	I	V	W	K
N. americanus	D	V	F	F	V	G	I	V	W	K
H. bacteriophora	D	V	V	F	F	G	I	V	W	K
H. contortus	D	V	F	F	F	G	I	V	W	K

Supplementary Table 2. Genes with enriched expression (> 5-fold) in the CANs.

Enriched genes	Predicted function from WormBase	Initial strain used for screening	Defective in nemamide production?
C24A3.4	Predicted to have CoA-transferase activity; homolog of human AMACR (alpha-methylacyl-CoA racemase)	VC40591	Yes
srv-1	Serpentine receptor, class V		
T22F3.12	Predicted to have peptidyl-prolyl cis-trans isomerase activity; ortholog of PPIAL4A (peptidylprolyl isomerase A like 4A); PPIAL4D (peptidylprolyl isomerase A like 4D); and PPIL6 (peptidylprolyl isomerase like 6)		
F49C12.10 (*nemt-1*)	Predicted to have methyltransferase activity	VC20249	Yes
dhhc-5	Predicted to have protein-cysteine S-palmitoyltransferase activity; homolog of human ZDHHC21 (zinc finger DHHC-type palmitoyltransferase 21)		
cyk-1	Predicted to have Rho GTPase binding activity and actin binding activity		
C32E8.6	Contains AMP-dependent synthetase domain	No strain available (RAB60, RAB61 generated)	Yes
T05A1.5	Ortholog of human SLC22A9 (solute carrier family 22 member 9)		
pks-1	Polyketide synthase	N/A	Yes
C08G5.6	No homology to known protein domains		
C03F11.4	No homology to known protein domains; located in the genome near *pks-1*	VC40279	No
ZK112.6	Weak homology to acetyl-CoA synthetase		
Y46H3C.7	No homology to known protein domains		
C41A3.2	No homology to known protein domains; enriched in coelomocyte, germ line, head mesodermal cell, and sensory neurons; located in the genome near *pks-1*		
aexr-1	Predicted to have neuropeptide receptor activity		
R04A9.6	No homology to known protein domains; enriched in AVE and hypodermis		
acy-2	Predicted to have adenylate cyclase activity; involved in adenylate cyclase-modulating G protein-coupled receptor signaling pathway and nematode larval development		
C23H5.11	No homology to known protein domains		
F27C8.2	Contains an acyl-CoA N-acyltransferases domain; enriched in GABAergic neurons, body wall muscle cell, excretory cell, and seam cell		
pqn-52	Contains prion-like (Q/N-rich) domain; enriched in cholinergic neurons, coelomocyte, germ line, germline precursor cell, and head mesodermal cell		

pak-1	Member of P21-activated kinase family; exhibits GTP binding activity and protein kinase activity; involved in hemidesmosome assembly, inductive cell migration, and motor neuron axon guidance		
T12G3.4	Ortholog of human APMAP (adipocyte plasma membrane-associated protein)		
Y54E5A.2	No homology to known protein domains; enriched in males; involved in spermatogenesis		
nrps-1	Nonribosomal peptide synthetase	N/A	Yes
F27C8.3	No homology to known protein domains		
ace-3	Exhibits acetylcholinesterase activity; involved in hatching and regulation of backward locomotion; expressed in body wall musculature, neurons, pharyngeal muscle cell, and vulva		
ZK112.5	No homology to known protein domains; enriched in coelomocyte, male-specific tissues, and pharynx		
F13H8.9	Ortholog of human SCLY (selenocysteine lyase)	RB2336	No
lips-13	Predicted to have lipase activity		
F09C12.6	Predicted to have G-protein coupled receptor activity		
moc-1	Predicted to have molybdopterin adenylyltransferase activity and molybdopterin molybdotransferase activity; expressed in tail; ortholog of human GPHN (gephyrin)		
F42C5.6	Contains SUP-1-like domain; expressed in AVE		
K10C2.12	Contains ubiquitin-like domain; expressed in AVG, RIM, and command interneuron		
acbp-6	Predicted to have fatty-acyl-CoA-binding activity		
acs-9 *(pkal-1)*	Member of fatty acyl-CoA synthetase family; predicted to have catalytic activity	VC40189	Yes
swt-1	Exhibits sugar transmembrane transporter activity; localizes to golgi and plasma membrane; ortholog of human SLC50A1 (solute carrier family 50 member 1)		
Y71H2B.1	Predicted to have fatty-acyl-CoA binding activity	VC40597	Yes
F40E3.5	Predicted to have protein serine/threonine phosphatase activity	VC40998	No

Genes were identified using the dataset of Cao *et al.* and GExplore, applying an enrichment ratio of 5-fold in the CANs and a false detection rate of 0.05.[5,6] Enrichment ratio reflects the ratio of gene expression in the cell type where the gene is most highly expressed versus gene expression in the cell type where the gene is next most highly expressed. Genes were selected for screening for involvement in nemamide production based on the putative enzymatic role of the encoded protein and the availability of a corresponding loss-of-function mutant. C03F11.4 was selected based on its proximity to *pks-1* in the genome.

Supplementary Table 3. Strains used in this study.

Strain	Genotype	Mutation	Background	Resource
N2	wild type			CGC
VC40591	C24A3.4(*gk961371*) X	Large deletion		CGC
VC20249	F49C12.10(*gk208727*) IV	R176Opal		CGC
VC40279	C03F11.4(*gk551362*) X	R144Opal		CGC
RB2336	F13H8.9(*ok3172*) II	754bp deletion with 28 bp insertion		CGC
VC40597	Y71H2B.1*(gk712674)* III	W136Opal		CGC
VC40998	F40E3.5(gk930567) I	Q47Ochre		CGC
VC40189	*acs-9(gk504580) X*	Q436Amber		CGC
RAB43	*nrps-1(gk186409[C_4_S1934N]; gk186410[C_4_D1971N])* III	Located in NRPS-1_C_4	VC20469 (4X outcrossing)	CGC
RAB45	Y71H2B.1*(gk712674)* III	W136Opal	VC40597(2X outcrossing)	CGC
RAB51	*nrps-1(reb8[ACP_7_S307V])* III	NRPS-1_ACP_7	N2	CRISPR-Cas9
RAB52	*pks-1(reb9[C_1_H6685A])* X	PKS-1_C_1	N2	CRISPR-Cas9
RAB53	*nrps-1(reb10[C_3_H1486A])* III	NRPS-1_C_3	N2	CRISPR-Cas9
RAB54	*pks-1(reb11[TE_1_S7593A])* X	PKS-1_TE_1	N2	CRISPR-Cas9
RAB55	*nrps-1(reb12[TE_2_S2803A])* III	NRPS-1_TE_2	N2	CRISPR-Cas9
RAB56	*pks-1(reb13[TE_1_S7593C]; reb14[TE_1_G7596A])* X	PKS-1_TE_1	N2	CRISPR-Cas9
RAB57	*nemt-1(reb15)* IV	306 bp deletion with 27 bp insertion	N2	CRISPR-Cas9
RAB58	*pkal-1(reb21)* X	154 bp deletion with single base 'T' insertion	N2	CRISPR-Cas9
RAB59	*pkal-1(reb28)* X	552 bp deletion	N2	CRISPR-Cas9
RAB60	C32E8.6*(reb23)* I	991 bp deletion with 10 bp insertion	N2	CRISPR-Cas9
RAB61	C32E8.6*(reb24)* I	993 bp deletion	N2	CRISPR-Cas9
RAB62	C24A3.4*(reb16)* X	1361 bp deletion	N2	CRISPR-Cas9
RAB67	*pkal-1(reb28)*; *pks-1(reb11[TE_1_S7593A])*			Outcrossing
RAB68	*pkal-1(reb28)*; *nemt-1(reb15)*			Outcrossing
RAB69	*nemt-1(reb15); pks-1(reb11[TE_1_ S7593A])*			Outcrossing
RAB72	*rebEx15 (Pnemt-1::gfp, 50 ng/µL; CAN::mcherry, 50 ng/µL)*		N2	Transgenesis
RAB73	*rebEx16 (Ppkal-1::gfp, 50 ng/µL; CAN::mcherry, 50 ng/µL)*		N2	Transgenesis
RAB74	*rebEx17 (PC32E8.6::gfp, 50 ng/µL; CAN::mcherry, 50 ng/µL)*		N2	Transgenesis

RAB76	*nemt-1(reb15); rebEx19(Pnemt-1::nemt-1::sl2::mcherry, 50 ng/µL)*			Transgenesis
RAB77	*pkal-1(reb28); rebEx20(Ppkal-1::pkal-1::sl2::mcherry, 50 ng/µL)*			Transgenesis
RAB78	*C32E8.6(reb24); rebEx21(PC32E8.6::C32E8.6::sl2::mcherry, 50 ng/µL)*			Transgenesis
RAB79	*C32E8.6(reb24); rebEx20(Ppkal-1::pkal-1::sl2::mcherry, 50 ng/µL)*			Transgenesis
RAB80	*pkal-1(reb28); rebEx21(PC32E8.6::C32E8.6::sl2::mcherry, 50 ng/µL)*			Transgenesis
RAB81	Y71H2B.1*(gk712674); rebEx22(P*Y71H2B.1*::*Y71H2B.1*::sl2::mcherry, 50 ng/µL)*			Transgenesis
RAB82	C24A3.4(*reb16); rebEx23(PC24A3.4::C24A3.4::sl2::mcherry, 50 ng/µL)*			Transgenesis
RAB89	*pks-1(reb22[A_1_G7106E])* X	PKS-1_A_1	N2	CRISPR-Cas9
RAB103	*pks-1(reb29[PCP_2_S7463A])* X	PKS-1_PCP_2	N2	CRISPR-Cas9
RAB109	*nrps-1(reb31[A_3_ G2337D])* III	NRPS-1_A_3	N2	CRISPR-Cas9
RAB110	*nrps-1(reb32[A_2_ G964D])* III	NRPS-1_A_2	N2	CRISPR-Cas9

Supplementary Table 4. Single worm PCR primers for mutant strains in this study.

Strain	Genotype	Sequence
RAB43	*nrps-1 (gk186409[C_4_S1934N]; gk186410[C_4_D1971N])* III	Forward: CTGAAGCCTTTATTCAGTGCCAAG<br>Reverse: CTTGCACTGCTAGAGCTAAGCTTC
RAB45	Y71H2B.1*(gk712674)* III	Forward: GGAAAGCACGGAGATTTTGAAG<br>Reverse: AGTGATGGGAATGGTCTCTGTT
RAB51	*nrps-1(reb8[ACP_7_S307V])* III	Forward: GAAGGAGCAGCAAACATCGAGAA<br>Reverse: ATCTGAGTGACCTGCTTTCAGAG
RAB52	*pks-1(reb9[C_1_H6685A])* X	Forward: CATCTGTAAACCCTGCAGATATTGC<br>Reverse: CGGCATCGCAGAAAACTGATAATGC
RAB53	*nrps-1(reb10[C_3_H1486A])* III	Forward: GAAGCTGGTGGAGTTGTCCAATGCT<br>Reverse: GAAACTGTATCCCAGTTCTCTGGAG
RAB54	*pks-1(reb11[TE_1_S7593A])* X	Forward: GGTGATTAAATCTGGAGTAC<br>Reverse: TAGTCCAGAGAAGACGTACT
RAB55	*nrps-1(reb12[TE_2_S2803A])* III	Forward: TCGAGACCAAACTCGGAATC<br>Reverse: TCTGAGAAAATGTTCACCGG
RAB56	*pks-1(reb13[TE_1_S7593C]; reb14[TE_1_G7596A])* X	Forward: GAGGTGATTAAATCTGGAGTACGGC<br>Reverse: TCACTATCCGGTAGTCCAGAGAAG
RAB57	*nemt-1(reb15)* IV	Forward: AGTGGCTTTGCCTTTCCTCCTT<br>Reverse: AGCCCTCAACTACTTCATCAGTG
RAB58	*pkal-1(reb21)* X	Forward: GAGCTCGGGATTTCTCAAGGT<br>Reverse: CAATTCTGCAACACAGAATGTCG
RAB59	*pkal-1(reb28)* X	Forward: GAGCTCGGGATTTCTCAAGGT<br>Reverse: CAATTCTGCAACACAGAATGTCG
RAB60	*C32E8.6(reb23)* I	Forward: GCTTCAACTCCAGAGAATCAGG<br>Reverse: CAACGGCTCTCCGCTCTTAAG
RAB61	*C32E8.6 (reb24)* I	Forward: GCTTCAACTCCAGAGAATCAGG<br>Reverse: CAACGGCTCTCCGCTCTTAAG
RAB62	C24A3.4*(reb16)* X	Forward: CTCTGCCGTACCAGTGATGTTCTA<br>Reverse: CTATCCATGTGCTACCAAACTTGTC
RAB89	*pks-1(reb22[A_1_G7106E])* X	Forward: CACCACTATACCAATTCGAAGAACTG<br>Reverse: AGTGACTTGTCAACTTTCCCACTTG
RAB103	*pks-1(reb29[PCP_2_S7463A])* X	Forward: GAGACTCACTGAGCAATGAAACTTG<br>Reverse: TCCAGATTTAATCACCTCTTCAGC
RAB109	*nrps-1(reb31[A_3_G2337D])* III	Forward: CTACCAGCAATTCTTTACTGCTAATTC<br>Reverse: CTTCTCAATTCTACAGACATCTCCA
RAB110	*nrps-1(reb32[A_2_G964D])* III	Forward: CCGTATCTCAAATCATAGGCC<br>Reverse: CCTATGTCCTCGCACCTTCACCTG

Supplementary Table 5. Single-worm PCR information used to confirm genotype of wild-type and mutant worm strains used in this study.

Strain	Genotype	Wild type	Mutant	Enzyme digestion
RAB43	*nrps-1 (gk186409[C_4_S1934N]; gk186410[C_4_D1971N])* III	445 bp	445 bp	Wild type is cut by BtsIMutI to 265bp + 180bp; no cut for mutant
RAB45	Y71H2B.1*(gk712674)* III	700 bp	700 bp	Wild type is cut by HinfI into 450bp + 200bp + 50bp; mutant is cut by HinfI into 500bp + 200bp
RAB51	*nrps-1(reb8[ACP_7_S307V])* III	820 bp	820 bp	Mutant is cut by AatII into 438bp + 382bp
RAB52	*pks-1(reb9[C_1_H6685A])* X	1098 bp	1098 bp	Mutant is cut by SphI into 750bp + 348bp
RAB53	*nrps-1(reb10[C_3_H1486A])* III	993 bp	993 bp	Mutant is cut by AseI into 658bp + 335bp
RAB54	*pks-1(reb11[TE_1_S7593A])* X	562 bp	562 bp	Mutant is cut by SphI into 355bp + 207bp
RAB55	*nrps-1(reb12[TE_2_S2803A])* III	539 bp	539 bp	Mutant is cut by NheI into 318bp + 221bp
RAB56	*pks-1(reb13[TE_1_S7593C]; reb14[TE_1_G7596A])* X	574 bp	574 bp	Mutant is cut by KasI into 364bp + 210bp
RAB57	*nemt-1(reb15)* IV	981 bp	702 bp	
RAB58	*pkal-1(reb21)* X	1723 bp	1570 bp	
RAB59	*pkal-1(reb28)* X	1723 bp	1171 bp	
RAB60	*C32E8.6(reb23)* I	1405 bp	424 bp	
RAB61	*C32E8.6(reb24)* I	1405 bp	412 bp	
RAB62	C24A3.4*(reb16)* X	1863 bp	502 bp	
RAB89	*pks-1(reb22[A_1_G7106E])* X	1350 bp	1350 bp	Mutant is cut by SalI into 914bp + 436bp
RAB103	*pks-1(reb29[PCP_2_S7463A])* X	1057bp	1057bp	Mutant is cut by SalI into 914bp + 436bp
RAB109	*nrps-1(reb31[A_3_ G2337D])* III	1009bp	1009bp	Wild type cut by BspEI to 395bp + 614bp (mutant not cut)
RAB110	*nrps-1(reb32[A_2_ G964D])* III	1062bp	1062bp	Mutant cut by SalI to 380bp + 682bp

Supplementary Table 6. gRNA sequences used for CRISPR-Cas9 in this study.

Strain	Genotype (alleles)	DNA encoding sgRNA (20 bases+NGG, and vector for cloning) or crRNA
RAB51	*nrps-1(reb8[ACP$_7$_S307V])* III	CTCCAGCTCGGCGAGTCTTA AGG (pTM55-FE*)
RAB52	*pks-1(reb9[C$_1$_H6685A])* X	ATCATATTTTAACTGATGGT TGG (pTM55-FE*)
RAB53	*nrps-1(reb10[C$_3$_H1486A])* III	GGCTTCTACCATCGCAGATC AGG (pTM55-FE*)
RAB54	*pks-1(reb11[TE$_1$_S7593A])* X	TTCGTTATGGGGCACTCGAT GGG (pTM55)
		CTTCGTTATGGGGCACTCGA TGG (pTM55)
		GTTATGGGGCACTCGATGGG TGG (pTM55)
RAB55	*nrps-1(reb12[TE$_2$_S2803A])* III	ACCTCTAAATTGGTGTTCAT TGG (pTM55)
		TTCATTGGCGCCTCGTCTGC TGG (pTM55)
RAB56	*pks-1(reb13[TE$_1$_S7593C];*	TTCGTTATGGGGCACTCGAT GGG (pTM55)
	reb14[TE$_1$_G7596A]) X	CTTCGTTATGGGGCACTCGA TGG (pTM55)
		GTTATGGGGCACTCGATGGG TGG (pTM55)
RAB57	*nemt-1(reb15)* IV	TATTACTACAGTTATGGCTT TGG (pTM55-FE*)
		CGAGAAATATGGAACACAGG TGG (pTM55-FE*)
RAB58	*pkal-1(reb21)* X	AAACTATTGGGCACTTTCGG AGG (pTM55-FE*)
		CCCAGAATCCAGATGCATGG TGG (pTM55-FE*)
		TCTTGTGGACATATTCTGCC AGG (pTM55-FE*)
RAB59	*pkal-1(reb28)* X	AAACTATTGGGCACTTTCGG AGG (pTM55-FE*)
		CCCAGAATCCAGATGCATGG TGG (pTM55-FE*)
		TCTTGTGGACATATTCTGCC AGG (pTM55-FE*)
RAB60	C32E8.6*(reb23)* I	CTTCCATTCTTCCATGCGGG TGG (pTM55-FE*)
		GACGTGATCCGGAAAGTGGA GGG (pTM55-FE*)
RAB61	C32E8.6*(reb24)* I	CTTCCATTCTTCCATGCGGG TGG (pTM55-FE*)
		GACGTGATCCGGAAAGTGGA GGG (pTM55-FE*)
RAB89	*pks-1(reb22[A$_1$_G7106E])* X	AGGGACACCTGTTGAGCCAC TGG (pTM55-FE*)
RAB103	*pks-1(reb29[PCP$_2$_S7463A])* X	AGCAATTTGAATAGCATTGA GGG (pTM55-FE*)
RAB109	*nrps-1(reb31[A$_3$_ G2337D])* III	rCrUrUrCrGrGrUrGrUrArCrCrCrGrUrUrGrUrUrC
		rGrUrUrUrUrArGrArGrCrUrArUrGrCrU
RAB110	*nrps-1(reb32[A$_2$_ G964D])* III	rGrCrUrUrArCrGrUrCrArCrCrUrCrArArCrArUrC
		rGrUrUrUrUrArGrArGrCrUrArUrGrCrU

* pTM55-FE is a modified version of pTM55 (a gift of Patrick McGrath) and was mutated to enable a higher level of recognition efficiency[7] by Cas9.

Supplementary Table 7. Repair templates used for CRISPR-Cas9 in this study.

Strain	Genotype (alleles)	Repair template*
RAB51	*nrps-1(reb8[ACP$_7$_S307V])* III	ATGAAGTTGAAACCACTCCTCTACCATACCTCGGAATCGACGTCTTAAGACTCGCCGAGCTGGAGTACCACGTGGCTAGT (underlined: AatII; S307V: TCC»GTC)
RAB52	*pks-1(reb9[C$_1$_H6685A])* X	TGATAACAGTCGAATTCACATCGTTTCAATCAGCATGCAATTTTAACTGATGGTTGGTCAATGACTGTTCTTTCTGACACTGT (underlined: SphI; H6685A: CAT»GCA)
RAB53	*nrps-1(reb10[C$_3$_H1486A])* III	CTGGATGAGTAGCAAAAATAAGTTATTGACAATTTCCATTCACGCATTAATCTGCGATGGTAGAAGCCTGCAGATTCTCGAG (underlined: AseI; H1486A: CAC»GCA)
RAB54	*pks-1(reb11[TE$_1$_S7593A])* X	TGCCGCACATGCCGGAAACAAGAGAATCTTCGTTATGGGGCATGCGATGGGTGGAATAATGAGTCGCGAAATAGTGGCTGAGCTCAAAAT (underlined: SphI; S7593A: TCG»GCG)
RAB55	*nrps-1(reb12[TE$_2$_S2803A])* III	GTGCTGAAAATATTGAAACCTCTAAATTGGTGTTCATTGGCGCCGCTAGCGCTGGTACTTTTGCATTTTCCACGTCACAACTTTTTG (underlined: NheI; S2803A: TCG»GCT)
RAB56	*pks-1(reb13[TE$_1$_S7593C]; reb14[TE$_1$_G7596A])* X	TGCCGCACATGCCGGAAACAAGAGAATCTTCGTTATGGGACATTGCATGGGCGCCATAATGAGTCGCGAAATAGTGGCTGAGCTCAAAAT (underlined: KasI; S7593C/G7596A: TCG» TGC/ GGA»GCC)
RAB89	*pks-1(reb22[A$_1$_G7106E])* X	AGAACTCAATTTGGAAGTATTTACTCCATATTCACCAGTGAGTCGACAGGTGTCCCTAAAGGAGTTTTGATGGCGGAACAGTCA(underlined: SalI; G7106E: GGC»GAG)
RAB103	*pks-1(reb29[PCP$_2$_S7463A])* X	GTGGCGCCGACAGATAAATTTGAAAGTATTGGTGGAAACGCGTTAAATGCTATTCAAATTGCTCATCGGTTGGCTGAAGAG(underlined: MluI; S7463A: TCC»GCG)
RAB109	*nrps-1(reb31[A$_3$_G2337D])* III	CACCGACTATTGTCTCTCCTACATTATCACAACTAGTGATAC**T**AC**T**GGTACACCGAAGTCGGTAGCAATCGGAGCGAAATC (bold: changed for crRNA binding purposes; underlined: previous BspEI site; G2337D; GGA»GAT)
RAB110	*nrps-1(reb32[A$_2$_G964D])* III	CAACAAATCCATCCTATACGACCTAGCTTACGTCACGTCGACA**AG**TGACAGCACTGGGACCCCGAAACTGGTTGGAACCTCATTTG (bold: changed for crRNA binding purposes; underlined: SalI; G964D; GGA»GAC)

* The underlined bases indicate restriction sites designed for screening worms for the desired mutations, and the bases labeled in red code for the amino acid that was mutated. Additional silent base changes made because they were necessary for creating or removing the restriction sites have not been indicated.

Supplementary Table 8. Primers for construction of transcriptional and translational reporter strains.

Strain	Genotype	Name and Sequence*
RAB72	*rebEx15*	nemt-1p_SalI_Fwd
		GCGCGTCGACCGAAAATCTCAAGTCTTGTCTTAA**
		nemt-1p_NotI_Rev
		CATGGCGGCCGCAGTGAATATGTGTTTACGAGTAAATG**
RAB73	*rebEx16*	pkal-1p_SalI_Fwd
		GCGCGTCGACGCTATAAATGGGTACCTGGCCGTAA**
		pkal-1p_NotI_Rev
		CATGGCGGCCGCCGTAGAGAAGAAACTGTGACAGTTC**
RAB74	*rebEx17*	C32E8.6p_SalI_Fwd
		GCGCGTCGACGTACCGGGAATCGAAAAATTGTCC**
		C32E8.6p_NotI_Rev
		CATGGCGGCCGCTCTTATTGTACAGAATGTTTCTTTCC**
RAB76	*rebEx19*	nemt-1SL2_SalI_Fwd
		GCGCGTCGACCGAAAATCTCAAGTCTTGTCTTAA***
		nemt-1SL2_NotI_Rev
		CATGGCGGCCGCAGTGAATATGTGTTTACGAGTAAATG***
RAB77	*rebEx20*	pkal-1SL2_PstI_Fwd
		CATGCTGCAGGCTATAAATGGGTACCTGGCCGTAA***
		pkal-1SL2_NotI_Rev
		CATGGCGGCCGCTCAATAGTACATTAGCCTATTCTTTTG***
RAB78	*rebEx21*	C32E8.6SL2_SalI_Fwd
		GCGCGTCGACGTACCGGGAATCGAAAAATTGTCC***
		C32E8.6SL2_NotI_Rev
		CATGGCGGCCGCTCAATGCAACATATGACGGACTAG***
RAB81	*rebEx22*	Y71H2B.1SL2_PstI_Fwd
		CATGCTGCAGCCAGATGAAGAAAACGATGGAACT***
		Y71H2B.1SL2_NotI_Rev
		CATGGCGGCCGCTTAAACCCATCCCTCTGGAGGATT***
RAB82	*rebEx23*	C24A3.4SL2_SalI_Fwd
		GCAGGTCGACGGTCTCTACAGTGATGACACTCATT***
		C24A3.4SL2_NotI_Rev
		CATGGCGGCCGCTCACAGCTTGGACCGCGCCGCAAA***

*The underlined bases indicate restriction sites used for plasmid construction.

**Used to amplify gene promoter for insertion into pPD114.108.

***Used to promoter and gene from genomic DNA for insertion into pBS77-SL2-mCherry.

Supplementary Table 9. Primers for construction of protein expression plasmids.

Purpose	Name and sequence*
PKAL-1 expression vector	pkal-1_for<br>CATGCCATGGGGGCGAAATATTATCCAGAAAC<br>pkal-1_rev<br>CATGGCGGCCGCATAGTACATTAGCCTATTC
PKS-1_ACP_1 expression vector	pks-1_acp1_for<br>GCGCCCATGGGGCTTTCTGATGCGGAAATTGAGTC<br>pks-1_acp1_rev<br>CATGGCGGCCGCAGTTGTTGCTTTAGTAACTGGAAC
NRPS-1_ACP_7 expression vector	nrps-1_acp7_for<br>CATGCCATGGGGAGTGAAGACTCCGATGAAGAAGT<br>nrps-1_acp7_rev<br>CATGGCGGCCGCTCCGGACCCCAGCGCTTTCTCAC
Mutagenesis of PKAL-1	pkal-1K488A_1<br>GTCAAGTGGGGCCATTCAAAAGAATAG<br>pkal-1K488A_2<br>GATTTTGGCATCTCTTTTATAATTG
Mutagenesis of PKS-1_ACP_1	pks-1_acp1_1<br>ATATACCATGTACTGGTCTGATGCGGAAATTGAG<br>pks-1_acp1_2<br>CTCCTTCTTAAAGTTAAACAAAATTATTTC
Mutagenesis of NRPS-1_ACP_7	nrps-1_acp7_1<br>GTACAGTGAAGACTCCGATGAAG<br>nrps-1_acp7_2<br>CACATATATCTCCTTCTTAAAGTTAAAC

*The underlined bases indicate restriction sites used for plasmid construction.

References

1 Trautman, E. P., Healy, A. R., Shine, E. E., Herzon, S. B. & Crawford, J. M. Domain-targeted metabolomics delineates the heterocycle assembly steps of colibactin biosynthesis. *J Am Chem Soc* **139**, 4195-4201 (2017).

2 Robert, X. & Gouet, P. Deciphering key features in protein structures with the new ENDscript server. *Nucleic Acids Res* **42**, W320-324 (2014).

3 Balibar, C. J., Vaillancourt, F. H. & Walsh, C. T. Generation of D amino acid residues in assembly of arthrofactin by dual condensation/epimerization domains. *Chem Biol* **12**, 1189-1200 (2005).

4 Kelley, L. A., Mezulis, S., Yates, C. M., Wass, M. N. & Sternberg, M. J. The Phyre2 web portal for protein modeling, prediction and analysis. *Nat Protoc* **10**, 845-858 (2015).

5 Cao, J. *et al.* Comprehensive single-cell transcriptional profiling of a multicellular organism. *Science* **357**, 661-667 (2017).

6 Hutter, H. & Suh, J. GExplore 1.4: An expanded web interface for queries on *Caenorhabditis elegans* protein and gene function. *Worm* **5**, e1234659 (2016).

7 Chen, B. *et al.* Dynamic imaging of genomic loci in living human cells by an optimized CRISPR/Cas system. *Cell* **155**, 1479-1491 (2013).
