# Supplementary material for: Mapping the biosynthetic pathway of a hybrid polyketide-nonribosomal peptide in a metazoan
Source: Nat Commun. 2021 Aug 13;12:4912. doi: 10.1038/s41467-021-24682-9 (PMC8363725; doi:10.1038/s41467-021-24682-9)
Supplement: Supplementary file 2 — Reporting Summary [file 41467_2021_24682_MOESM2_ESM.pdf]

nature research

Corresponding author(s): Rebecca A. Butcher

Last updated by author(s): 05-28-2021

Reporting Summary

Nature Research wishes to improve the reproducibility of the work that we publish. This form provides structure for consistency and transparency in reporting. For further information on Nature Research policies, see our Editorial Policies and the Editorial Policy Checklist.

Statistics

For all statistical analyses, confirm that the following items are present in the figure legend, table legend, main text, or Methods section.

n/a	Confirmed	
☐	☒	The exact sample size (n) for each experimental group/condition, given as a discrete number and unit of measurement
☐	☒	A statement on whether measurements were taken from distinct samples or whether the same sample was measured repeatedly
☒	☐	The statistical test(s) used AND whether they are one- or two-sided<br>*Only common tests should be described solely by name; describe more complex techniques in the Methods section.*
☒	☐	A description of all covariates tested
☒	☐	A description of any assumptions or corrections, such as tests of normality and adjustment for multiple comparisons
☐	☒	A full description of the statistical parameters including central tendency (e.g. means) or other basic estimates (e.g. regression coefficient) AND variation (e.g. standard deviation) or associated estimates of uncertainty (e.g. confidence intervals)
☒	☐	For null hypothesis testing, the test statistic (e.g. F, t, r) with confidence intervals, effect sizes, degrees of freedom and P value noted<br>*Give P values as exact values whenever suitable.*
☒	☐	For Bayesian analysis, information on the choice of priors and Markov chain Monte Carlo settings
☒	☐	For hierarchical and complex designs, identification of the appropriate level for tests and full reporting of outcomes
☒	☐	Estimates of effect sizes (e.g. Cohen's d, Pearson's r), indicating how they were calculated

Our web collection on statistics for biologists contains articles on many of the points above.

Software and code

Policy information about availability of computer code

Data collection	No software used
Data analysis	GraphPad Prism 8, ChemStation B.04.03 (Agilent), Compass DataAnalysis 4.4 SR1 (Bruker), flexAnalysis 3.4 (Bruker), Clustal Omega 1.2.4, ESPript 3.0, PyMOL 2.3.3, BLAST+ 2.11.0, antiSMASH 6.0, SWISS-MODEL, Phyre2

For manuscripts utilizing custom algorithms or software that are central to the research but not yet described in published literature, software must be made available to editors and reviewers. We strongly encourage code deposition in a community repository (e.g. GitHub). See the Nature Research guidelines for submitting code & software for further information.

Data

Policy information about availability of data

All manuscripts must include a data availability statement. This statement should provide the following information, where applicable:

- Accession codes, unique identifiers, or web links for publicly available datasets
- A list of figures that have associated raw data
- A description of any restrictions on data availability

The LC–MS/MS data for 3, 4, 5, 6, 7, and 8 have been deposited in the MetaboLights database under accession code MTBLS2313 [https://www.ebi.ac.uk/metabolights/MTBLS2313]. Source data are provided with this paper. All other data supporting the findings of this study are available within the article and its Supplementary Information files or from the corresponding author upon request.

Field-specific reporting

Please select the one below that is the best fit for your research. If you are not sure, read the appropriate sections before making your selection.

☒ Life sciences ☐ Behavioural & social sciences ☐ Ecological, evolutionary & environmental sciences

For a reference copy of the document with all sections, see nature.com/documents/nr-reporting-summary-flat.pdf

Life sciences study design

All studies must disclose on these points even when the disclosure is negative.

Sample size	The samples sizes were chosen based on prior studies that showed significant effects with similar sample sizes (e.g., Zhang,X. et al., PNAS, 2015; Zhang, X. et al., ACS Chem. Biol., 2018; Zhou, Y. et al., eLife, 2018).
Data exclusions	No data from samples were excluded.
Replication	The number of replication were stated in the paper. All attempts were independent for at least two or three times. All attempts at replication were successful.
Randomization	No randomization was used so this is not relevant to our study. Worm experiments were done on bulk populations so it does not make sense to talk about randomization.
Blinding	No blinding was done. LC/MS traces were analyzed in an automated fashion such that blinding was not necessary.

Reporting for specific materials, systems and methods

We require information from authors about some types of materials, experimental systems and methods used in many studies. Here, indicate whether each material, system or method listed is relevant to your study. If you are not sure if a list item applies to your research, read the appropriate section before selecting a response.

Materials & experimental systems

n/a	Involved in the study	
☒	☐	Antibodies
☒	☐	Eukaryotic cell lines
☒	☐	Palaeontology and archaeology
☐	☒	Animals and other organisms
☒	☐	Human research participants
☒	☐	Clinical data
☒	☐	Dual use research of concern

Methods

n/a	Involved in the study	
☒	☐	ChIP-seq
☒	☐	Flow cytometry
☒	☐	MRI-based neuroimaging

Animals and other organisms

Policy information about studies involving animals; ARRIVE guidelines recommended for reporting animal research

Laboratory animals	The animal model Caenorhabditis elegans species was used in this study. Related mutants were obtained from Caenorhabditis Genetics Center (CGC) or generated by CRISPR-Cas9. Details of all strains in this study are available in the Supplementary Information. All C. elegans cultures were of mixed larval stages and contained both hermaphrodites and males at their natural frequency.
Wild animals	No wild animals were used in this study.
Field-collected samples	No field-collected samples were used in this study.
Ethics oversight	This study did not require ethical approval.

Note that full information on the approval of the study protocol must also be provided in the manuscript.

April 2020
